# Multi-ancestry transcriptome-wide association analyses yield insights into tobacco use biology and drug repurposing

**DOI:** 10.1038/s41588-022-01282-x

**Published:** 2023-01-26

**Authors:** Fang Chen, Xingyan Wang, Seon-Kyeong Jang, Bryan C. Quach, J. Dylan Weissenkampen, Chachrit Khunsriraksakul, Lina Yang, Renan Sauteraud, Christine M. Albert, Nicholette D. D. Allred, Donna K. Arnett, Allison E. Ashley-Koch, Kathleen C. Barnes, R. Graham Barr, Diane M. Becker, Lawrence F. Bielak, Joshua C. Bis, John Blangero, Meher Preethi Boorgula, Daniel I. Chasman, Sameer Chavan, Yii-Der I. Chen, Lee-Ming Chuang, Adolfo Correa, Joanne E. Curran, Sean P. David, Lisa de las Fuentes, Ranjan Deka, Ravindranath Duggirala, Jessica D. Faul, Melanie E. Garrett, Sina A. Gharib, Xiuqing Guo, Michael E. Hall, Nicola L. Hawley, Jiang He, Brian D. Hobbs, John E. Hokanson, Chao A. Hsiung, Shih-Jen Hwang, Thomas M. Hyde, Marguerite R. Irvin, Andrew E. Jaffe, Eric O. Johnson, Robert Kaplan, Sharon L. R. Kardia, Joel D. Kaufman, Tanika N. Kelly, Joel E. Kleinman, Charles Kooperberg, I-Te Lee, Daniel Levy, Sharon M. Lutz, Ani W. Manichaikul, Lisa W. Martin, Olivia Marx, Stephen T. McGarvey, Ryan L. Minster, Matthew Moll, Karine A. Moussa, Take Naseri, Kari E. North, Elizabeth C. Oelsner, Juan M. Peralta, Patricia A. Peyser, Bruce M. Psaty, Nicholas Rafaels, Laura M. Raffield, Muagututi’a Sefuiva Reupena, Stephen S. Rich, Jerome I. Rotter, David A. Schwartz, Aladdin H. Shadyab, Wayne H-H. Sheu, Mario Sims, Jennifer A. Smith, Xiao Sun, Kent D. Taylor, Marilyn J. Telen, Harold Watson, Daniel E. Weeks, David R. Weir, Lisa R. Yanek, Kendra A. Young, Kristin L. Young, Wei Zhao, Dana B. Hancock, Bibo Jiang, Scott Vrieze, Dajiang J. Liu

**Affiliations:** 1 Department of Public Health Sciences, Penn State College of Medicine, Hershey, PA USA; 2 Department of Psychology, University of Minnesota, Minneapolis, MN USA; 3 RTI International, Research Triangle, NC USA; 4 Department of Genetics, University of Pennsylvania, Philadelphia, PA USA; 5 Department of Psychology, Penn State College of Medicine, Hershey, PA USA; 6 Deparment of Bioinformatics and Genomics, Penn State College of Medicine, Hershey, PA USA; 7 Department of Cardiology, Cedars-Sinai Medical Center, Los Angeles, CA USA; 8 Division of Preventive Medicine, Brigham and Women’s Hospital, Boston, MA USA; 9 Department of Biochemistry, Wake Forest School of Medicine, Winston-Salem, NC USA; 10 College of Public Health, University of Kentucky, Lexington, KY USA; 11 Duke Molecular Physiology Institute, Duke University Medical Center, Durham, NC USA; 12 Department of Medicine, Duke University Medical Center, Durham, NC USA; 13 Duke Comprehensive Sickle Cell Center, Duke University Medical Center, Durham, NC USA; 14 Colorado Center for Personalized Medicine, University of Colorado Anschutz Medical Center, Aurora, CO USA; 15 Department of Medicine, Columbia University Medical Center, New York, NY USA; 16 Department of Medicine, Johns Hopkins University School of Medicine, Baltimore, MD USA; 17 Department of Epidemiology, School of Public Health, University of Michigan, Ann Arbor, MI USA; 18 Department of Medicine, Cardiovascular Health Research Unit, University of Washington, Seattle, WA USA; 19 Department of Human Genetics, University of Texas Rio Grande Valley School of Medicine, Brownsville, TX USA; 20 South Texas Diabetes and Obesity Institute, University of Texas Rio Grande Valley School of Medicine, Brownsville, TX USA; 21 Harvard Medical School, Boston, MA USA; 22 Department of Pediatrics, Institute for Translational Genomics and Population Sciences, Lundquist Institute for Biomedical Innovation at Harbor-UCLA Medical Center, Torrance, CA USA; 23 Department of Internal Medicine, National Taiwan University Hospital, Taipei, Taiwan; 24 Department of Medicine, Jackson Heart Study, University of Mississippi Medical Center, Jackson, MS USA; 25 University of Chicago, Chicago, IL USA; 26 NorthShore University Health System, Evanston, IL USA; 27 Department of Medicine, Division of Biostatistics and Cardiovascular Division, Washington University School of Medicine, St. Louis, MO USA; 28 Department of Environmental and Public Health Sciences, College of Medicine, University of Cincinnati, Cincinnati, OH USA; 29 Institute for Social Research, Survey Research Center, University of Michigan, Ann Arbor, MI USA; 30 Computational Medicine Core at Center for Lung Biology, Division of Pulmonary, Critical Care and Sleep Medicine, University of Washington, Seattle, WA USA; 31 Department of Medicine, University of Mississippi Medical Center, Jackson, MS USA; 32 Department of Epidemiology (Chronic Disease), School of Public Health, Yale University, New Haven, CT USA; 33 Department of Epidemiology, Tulane University School of Public Health and Tropical Medicine, New Orleans, LA USA; 34 Channing Division of Network Medicine, Brigham and Women’s Hospital, Boston, MA USA; 35 Division of Pulmonary and Critical Care Medicine, Brigham and Women’s Hospital, Boston, MA USA; 36 Department of Epidemiology, Colorado School of Public Health, University of Colorado Anschutz Medical Campus, Aurora, CO USA; 37 Institute of Population Health Sciences, National Health Research Institutes, Zhunan, Taiwan; 38 The Population Sciences Branch, Division of Intramural Research, National Heart, Lung, and Blood Institute, National Institutes of Health, Bethesda, MD USA; 39 The Framingham Heart Study, Framingham, MA USA; 40 Lieber Institute for Brain Development, Baltimore, MD USA; 41 Department of Psychiatry and Behavioral Sciences, Johns Hopkins University School of Medicine, Baltimore, MD USA; 42 Department of Neurology, Johns Hopkins University School of Medicine, Baltimore, MD USA; 43 Department of Epidemiology, University of Alabama at Birmingham, Birmingham, AL USA; 44 Department of Mental Health and Department of Biostatistics, Johns Hopkins Bloomberg School of Public Health, Baltimore, MD USA; 45 Department of Human Genetics and Department of Neuroscience, Johns Hopkins University School of Medicine, Baltimore, MD USA; 46 Department of Epidemiology and Population Health, Albert Einstein College of Medicine, The Bronx, NY USA; 47 Public Health Sciences Division, Fred Hutchinson Cancer Research Center, Seattle, WA USA; 48 Departments of Environmental & Occupational Health Sciences, Medicine, and Epidemiology, University of Washington Seattle, Seattle, WA USA; 49 Fred Hutchinson Cancer Center, Seattle, WA USA; 50 Department of Internal Medicine, Division of Endocrinology and Metabolism, Taichung Veterans General Hospital, Taichung, Taiwan; 51 Department of Population Medicine, Harvard Pilgrim Health Care, Boston, MA USA; 52 Center for Public Health Genomics, University of Virginia, Charlottesville, VA USA; 53 Division of Cardiology, George Washington University School of Medicine and Health Sciences, Washington, DC USA; 54 Department of Biomedical Sciences, Penn State College of Medicine, Hershey, PA USA; 55 Department of Epidemiology, International Health Institute, Brown University School of Public Health, Providence, RI USA; 56 Department of Human Genetics and Department of Biostatistics, University of Pittsburgh, Pittsburgh, PA USA; 57 Penn State Huck Institutes of Life Sciences, Penn State College of Medicine, University Park, PA USA; 58 Ministry of Health, Government of Samoa, Apia, Samoa; 59 Department of Epidemiology, Gillings School of Global Public Health, University of North Carolina at Chapel Hill, Chapel Hill, NC USA; 60 Department of Epidemiology, University of Washington, Seattle, WA USA; 61 Department of Health Systems and Population Health, University of Washington, Seattle, WA USA; 62 Department of Genetics, University of North Carolina at Chapel Hill, Chapel Hill, NC USA; 63 Lutia I Puava Ae Mapu I Fagalele, Apia, Samoa; 64 Department of Medicine, University of Colorado, Aurora, CO USA; 65 Herbert Wertheim School of Public Health and Human Longevity Science, University of California San Diego, La Jolla, CA USA; 66 Taipei Veterans General Hospital, Taipei, Taiwan; 67 Faculty of Medical Sciences, University of the West Indies, Cave Hill Campus, Barbados

**Keywords:** Transcriptomics, Software

## Abstract

Most transcriptome-wide association studies (TWASs) so far focus on European ancestry and lack diversity. To overcome this limitation, we aggregated genome-wide association study (GWAS) summary statistics, whole-genome sequences and expression quantitative trait locus (eQTL) data from diverse ancestries. We developed a new approach, TESLA (multi-ancestry integrative study using an optimal linear combination of association statistics), to integrate an eQTL dataset with a multi-ancestry GWAS. By exploiting shared phenotypic effects between ancestries and accommodating potential effect heterogeneities, TESLA improves power over other TWAS methods. When applied to tobacco use phenotypes, TESLA identified 273 new genes, up to 55% more compared with alternative TWAS methods. These hits and subsequent fine mapping using TESLA point to target genes with biological relevance. In silico drug-repurposing analyses highlight several drugs with known efficacy, including dextromethorphan and galantamine, and new drugs such as muscle relaxants that may be repurposed for treating nicotine addiction.

## Abstract (web summary)

A multi-ancestry transcriptome-wide association study using an optimal linear combination of association statistics provides insights into tobacco use biology and suggests opportunities for drug repurposing.

## Main

Cigarette smoking is a major heritable risk factor for human diseases. The availability of large datasets has enabled a breakthrough in the genetics of smoking addiction, with >400 loci discovered to date^1^. Although some of these associations point to genes and pathways of known biological importance, including the nicotinic receptor and dopaminergic signaling pathway genes^1^, the underlying mechanisms for most of the identified loci are unknown. On top of this, the genetic architecture of tobacco use outside of European populations remains understudied. In the present study, we combined GWAS datasets totaling 1.3 million individuals: 1.2 million from the GWAS and Sequencing Consortium of Alcohol and Nicotine use (GSCAN) and 150,000 diverse ancestries from the Trans-Omics Precision Medicine (TOPMed)^2^ to further empower gene discovery and elucidate the genetic architecture of smoking behavior.

Dissecting the mechanisms of GWAS hits for tobacco use is crucial to understand the etiology of nicotine addiction and related disease outcomes. TWAS approaches (for example, FUSION^3^, TIGAR^4^, PrediXcan^5^ and UTMOST^6^) use eQTLs to predict gene expression levels in silico, which the method then uses to identify genes associated with the phenotype of interest. Various TWAS methods have been widely applied to different complex traits to understand the functional consequences of regulatory variations^7–9^.

TWAS in its original form requires GWAS and eQTL data to be from matched ancestries. Direct integration of eQTLs with GWAS data from nonmatched ancestries (for example, integrating European-derived eQTLs with non-European GWASs) was shown to have suboptimal power^10^. The results may also be difficult to interpret because causal variants underlying GWAS hits or eQTLs may differ between ancestries. An alternative strategy is to use ancestry-matched eQTL data from disease-relevant tissues and perform TWAS separately for each ancestry (which we call MATCH-TWAS). MATCH-TWAS may be difficult or even impossible to implement in practice because eQTL data may not be broadly available for disease-relevant tissues in non-European ancestries. In addition, because most causal variants have been observed to be consistent across ancestries^11–13^, MATCH-TWAS can suffer from substantial power loss by using only the GWAS data from the matched ancestry, due simply to smaller sample size. Another possible strategy is to ignore ancestral differences and perform TWAS using GWAS fixed effect (FE) or random effect (RE) meta-analysis results combining different ancestries (FE-TWAS and RE-TWAS). FE-TWAS and RE-TWAS do not fully leverage ancestral differences in phenotypic effect sizes and linkage equilibrium (LD) patterns, which also leads to suboptimal power.

Given the lack of sizable eQTL datasets from disease-relevant tissues in a matched ancestry, it is important to develop methods to optimally integrate an existing eQTL dataset from a given ancestry (European or any ancestry) in a multi-ancestry meta-analysis. To achieve this goal, we developed a new method, TESLA, which exploits shared phenotypic effects across ancestries and accommodates between-ancestry genetic effects, and consistently improves power over existing methods. We identify many more gene-level associations than alternative methods, such as MATCH-TWAS and FE-TWAS. We also performed fine mapping, enrichment and drug-repurposing analyses for TWAS hits to learn new biology and gain clinical insights related to tobacco use phenotypes.

## Results

### Method overview

For all presentations, we call the genetic effects on GWAS phenotypes ‘phenotypic effects’ and the effect of gene expression the ‘eQTL effect’. TWAS was originally developed to integrate eQTL and GWAS datasets derived from matched ancestries^5^. Specifically, it first builds gene expression prediction models using eQTL datasets that measure both gene expression levels and genotypes and obtains weights on eQTL SNPs (*w*_*j*_). The eQTL weights are then used to calculate a weighted sum of phenotypic effect estimates (which we denote as *b*_*j*_ for the effect of variant *j*) for gene-level association tests. When adapting TWAS to integrate European eQTL data with non-European GWAS data, power loss was observed empirically^10^, but the theoretical reason behind the power loss was not well established.

In the present study, we propose a proportionality condition under which trans-ancestry TWAS attains its optimal power. Specifically, the proportionality condition states that TWAS has optimal power if the phenotypic effects and eQTL weights from the gene expression prediction model are proportional to each other. This condition is satisfied when the eQTL SNPs influence phenotypes via their regulatory effects, that is, ${{{\mathrm{SNP}}}}j\mathop{\longrightarrow}\limits^{{w_j}}{{{\mathrm{Expression}}}}\mathop{\longrightarrow}\limits^{c}{{{\mathrm{Phenotype}}}}$, where *w*_*j*_ is the eQTL effect of SNP *j* from the gene expression prediction model and *c* is the effect of genetically regulated gene expressions on the phenotypes. The phenotypic effect of variant *j* satisfies $\beta _j = w_jc$. When the eQTL and GWAS data come from the same ancestry and the phenotypic and eQTL effect heterogeneities between studies are modest, the proportionality condition is expected to hold. However, when integrating non-European GWASs with European eQTL datasets, this proportionality condition can be violated and the power for TWASs is suboptimal because the set of causal variants and their phenotypic effects may differ across different ancestries. Motivated by this proportionality condition, we developed an improved TWAS method, TESLA, that optimally integrates a given eQTL dataset with a multi-ancestry GWAS. TESLA consists of three key steps.

First, TESLA models phenotypic effects across ancestries using meta-regression, which takes phenotypic effect estimates, standard deviations and genome-wide allele frequency principal components (PCs, as a proxy for ancestry) as input. We estimate ancestry using genetic PC analysis on per-study allele frequencies, although other methods may also be used. When no PC is included, the model is equivalent to a fixed-effects meta-analysis; when one or more PCs are included, the meta-regression coefficients quantify the extent of SNP effect heterogeneity as a function of ancestry. For example, in the present study, the first PC separates cohorts of individuals with recent African–American ancestries (Supplementary Fig. 4). The regression coefficient for the first PC will estimate how much the phenotypic effect varies between samples of African and non-African ancestry. This model jointly analyzes different ancestries, which maximizes the sample size and improves the phenotypic effect estimates. To account for the unknown extent of phenotypic effect heterogeneities, we fit multiple different meta-regression models with varying numbers of PCs. The method synthesizes the phenotypic effect estimates from different meta-regression models in the third step for TWASs.

Next, for each fitted model, we estimate phenotypic effects in the ancestry that match the eQTL dataset. For cohorts from ancestries that do not match the eQTL dataset, their phenotypic effect will be projected to allele frequency PCs of the eQTL dataset and then meta-analyzed with other cohorts. The resulting estimates benefit from the contribution of cohorts of all ancestries and satisfy the proportionality condition, as long as the phenotypic effects are mediated by the genetically regulated gene expressions and effect heterogeneity in the same ancestry is modest. The performance of TWASs using the eQTL weights and estimated phenotypic effects in the matched ancestry thus yields optimal power.

Finally, TESLA combines the TWAS results based on multiple meta-regression models using a minimal *P*-value method to attain robust results. We also assessed whether TESLA hits are enriched in pathways or tissues and identified candidate drugs that may be repurposed for smoking cessation. We provide details in Methods and Supplementary Text.

We perform extensive simulation to evaluate the proposed method and compare them with FE-TWASs, RE-TWASs and EURO-TWASs using meta-analysis results from METASOFT (Code availability). We show that TESLA consistently outperforms or performs competitively compared with other methods across all scenarios (Supplementary Text and Supplementary Tables 1 and 2). In fact, TESLA is the only method that performs consistently well. Given that the genetic effects are often unknown in practice, TESLA is a clear favorite in real applications.

### TESLA improves gene discovery in diverse ancestries

**Fig. 1:**
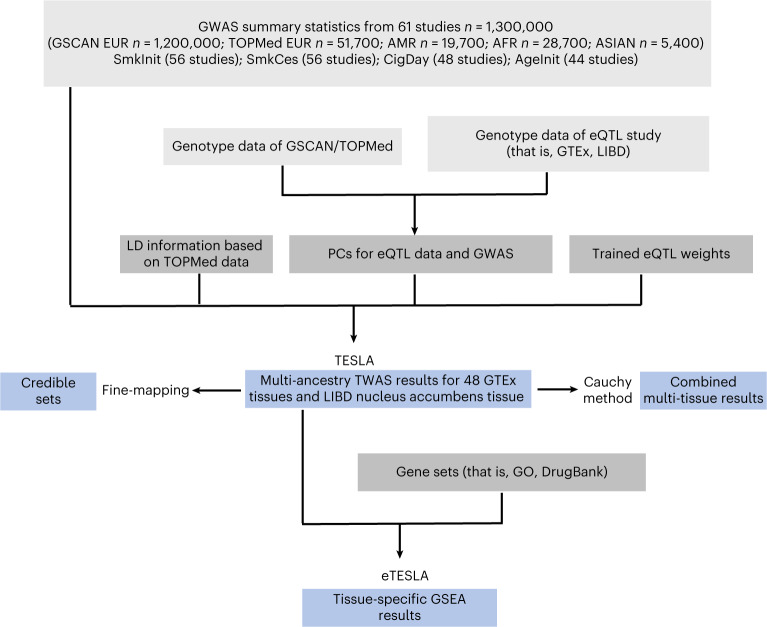
Schematic description of the TESLA method. TESLA uses meta-regression to model phenotypic effect estimates as functions of the PCs of genome-wide allele frequencies from each cohort. For a given gene expression prediction model generated from an eQTL dataset, we use TESLA to more accurately estimate phenotypic effects, then use them to perform TWASs and attain optimal power. We also performed fine mapping and enrichment analysis using the TESLA results (which we call eTESLA).

We applied TESLA to summary-level association statistics derived from 61 cohorts in GSCAN and TOPMed studies of 4 smoking traits including smoking initiation (SmkInit, binary trait of smoker versus nonsmoker), cigarettes per day (CigDay, continuous outcome), smoking cessation (SmkCes, binary outcome comparing current versus former smokers) and age of smoking initiation (AgeInit, continuous outcome of the age of starting regular smoking) (Supplementary Table 3). Details of phenotype definitions can be found in Methods and Supplementary Text. PrediXcan weights of 48 tissues from samples of European ancestry in GTEx (v.7) (Genotype-Tissue Expression) were used. TESLA was applied to analyze gene–phenotype associations in each tissue separately. All statistical tests that we performed and the reported *P* values are two sided, unless stated otherwise. Tissue-specific TESLA results were also combined using the Cauchy combination test^14^ to obtain a *P* value of a multi-tissue TWAS for each gene. A schematic description of the TESLA analysis flow is shown in Fig. 1.

**Fig. 2:**
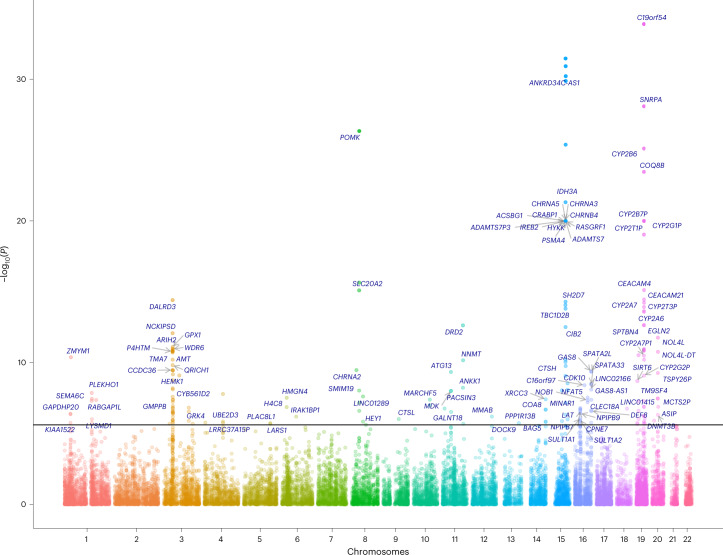
Manhattan plot for multi-tissue TESLA results using GTEx for CigDay phenotype. For each chromosome, we labeled the fine-mapped genes with posterior inclusion probability (PIP) > 0.9 (with *P* < 2.5 × 10^−6^). If more than ten genes were significant for a chromosome, only the top ten genes were labeled. The Manhattan plot for other traits can be found in Supplementary Fig. 2. All *P* values are two sided. We have now labeled the fine-mapped genes with PIP > 0.9 in the Manhattan plot. For smoking initiation trait, there are a large number of fine-mapped signals, so we labeled only ten genes per chromosome with the largest PIP values.

TESLA results produced well-calibrated genomic control values (Supplementary Fig. 1) in each GTEx tissue and phenotype. A total of 4,475 gene × trait associations (across 48 tissues, 1,389 unique genes in total) of 4 smoking traits were identified by TESLA with *P* values <2.5 × 10^−6^ (Bonferroni’s threshold for testing up to 20,000 expressed genes), which was 6.9%, 504% and 12.5% more than FE-TWAS, RE-TWAS and EURO-TWAS, respectively (Table 1, Fig. 2 and Supplementary Figs. 2 and 3). Although 87% of the GWAS samples were of European ancestry, we still noted considerable improvement in power from TESLA, which corroborated the simulation results. Among these results, 783 gene × trait associations (384 unique genes) were identified in 13 brain tissues, including the amygdala, anterior cingulate cortex, caudate, cerebellar hemisphere, cerebellum, cortex, frontal cortex, hippocampus, hypothalamus, nucleus accumbens, putamen, brain spinal cord (cervical C1) and substantia nigra (Supplementary Table 4).

**Table 1:** TESLA identified substantially more loci and new loci than FE-TWASs, RE-TWASs and EURO-TWASs using GTEx data and PrediXcan weights

Genes identified across all the tissues				
Trait	TESLA	FE-TWAS	RE-TWAS	EURO-TWAS
SmkInit	3,066 (908, 193)	2,916 (852, 168)	218 (84, 12)	2,729 (795, 132)
SmkCes	476 (155, 19)	414 (136, 16)	33 (19, 4)	428 (144, 16)
CigDay	840 (276, 46)	793 (248, 29)	482 (143, 31)	793 (229, 26)
AgeInit	93 (50, 15)	64 (38, 8)	8 (7, 3)	29 (21, 2)
Total	4475 (1,389, 273)	4187 (1,274, 221)	741 (147, 50)	3979 (1,189, 176)

Genes with two-sided TWAS *P* values <2.5 × 10^−6^ were deemed statistically significant. A gene × trait association was considered new if it was >1 × 10^6^ bp away from previously reported GWAS hits. The number of gene × trait associations, the number of unique gene × trait associations (that is, the gene × trait association that appears in multiple tissues are counted only once) and new associations are shown for each TWAS method. The numbers in parentheses are unique gene and new gene counts, respectively

Among the TESLA-identified genes, 15, 193, 19 and 46 were new for AgeInit, SmkInit, SmkCes and CigDay, respectively, which are >1 × 10^6^ base pairs (bp) away from known GWAS sentinel variants (Supplementary Table 5). The number of new genes identified by TESLA was also 23.5% and 55.1% more than FE-TWAS and EURO-TWAS, respectively. We also counted the number of new loci where we considered genes within 1 × 10^6^ bp of each other to be the same locus. A similar advantage remains where the number of new loci identified by TESLA is 20.5% and 32.5% more than FE-TWASs and EURO-TWASs, respectively. The improvements over FE-TWAS showcase the advantage of the TESLA method, whereas the advantage over EURO-TWAS is probably attributable to the addition of non-European samples. The advantage of TESLA was maintained when a more stringent *P*-value threshold was used (that is, 5.0 × 10^−8^, Bonferroni’s threshold for testing 20,000 genes among 48 tissues) (Supplementary Table 6).

The number of significant associations in each tissue was influenced by both the tissue relevance for the trait and the sample size of the eQTL dataset. Although brain tissues are known to be involved in tobacco use phenotypes, we did not observe an increased number of associated genes in brain tissues, possibly because the small sample sizes of brain tissue eQTL datasets lead to limited power for predicting gene expression in silico. On the other hand, we typically found a larger number of gene × trait associations in tissues with larger eQTL sample sizes, with TWASs in whole blood yielding the largest number of associations (Supplementary Fig. 3).

Similar patterns were observed for TESLA analysis with nucleus accumbens eQTL data from the Lieber Institute for Brain Development (LIBD) Human Brain Repository, which contains a higher representation of non-European ancestry (*n* = 198; 53% of European and 47% with African ancestry) than GTEx (*n* = 114 for nucleus accumbens; overall 15% non-European ancestry). As the sample size of non-European ancestry GWASs is relatively small (AgeInit *n* = 11,626, CigDay *n* = 12,379, SmkCes *n* = 14,293, SmkInit *n* = 22,693), the number of gene × trait associations identified using African–American eQTL data is small, but a significant portion is replicated in the TWAS using European eQTLs (Supplementary Table 7). The advantage of TESLA over alternative TWAS methods widened even more using the African ancestry eQTL dataset, because the fraction of non-African ancestry GWAS samples is large. Across 4 smoking traits, TESLA identified 122 genes, which was 91% more than FE-TWAS (64 significant gene associations), the second-best method. On the other hand, AFR-TWAS that uses ancestry-matched African ancestry eQTL and GWAS data yielded much smaller numbers of genes, because only a small fraction of GWAS cohorts was of African ancestry. This showed that conducting TWASs using only ancestry-matched GWAS and eQTL datasets cannot overcome sample size limitations and thus they remain severely underpowered (Supplementary Table 8).

Based on TESLA results, we quantified the extent of phenotypic effect heterogeneity based on the models that yield minimal *P* values and show that 77% of the genes have homogeneous effects across ancestries. (Supplementary Text, Supplementary Figs. 4 and 5 and Supplementary Table 9). We also performed fine-mapping analysis and identified a number of genes with biological relevance (Supplementary Text, Supplementary Fig. 6 and Supplementary Table 10).

### Enrichment analysis highlighted key pathways

**Fig. 3:**
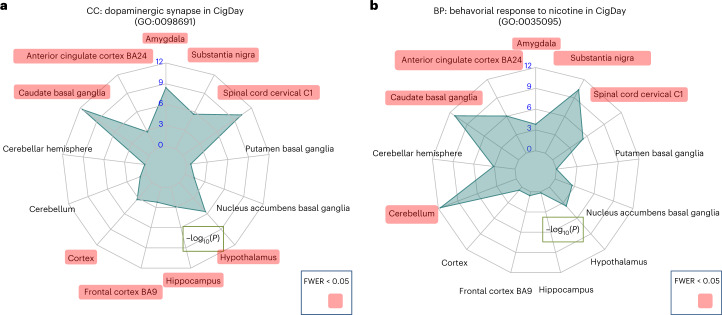
Key addiction-related pathways are ubiquitously enriched with TESLA hits in multiple brain tissues. We displayed TESLA enrichment *P* values (two sided) across 13 GTEx brain tissues using radar plots. **a**,**b**, The enrichment of TESLA hits for cigarettes per day for the dopaminergic synapse pathways (**a**) and the behavioral response to nicotine pathways (**b**). Gridlines in the radar plots indicate different levels of statistical significance. Each spoke represents a brain tissue and the length of the spoke represents the −log_10_(*P*) of enrichment. Brain tissues with significant enrichment *P* values after multiple testing corrections are shown in red. CC, cellular component; BP, biological process.

We used gene ontology (GO) enrichment analysis to find pathways, tissues and cell types relevant to tobacco use (Supplementary Table 11). Our enrichment analysis is based on the same idea as GWAS-based pathway analysis tools, such as MAGMA^15^, which leverage weighted regression to assess whether a given pathway is enriched with TWAS hits from a given tissue^16^. First, we identified a number of key pathways with known biological relevance to addiction that are ubiquitously enriched in multiple tissues. These pathways include neuromuscular synaptic transmission (GO:0007274), neurotransmitter catabolic process (GO:0042135), negative regulation of synaptic transmission, GABAergic (GO:0032229), Lewy body (GO:0097413) and dopaminergic synapse (GO:0098691) (Fig. 3a). Importantly, many tobacco-related pathways are consistently ranked among the top pathways (family-wise error rate (FWER) < 0.05) in the cerebellum, including neurotransmitter catabolic process (GO:0042135) for CigDay (*P* = 9.5 × 10^−11^), dopaminergic synapse (GO:0098691) for SmkInit (*P* = 1.2 × 10^−9^) and behavioral response to nicotine (GO:0035095) for CigDay (*P* = 2.3 × 10^−14^). This finding is consistent with increasing evidence showing that cerebellum functions extend beyond motor control and involve rewarding and addictive behaviors^17–22^.

**Fig. 4:**
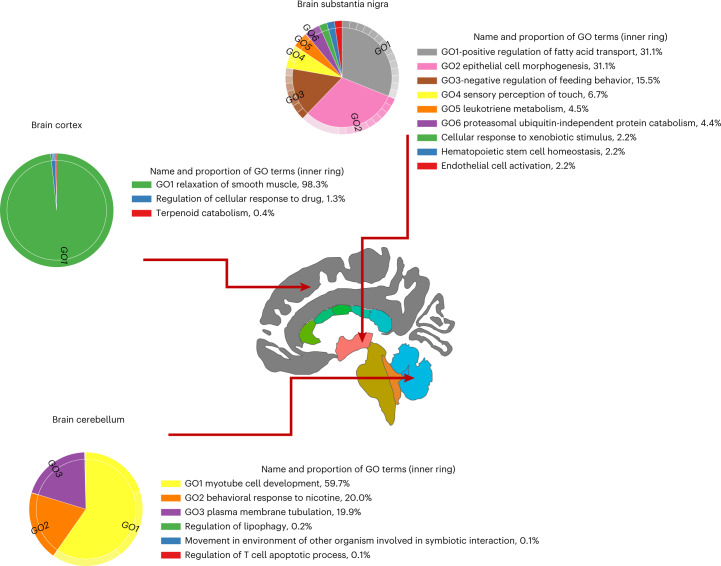
Different brain tissues are enriched with distinct pathways. We used REVIGO to reduce redundant GO terms and facilitate the visualization of enrichment results. We highlighted three brain regions (that is, cortex, substantia nigra and cerebellum) with distinct patterns of enrichment. For brain cortex, one GO term (relaxation of smooth muscle) accounts for 98.3% of the pathways enriched with TWAS hits, whereas, for substantia nigra and cerebellum, a diverse set of GO terms was enriched with TWAS hits. The brain figures are generated by R package ggseg^43^.

On the other hand, for most pathways, the enrichment patterns differ between traits and tissues, which implicated potentially different genetic architectures (Fig. 3b). To reduce the dimension of the data and reveal underlying biology, we clustered GO items using the REVIGO method^23^ (Code availability and Supplementary Fig. 7). For CigDay, the only dominant pathway category enriched with TWAS hits in cortex is ‘relaxation of smooth muscle’ (GO:0044557, *P* = 3.4 × 10^−7^, with weight = 98.3%), whereas there are more diverse GO items in substantia nigra, an important brain tissue for reward. The top GO terms enriched with TESLA hits include: ‘positive regulation of fatty acid transport’ (weight = 31.3%), ‘epithelial cell morphogenesis’ (weight = 31.1%), ‘negative regulation of feeding behavior’ (weight = 15.5%) and ‘sensory of touch’ (weight = 6.7%) (Fig. 4). The top GO terms have been implicated in substance use and addictive behaviors. For example, poly(unsaturated fatty acids) were known to influence psychiatric outcomes among drug users and food supplements for poly(unsaturated fatty acids) have been used to stabilize aggressive behaviors^24^. It is interesting that smoking is also known to reduce stress and have self-medication effects. In addition, the enriched GO term ‘negative regulation of feeding behavior’ is corroborated by many smoking-associated loci. These loci were implicated in feeding behavior due to their functions in reward processing^25^. Results from MAGMA enrichment analyses using samples of European ancestry were included as a comparison (Supplementary Table 12). Top hits from MAGMA remain significant in TESLA and show up in multiple tissues, whereas hits that are only significant in TESLA tend to be more tissue specific.

Finally, we incorporated single-cell RNA-sequencing (scRNA-seq) data from neurons in the mouse central nervous system to prioritize specific cell types related to tobacco use phenotypes^26^. We created cell-type-specific gene sets that consist of the top 10% most highly expressed genes specific to each cell type and tested whether they are enriched with TESLA hits (Supplementary Fig. 8 and Supplementary Table 13). We highlighted cholinergic and monoaminergic neurons (*P* = 4.9 × 10^−6^, FEWR < 0.01), as well as glutamatergic neuroblasts (*P* = 6.1 × 10^−6^, FWER < 0.01), as relevant cell types for CigDay in the cerebellum (Supplementary Fig. 8), which corroborated human brain transcriptomic data.

### Enrichment analysis identified drugs for repurposing

We created genes sets for targeted pathways of each drug in DrugBank^27^ and examined whether these drug target pathways were enriched with TESLA hits in 13 brain tissues from GTEx. We identified 102 putative drugs pathways under stringent Bonferroni’s threshold for testing 1,642 drugs (7.9 × 10^−7^) (Supplementary Table 14). As confirmation, we also included enrichment analysis based on MAGMA, a gene-based method that aggregates phenotype association results without incorporating eQTLs, using samples of European ancestry. Our results pointed to drugs with putative or known relevance to smoking cessation and suggested new drug classes that may be repurposed for treatment of smoking cessation (Table 2).

**Table 2:** Top drugs identified using enrichment analysis

Drug name	Indication	Smoking trait	Minimal *P* value and tissue types^a^	MAGMA^b^	Reference^c^
**Putative drug targets that may be repurposed for smoking cessation**					
Dextromethorphan	Coughing	CigDay	**3.3** **×** **10**^**−39**^ **(caudate basal ganglia)**	1.0 × 10^−4^	^32,41^
		SmkInit	**9.2** **×** **10**^**−15**^ **(brain spinal cord cervical C1)**	0.36	
		SmkCes	2.8 × 10^−4^ (brain spinal cord cervical C1)	**9.2** **×** **10**^**−9**^	
Ganaxolone	Seizure disorders (investigated)	CigDay	**1.3** **×** **10**^**−9**^ **(substantia nigra)**	0.05	^33^
		SmkInit	3.5 × 10^−3^ (cerebellum)	0.66	
		SmkCes	0.08 (caudate basal ganglia)	0.02	
Galantamine	Alzheimer’s disease	CigDay	**4.2** **×** **10**^**−73**^ **(substantia nigra)**	**7.7** **×** **10**^**−14**^	^34,42^
		SmkInit	1.3 × 10^−4^ (brain spinal cord cervical C1)	4.3 × 10^−3^	
		SmkCes	0.020 (cortex)	**3.4** **×** **10**^**−9**^	
**Clinical drugs identified**					
Nicotine	Smoking cessation	CigDay	**4.2** **×** **10**^**−71**^ **(substantia nigra)**	**4.3** **×** **10**^**−17**^	First-line therapy
		SmkInit	1.3 × 10^−5^ (hypothalamus)	0.01	
		SmkCes	0.03 (amygdala)	**5.8** **×** **10**^**−11**^	
Varenicline		CigDay	**4.8** **×** **10**^**−26**^ **(frontal cortex BA9)**	**5.6** **×** **10**^**−6**^	First-line therapy
		SmkInit	**9.2** **×** **10**^**−15**^ **(brain spinal cord cervical C1)**	5.9 × 10^−3^	
		SmkCes	2.8×10^−4^ (brain spinal cord cervical C1)	**8.2** **×** **10**^**−9**^	
Bupropion		CigDay	**9.0** **×** **10**^**−19**^ **(brain spinal cord cervical C1)**	0.62	First-line therapy
		SmkInit	**9.2** **×** **10**^**−15**^ **(brain spinal cord cervical C1)**	0.92	
		SmkCes	2.8 × 10^−4^ (brain spinal cord cervical C1)	0.05	
Cytisine		CigDay	**3.9** **×** **10**^**−132**^ **(frontal cortex BA9)**	**5.6** **×** **10**^**−6**^	Second-line therapy
		SmkInit	**9.2** **×** **10**^**−15**^ **(brain spinal cord cervical C1)**	5.9 × 10^−3^	
		SmkCes	2.8 × 10^−4^ (brain spinal cord cervical C1)	**8.2** **×** **10**^**−9**^	
Anxiolytic drugs (butalbital)^d^		CigDay	**4.8** **×** **10**^**−132**^ **(frontal cortex BA9)**	1.1 × 10^−3^	Second-line therapy
		SmkInit	4.9 × 10^−5^ (cerebellar hemisphere)	7.3 × 10^−3^	
		SmkCes	1.1 × 10^−3^ (caudate basal ganglia)	0.33	

Drug enrichment analysis of TESLA results implicates drugs with biological relevance and drugs that are being clinically evaluated. We created gene sets of drug target genes and tested whether these gene sets were enriched with TESLA hits. The most significant TESLA *P* values for enrichment analysis are shown and, as a comparison and validation, we also show enrichment analysis based on MAGMA for implicated drugs. Full results are available in Supplementary Table 14. All *P* values are two sided.

^a^The minimal *P* value in 13 brain tissues; significance Bonferroni’s corrected *P* values that are under 5% threshold and labeled bold.

^b^MAGMA using the GWAS signals; significant *P* values after Bonferroni’s correction are labeled bold.

^c^References where the candidate drugs were discussed.

Preliminary clinical/basic evidence/references support the drug repositioning.

^d^Complete enrichment results for anxiolytic drugs are shown in Supplementary Table 14.

First, as a positive control and confirmation of the validity of our approach, our enrichment analysis identified approved drugs, including varenicline, bupropion and cytisine, which are used as first- or second-line therapies for smoking cessation^28–31^.

Second, TESLA enrichment pointed to drugs with putative smoking cessation effects, which are being evaluated in clinical trials. For example, the target pathway of dextromethorphan^32^, a drug originally used to treat cough, is enriched with CigDay loci in anterior cingulate cortex BA24 (*P* = 3.28 × 10^−31^, FEWR < 0.01), caudate basal ganglia (*P* = 1.17 × 10^−39^, FEWR < 0.01) and cerebellum (*P* = 7.4 × 10^−39^, FEWR < 0.01). The drug target pathway for ganaxolone^33^, a drug used for seizure disorders, is enriched with CigDay loci in hippocampus (*P* = 2.2 × 10^−5^, FEWR < 0.01) and substantia nigra (*P* = 1.1 × 10^−9^, FEWR < 0.01).

Enrichment analysis also identified potential drugs for treating smoking addiction, which are supported by preliminary clinical evidence. For example, galantamine, a Food and Drug Administration-approved medication for the treatment of cognitive deficits associated with Alzheimer’s disease, increases synaptic acetylcholine levels by inhibiting acetylcholinesterase, an enzyme that breaks down acetylcholine. Galantamine also directly stimulates α7- and α4β2-nicotinic acetylcholine receptors (nAChRs) via its positive allosteric modulator actions^34^.

In addition to individual drugs, we also evaluated the potential of drug classes that can be repurposed for smoking cessation. To do so, we grouped all the identified drugs into 15 categories based on their indications (see Supplementary Text). The top drug group enriched with CigDay hits was muscle relaxants, which have established relevance to smoking. For example, γ-aminobutyric acid (GABA) β-agonist baclofen was shown to ameliorate nicotine- and drug-induced behavior in animals and humans. This could be due to their shared targets of nAChR pathways with smoking addiction. The other two largest drug groups were for the treatment of mental disorders and neurological drugs (Supplementary Fig. 9).

## Discussion

In the present study, we conducted a multi-ancestry TWAS using GWASs and whole-genome sequence data from 1.3 million individuals. Our TWAS results highlighted shared mechanisms with other substance use behaviors (for example, cocaine addiction) and psychiatric phenotypes (for example, pain sensitivity, depression and anxiety). Leveraging shared disease pathways, we identified drugs that may be repurposed for smoking cessation treatment, including dextromethorphan and galantamine, which are already being assessed in clinical trials. Given the tremendous public health burden that continues to be incurred by smoking, repurposing drugs for smoking cessation is extremely valuable, because it offers a potentially quicker and more cost-effective route to treatment than the development of new therapeutic targets.

Our work also made important methodological contributions. TESLA showed robust performance over other methods across different genetic architectures, which makes it a desirable choice in practice, because the true phenotypic effects across ancestries are unknown. TESLA improves power because it jointly analyzes samples from multiple ancestries, maximizes sample sizes and accommodates between-ancestry heterogeneities. The magnitude of increased power depends on the genetic architecture of the traits across ancestries. TELSA has the largest advantage when causal variants are shared between ancestries but have heterogeneous effects. Its performance is comparable to other well-performing methods when the effects are unique to European ancestry or homogeneous across ancestry groups. Importantly, the power improvement of TESLA over alternative methods tends to increase as a larger fraction of non-European samples is included. This ensures that TESLA will be even more useful because genetic studies are expanding to non-European populations, as part of the biomedical research community’s vision for precision health using genomics^35^.

TESLA uses allele frequency PCs to capture cohort ancestry differences^36^ (Supplementary Fig. 4), because cohorts from different ancestries show systematic differences in allele frequencies. Similar to genotype PCs^37^, allele frequency PCs can also separate different ancestral groups. For example, the first allele frequency PC separates cohorts of individuals with recent African ancestries from those with other ancestries. As a rule of thumb, the number of PCs used could be determined by the number of relatively distinct ancestral groups of participating studies minus one, to yield sufficient degrees of freedom to separate different major ancestral groups. In our evaluations, we used three PCs, which is consistent with other applications of meta-regression models in multi-ancestry studies^36^. In our simulation study, we varied the number of PCs between two and four and the relative performance remained very similar.

TESLA is optimal when the phenotypic effects are mediated by the eQTL effects. When there are residual genetic effects of eQTL SNPs that influence phenotypes (for example, due to the LD between eQTL SNPs and other causal variants in the region), methods such as variance component (VC)-TWAS^38^ would be a useful complementary approach. VC-TWAS, in its original form, applies to individual-level data from a single study or summary association statistics. It does not accommodate multiple sources of input. A straightforward approach is to apply VC-TWAS to meta-analysis results. Given that VC-TWAS is an extension of the sequence kernel association test (SKAT)^39^, another possibility is to extend VC-TWAS in the same way as het-meta-SKAT^39^, which assumes that genetic effects are heterogeneous. These extensions may not be optimal, because they do not properly consider genetic effect heterogeneities across ancestries. It would be an important future research area to develop optimal strategies to integrate VC-TWAS into trans-ancestry genetic studies.

Although TESLA optimizes the power for TWASs using existing eQTL datasets, it does not take away the need to generate eQTL datasets from non-European populations. The ancestry of the eQTL dataset strongly influences the interpretation of TESLA results. When a European eQTL dataset is used, TESLA identifies target genes specific to European ancestry. Therefore, if a genetic variant has heterogeneous effects, meta-regression will put the most weight over cohorts of European ancestry and less weight on cohorts from non-European ancestry. Similarly, when an eQTL dataset of African ancestry is used (for example, nucleus accumbens from the LIBD dataset), TESLA identifies target genes in African ancestries and cohorts with individuals of African ancestries would contribute the most to meta-analysis. As additional non-European eQTL datasets are generated, TESLA will become even more useful to understand the impact of noncoding variants in non-European populations.

In summary, our study represents an attempt to extend GWASs and TWASs of tobacco use to non-European ancestries. The gene discoveries deepen our understanding of the etiology of tobacco use phenotypes and implicate translational applications. The methodology is broadly useful for next-generation trans-ancestry genetic studies of complex diseases and address critical challenges for multi-ancestry TWASs^40^. TESLA will further improve power over existing methods as more non-European GWASs and eQTL datasets are generated.

## Methods

In this section, we describe the smoking phenotype definition, the summary association statistics from the GSCAN and TOPMed consortium, as well as the TESLA method. The enrichment and drug-repurposing analyses are described in Supplementary Text. The detailed descriptions of transcriptomics datasets from the GTEx consortium, LIBD Human Brain Repository and mouse scRNA-seq data can also be found in Supplementary Text.

### Phenotype definition

We analyzed the following four smoking behavior-related traits because of their broad availability in existing epidemiological and medical studies, as well as their biological relevance to addiction behaviors:

1. Smoking initiation (SmkInit): a binary trait that compares ever smokers with never smokers. Ever smokers were defined as individuals who have smoked >99 cigarettes in their lifetime, which is consistent with the definition by the Center for Disease Control^44^.
2. Cigarettes per day (CigDay): a quantitative trait that measures the average number of cigarettes smoked per day by an ever smoker.
3. Smoking cessation (SmkCes): a binary trait that compares former against current smokers.
4. Age of smoking initiation (AgeInit): a continuous outcome that measures the age when one starts regular smoking.

More detailed definitions for the four phenotypes can be found in Supplementary Text, which is reproduced from our published GSCAN studies^1^.

### GWAS summary association statistics

Our study used GWAS summary association statistics from 61 participating studies as input (Supplementary Table 3). These studies were analyzed using either (generalized) linear models or linear mixed models and adjusted for age, sex and at least ten genetic PCs. The adjusted covariates may differ slightly between studies. All participating studies in the meta-analysis were examined by extensive quality control, including the check of Manhattan plots and quantile–quantile plots. The genomic control values for all participating cohorts are between 0.9 and 1.1 (Supplementary Table 15). We assessed the probability of the meta-analysis results being genuine using MAMBA^45^, a model-based method that relies on the strength of consistency of association signals across studies.

We use *b*_*jk*_ and *s*_*jk*_ to denote the phenotypic effects and standard deviation for variant *j* in study *k*. We further use $z_{jk} = b_{jk}/s_{jk}$ to denote the *z*-score statistic. In our analysis, standardized genotypes (that is, when genotypes are normalized to have mean 0 and variance of 1) are used, so that the standard deviation *s*_*jk*_ is inversely proportional to $\sqrt {n_{jk}}$, that is, $z_{jk} \approx \sqrt {n_{jk}} b_{jk}$. The results could be easily extended when nonstandardized genotypes were used. In sequence-based genetic studies, score statistics are often generated, from which we can derive approximate phenotypic effects using the above formula. The approximation is known to be accurate if true phenotypic effects are small^46^.

In addition to phenotypic effects and their standard deviations, we also take the PCs (or multi-dimensional scaling coefficients^36^) of the cohort allele frequencies as input, which serve as proxies for the cohort ancestry (Supplementary Fig. 4). Allele frequencies from different ancestry groups show systematic differences, which can be captured by the PCs.

### Proportionality condition for optimal TWAS power

We derived conditions for the TWAS statistic to have optimal power and used them to explain why direct integration of eQTL data with GWASs from different ancestries leads to suboptimal TWASs. We then proposed new and improved TWAS methods for integrating trans-ancestry GWASs with European eQTL datasets.

TWASs (and similar methods) were proposed to integrate eQTL effects with GWASs, to identify transcripts/genes that are associated with phenotypes. The TWAS statistic is often written in the form of a linear combination of *z*-score statistics (which is proportional to phenotypic effect estimates when standardized genotypes are used):

1 $$\begin{array}{*{20}{c}} {U_{{\mathrm{TWAS}}} = \mathop {\sum}\limits_j {w_j} z_j} \end{array}$$

where *w*_*j*_ are the weights obtained from a gene expression prediction model. The variance for the statistic $U_{{\mathrm{TWAS}}}$ equals:

2 $$\begin{array}{*{20}{c}} {V_{{\mathrm{TWAS}}} = {{{\mathbf{w}}}}^\prime {{V}}_{{z}}{\mathbf{w}}} \end{array}$$

where **w** is the vector of eQTL weights trained from gene expression prediction models, that is, ${{{\mathbf{w}}}} = \left( {w_1, \ldots ,w_J} \right)$, with *J* being the total number of variants used in the prediction model. *V*_*z*_ is the covariance matrix between *z*-score statistics, which can be approximated based on reference panels.

It is well understood that the choice of the weights can affect the power for the statistic $U_{{\mathrm{TWAS}}}$. To attain optimal power, the weights have to be chosen to maximize the noncentrality parameter of the test statistic, that is, $\mu _{{\mathrm{TWAS}}}^2 = \left( {E\left( {U_{{\mathrm{TWAS}}}/\sqrt {V_{{\mathrm{TWAS}}}} } \right)} \right)^2$. Applying Cauchy Schwarz inequality, a given set of eQTL weights yields the optimal power if they are proportional to the phenotypic effects, that is $w_j \propto \beta _j$. We call this the ‘proportionality condition’.

In TWAS methods, the eQTL effects are used as weights to combine phenotypic effect estimates of GWASs from the same ancestry. If the phenotypic effects are mediated by the eQTL effects, that is, $G_j\mathop { \to }\limits^{w_j} E\mathop { \to }\limits^c Y$, and the phenotypic and eQTL effects are homogeneous in samples from the same ancestry, the weights and phenotypic effects will satisfy the proportionality condition, that is, $\beta _j = w_jc$, and TWAS will yield optimal power as a gene-level test.

### Improved TWASs in trans-ancestry genetic studies

In contrast to TWASs using European GWASs and eQTL datasets, measured phenotypic effects can differ between ancestries in multi-ancestry genetic studies due to possibly different causal variants, allele frequencies or LD patterns. As a result, the proportionality condition may be violated when the GWAS and eQTL data come from different ancestries. Nor will the proportionality condition hold when FE or RE meta-analysis results from a multi-ancestry study are used with European eQTL dataset for TWASs. Suboptimal power is expected. Alternatively, if a TWAS is performed using European GWAS results and European eQTL dataset, and if the phenotypic effects and eQTL effects are homogeneous in the European population, the proportionality condition is expected to hold. Yet this strategy leaves out non-European GWAS data in the study and can still lead to suboptimal power when causal variants are shared between ancestries^47^.

Leveraging ancestral diversity while accounting for between-ancestry heterogeneities can improve the accuracy of the phenotypic effects in the matched ancestry of the eQTL data. For GWAS cohorts from different ancestries than the eQTL dataset, TESLA projects their phenotypic effects in the direction of eQTL weights, which are then meta-analyzed with other studies to get more accurate phenotypic effect estimates. TESLA uses these improved phenotypic effects to perform TWASs for optimal power.

### Multi-ancestry meta-regression models for phenotypic effects

We model the phenotypic effect estimates of eQTL SNPs of a given gene as a fixed effect of the ancestry captured by the allele frequency PCs. To calculate the PCs of allele frequencies, we code the allele frequency matrix using variant sites shared across all studies as *F*, where each row represents a study and each column represents a variant site. We then perform singular value decomposition for *F*, that is, $F = C_FD_FE_F^\prime$. In our analyses, we use the first three PCs, which is the first three columns of the matrix $FE_F$. We denoted the *l*th PCs for study *k* as $X_{kl}$ and the phenotypic effects of multiple genetic variants in study *k* as $b_{ \cdot k}$. For notational convenience, we fix $X_{k0}$ to 1.

We vary the number of PCs used (that is, *L*) and consider a series of models $M^{[L]}$:

3 $$\begin{array}{*{20}{c}} {M^{\left[ L \right]}:b_{ \cdot k} = \mathop {\sum}\limits_{l = 0}^L {X_{kl}} \gamma _{l \cdot }^{\left[ L \right]} + {\it{\epsilon }}_{ \cdot k}} \end{array}$$

where $b_{ \cdot k} = (b_{1k}, \ldots ,b_{Jk})$ is the phenotypic effects of eQTL SNPs $1, \ldots ,J$ for the gene and ${\it{\epsilon }}_{ \cdot k} = \left( {{\it{\epsilon }}_{1k}, \ldots ,{\it{\epsilon }}_{Jk}} \right)$ is the vector of residuals. The residuals follow multivariate normal distribution. $\gamma _{l \cdot }^{[L]} = \left( {\gamma _{l1}^{[L]}, \ldots ,\gamma _{lJ}^{[L]}} \right)$ are the regression coefficients for variants $1, \ldots ,J$.

In our simulations and data analyses, we considered *L* = 0, 1, 2 or 3. When no PCs are included in the model, it is equivalent to the FE meta-analysis, which is suitable for modeling variants that have homogeneous effects across studies. When one or more PCs are included in the model, it can capture phenotypic effect heterogeneity between studies.

Under model *M*^[L]^, the phenotypic effect follows a normal distribution:

$$b_{jk}|M^{\left[ L \right]}\sim N\left( {\mathop {\sum }\limits_{l = 1}^L X_{kl}\gamma _{lj}^{\left[ L \right]},s_{jk}^2} \right).$$

Model *M*^[*L*]^ can be fitted using the weighted least square method^36^. The solution satisfies:

4 $$\begin{array}{*{20}{c}} {{{{\hat{\boldsymbol \gamma }}}}_{ \cdot {{{\boldsymbol{j}}}}}^{\left[ {{{\boldsymbol{L}}}} \right]} = \left( {{{{\boldsymbol{X}}}}^{\left[ {{{\boldsymbol{L}}}} \right]^\prime }{{{\mathbf{\Omega }}}}_{{{\boldsymbol{j}}}}{{{\boldsymbol{X}}}}^{\left[ {{{\boldsymbol{L}}}} \right]}} \right)^{ - 1}{{{\boldsymbol{X}}}}^{\left[ {{{\boldsymbol{L}}}} \right]^\prime }{{{\mathbf{\Omega }}}}_{{{\boldsymbol{j}}}}{{{\boldsymbol{b}}}}_{{{{\boldsymbol{j}}}} \cdot }} \end{array}$$

where $${{{\mathbf{\Omega }}}}_{{{\boldsymbol{j}}}} = {\mathrm{diag}}\left( {s_{j1}, \ldots ,s_{jK}} \right)$$.

Based on meta-regression coefficients, we can estimate phenotypic effects in the ancestry of the eQTL dataset so that the eQTL weights and phenotypic effect estimates satisfy the proportionality condition. The first *L* PC coordinates of the eQTL dataset are denoted $\tilde X^{[L]}$ and the phenotypic effect estimates in the ancestry of the eQTL dataset are given by:

$${\hat {b_j}}^{[L]} = {{{\tilde{\boldsymbol X}}}}^{[{{{\boldsymbol{L}}}}]}{{{\hat{\boldsymbol \gamma }}}}_{{{\boldsymbol{j}}}}^{[{{{\boldsymbol{L}}}}]} = {{{\tilde{\boldsymbol X}}}}^{\left[ {{{\boldsymbol{L}}}} \right]}\left( {{{{\boldsymbol{X}}}}^{\left[ {{{\boldsymbol{L}}}} \right]^\prime }{{{\mathbf{\Omega }}}}_{{{\boldsymbol{j}}}}{{{\boldsymbol{X}}}}^{\left[ {{{\boldsymbol{L}}}} \right]}} \right)^{ - 1}{{{\boldsymbol{X}}}}^{\left[ {{{\boldsymbol{L}}}} \right]^\prime }{{{\mathbf{\Omega }}}}_{{{\boldsymbol{j}}}}{{{\boldsymbol{b}}}}_{{{{\boldsymbol{j}}}} \cdot }$$

We denote the vector of estimated effects as ${{{\hat{\mathbf{b}}}}}^{[L]} = \left( {{\hat {b_1}}^{\left[ L \right]}, \ldots ,{\hat {b}}_J^{\left[ L \right]}} \right)$, the covariance matrix of which is ${{{\mathbf{\Sigma }}}}_{{{\mathbf{b}}}}^{[{{{\boldsymbol{L}}}}]}$. To calculate ${{{\mathbf{\Sigma }}}}_{{{\mathbf{b}}}}^{[{{{\boldsymbol{L}}}}]}$, we use the fact that the predicted phenotypic effects ${\hat {b_j}}^{[L]}$ are a linear combination of the phenotypic effects across all participating studies. As a result, we can calculate the correlation between the predicted effects of variants *j*_1_ and *j*_2_, that is, $b_{j_1}^{\left[ L \right]}$ and $b_{j_2}^{\left[ L \right]}$, based on the correlations between $b_{j_1k}$ and $b_{j_2k}$ in each study *k*. Given that each cohort may come from different ancestries, we use ancestry-specific reference panels to estimate LD and approximate the correlations between $b_{j_1k}$ and $b_{j_2k}$. Detailed derivation of the covariance matrix can be found in Supplementary Text. The standard deviation for the estimated effects ${{{\hat{\mathbf b}}}}^{[L]}$ is denoted by ${{{\hat{\mathbf {s}}}}}^{[{{{\boldsymbol{L}}}}]} = \left( {{\hat {s_1}}^{\left[ L \right]}, \ldots ,{\hat {s_J}}^{\left[ L \right]}} \right)$, which equals the square root of the diagonal entries of ${{{\mathbf{\Sigma }}}}_{{{\mathbf{b}}}}^{[{{{\mathbf{L}}}}]}$.

### TESLA using predicted phenotypic effect

Based on the phenotypic effect estimate ${{{\hat{\mathbf b}}}}^{[L]}$ and its standard deviation ${{{\hat{\mathbf s}}}}^{[L]}$, we constructed our TWAS statistic as $U_{{\mathrm{TWAS}}}^{[L]} = \mathop {\sum }\limits_{j = 1}^J w_j{\hat {b_j}}^{[L]}/{\hat {s_j}}^{[L]}$. The variance for the statistic equals:

5 $$\begin{array}{*{20}{c}} {V_{{\mathrm{TWAS}}}^{\left[ L \right]} = {{{\mathbf{w}}}}^\prime \left( {{{{\mathrm{diag}}}}\left( {{\hat {s_1}}^{\left[ L \right]}, \ldots ,{\hat {s_J}}^{\left[ L \right]}} \right)} \right)^{ - 1}{{{\mathbf{\Sigma }}}}_{{{\mathbf{b}}}}^{[{{{\boldsymbol{L}}}}]}\left( {{{{\mathrm{diag}}}}\left( {{\hat {s_1}}^{\left[ L \right]}, \ldots ,{\hat {s_J}}^{[L]}} \right)} \right)^{ - 1}{\mathbf{w}}} \end{array}$$

We further calculated the standardized statistic as $T_{{\mathrm{TWAS}}}^{[L]} = U_{{\mathrm{TWAS}}}^{\left[ L \right]}/\sqrt {V_{{\mathrm{TWAS}}}^{[L]}}$.

Four different TWAS statistics are calculated that correspond to the models with 0–3 PCs. The model with 0 PC is equivalent to FE-TWAS. When the same eQTL weights are used in each study, FE-TWAS is also equivalent to conducting TWAS in each participating study and then combining results using inverse-variance, weighted meta-analysis (Supplementary Text).

We use a minimal *P*-value approach to find the overall *P* value for the statistic. Specifically, we denote the *P* values for the four statistics as $P^{[0]}$, …, $P^{[3]}$. The minimal *P*-value statistic $P^ \ast = \min \left( {P^{\left[ 0 \right]}, \ldots ,P^{\left[ 3 \right]}} \right)$ follows:

6 $$\begin{array}{*{20}{l}} \Pr \left( {P^ \ast < p^ \ast } \right) = 1 - \Pr \left( P^ \ast > p^ \ast \right) = 1 - \Pr \left( {{\Phi }}^{ - 1}\left( {1 - p^ \ast } \right) < T_{{\mathrm{TWAS}}}^{[1]}\right. \\\left.< {{\Phi }}^{ - 1}\left( {p^ \ast } \right), \ldots ,{{\Phi }}^{ - 1}\left( {1 - p^ \ast } \right) < T_{{\mathrm{TWAS}}}^{[4]} < {{\Phi }}^{ - 1}\left( {p^ \ast } \right) \right) \end{array}$$

which can be evaluated using multivariate normal distribution function. Details can be found in Supplementary Text.

### Multi-tissue TESLA statistic using the Cauchy combination

In addition to the single-tissue TESLA statistic, we also calculated a cross-tissue TWAS statistic. Numerous methods exist to combine *P* values from correlated test statistics, from which we chose to use the Cauchy combination^14^ due to its excellent power and the ease of calculation. In our analysis, we assigned equal weight to each tissue in the Cauchy combination test.

### Reporting summary

Further information on research design is available in the Nature Portfolio Reporting Summary linked to this article.

## Online content

Any methods, additional references, Nature Portfolio reporting summaries, source data, extended data, supplementary information, acknowledgements, peer review information; details of author contributions and competing interests; and statements of data and code availability are available at 10.1038/s41588-022-01282-x.

## Supplementary information

Supplementary Information Supplementary Figs. 1–9, Tables 1–9 and legends for Supplementary Tables 10–15.

Reporting Summary

Supplementary Table 1 Supplementary Tables 5 and 10–15.

## Data Availability

We implemented a Shiny app for users to interactively explore research results, which is available at https://liugroupstatgen.shinyapps.io/shiny-tesla-only. Precomputed gene expression prediction model weights of 48 tissues are from the PrediXcan website (GTEx v.7): https://predictdb.org. GO and pathway gene sets are from MSigDB (https://www.gsea-msigdb.org/gsea/msigdb). RNA-seq and genotype data from postmortem nucleus accumbens samples of physiologically normal human brains are from the LIBD Human Brain Repository Data (http://eqtl.brainseq.org/phase2/eqtl).

## Code availability

TELSA is implemented in our rare GWAMA software package and made available at GitHub (https://github.com/funfunchen/rareGWAMA) and *Zenodo* (10.5281/zenodo.7352120)^48^. Other software used includes MAGMA (v.1.08; https://ctg.cncr.nl/software/magma); REVIGO (accessed May 2022; http://revigo.irb.hr); METASOFT (v.2.0.1; http://zarlab.cs.ucla.edu/software); MAMBA (v.1.12; https://github.com/dan11mcguire/mamba); MetaXcan (v.0.7.1; https://github.com/hakyimlab/MetaXcan); R Shiny (v.1.7.2; https://cran.r-project.org/web/packages/shiny/index.html); and ggseg (v.1.5.3; https://github.com/ggseg/ggseg).
