# Supplementary material for: Multi-ancestry transcriptome-wide association analyses yield insights into tobacco use biology and drug repurposing
Source: Nat Genet. 2023 Jan 26;55(2):291–300. doi: 10.1038/s41588-022-01282-x (PMC9925385; doi:10.1038/s41588-022-01282-x)
Supplement: Supplementary file 1 — Supplementary Figs. 1–9, Tables 1–9 and legends for Supplementary Tables 10–15. [file 41588_2022_1282_MOESM1_ESM.pdf]

nature genetics

Article https://doi.org/10.1038/s41588-022-01282-x

Multi-ancestry transcriptome-wide association analyses yield insights into tobacco use biology and drug repurposing

In the format provided by the
authors and unedited

Supplementary Figures

Supplementary Figure 1: Distribution of genomic control values. **Panels a-d** showed violin plots of the genomic control values of four TWAS methods using GTEx eQTL data (i.e., TESLA, FE-TWAS, RE-TWAS and EURO-TWAS) for four smoking traits, i.e., a) AgeInit, b) CigDay, c) SmkInit and d) SmkCes. Each data point in the violin plot represents the genomic control value from a tissue. The line in the middle of each box represents median, the bold red dot in the middle of the box represents the mean. The upper and lower bounds of the box represent the 25th and 75th percentile, and whiskers are 1.5 times the inter-quartile range. The contours of the "violin" represents the density function of the data points. We plot any outliers that are outside the range of 1.5 times IQR. All methods have well-behaved genomic control values across all scenarios. Panels e-l displayed the Quantile-Quantile plot of p-values for four TWAS methods using LIBD eQTL dataset from nucleus accumbens in African and European ancestries. Panels e and f are the results for trait AgeInit for EUR-TWAS and AFR-TWAS respectively. Panels g and h are the results for trait CigDay for EUR-TWAS and AFR-TWAS respectively. Panels i and j are the results for trait SmkCes for EUR-TWAS and AFR-TWAS respectively. Panels k and l are the results for trait SmkInit for EUR-TWAS and AFR-TWAS respectively. Shaded areas in the plot represent the 95% confidence band of different quantiles of $-\log_{10}(\mathrm{p}-\mathrm{value})$.

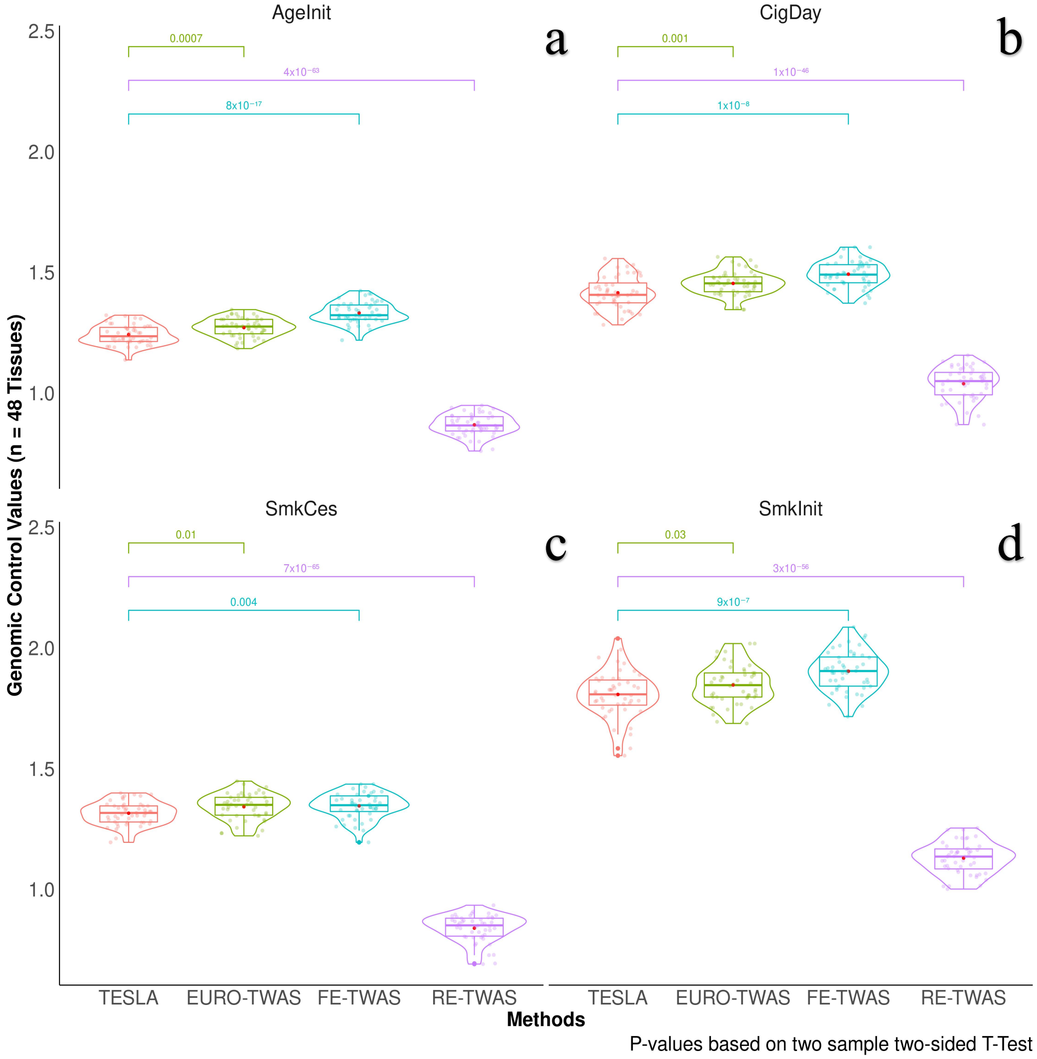

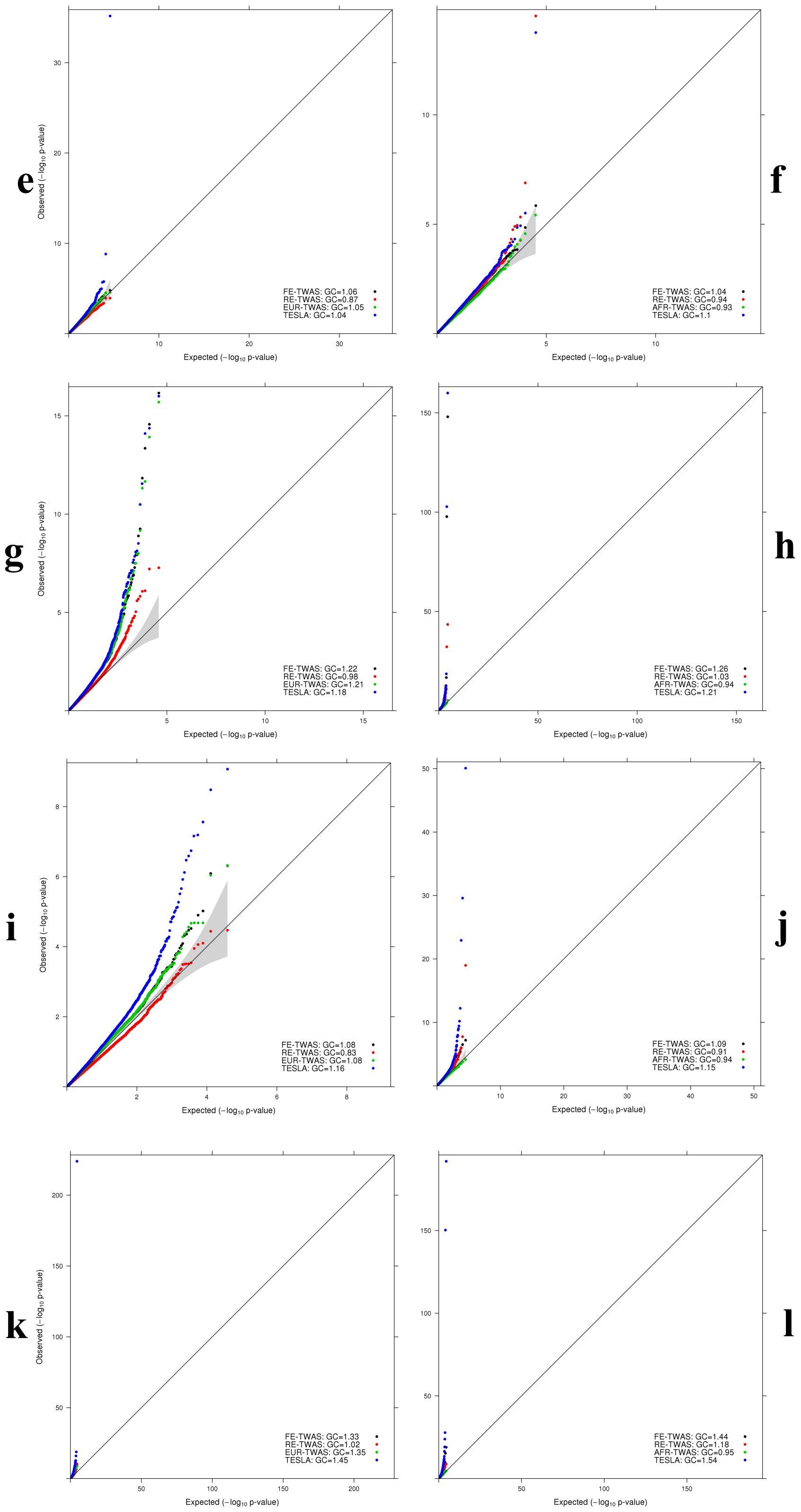
e
f
g
h
i
j
k
l
Observed (−log10 p-value)
Expected (−log10 p-value)
FE-TWAS: GC=1.06
RE-TWAS: GC=0.87
EUR-TWAS: GC=1.05
TESLA: GC=1.04
FE-TWAS: GC=1.04
RE-TWAS: GC=0.94
AFR-TWAS: GC=0.93
TESLA: GC=1.1
FE-TWAS: GC=1.22
RE-TWAS: GC=0.98
EUR-TWAS: GC=1.21
TESLA: GC=1.18
FE-TWAS: GC=1.26
RE-TWAS: GC=1.03
AFR-TWAS: GC=0.94
TESLA: GC=1.21
FE-TWAS: GC=1.08
RE-TWAS: GC=0.83
EUR-TWAS: GC=1.08
TESLA: GC=1.16
FE-TWAS: GC=1.09
RE-TWAS: GC=0.91
AFR-TWAS: GC=0.94
TESLA: GC=1.15
FE-TWAS: GC=1.33
RE-TWAS: GC=1.02
EUR-TWAS: GC=1.35
TESLA: GC=1.45
FE-TWAS: GC=1.44
RE-TWAS: GC=1.18
AFR-TWAS: GC=0.95
TESLA: GC=1.54

Supplementary Figure 2: Manhattan plot for TESLA p-values. For panels a-c, we labeled the fine mapped genes with posterior inclusion probability (PIP) > 0.9 in the Manhattan plot with two-sided p-value < 2.5×10^{-6} from multi-tissue TESLA analysis against their chromosomal positions (Manhattan CigDay is shown in Figure 2). For smoking initiation trait, there are a large number of fine mapped signals, so we only labelled 10 genes per chromosome with the largest PIP values. Panel d-g show the $-\log_{10}$ (two-sided p-values) for AgeInit, SmkInit, SmkCes and CigDay with European LIBD eQTL data from nucleus accumbens. Panel h-k show the $-\log_{10}$ (two-sided p-values) for AgeInit, SmkInit, SmkCes and CigDay with African LIBD eQTL data from nucleus accumbens. For each chromosome, all significant genes were labeled (two-sided p-value < 2.5×10^{6}).

(a) Top10 significant fine mapped genes on each chromosome for SmkInit multi-tissue TESLA results using GTEx

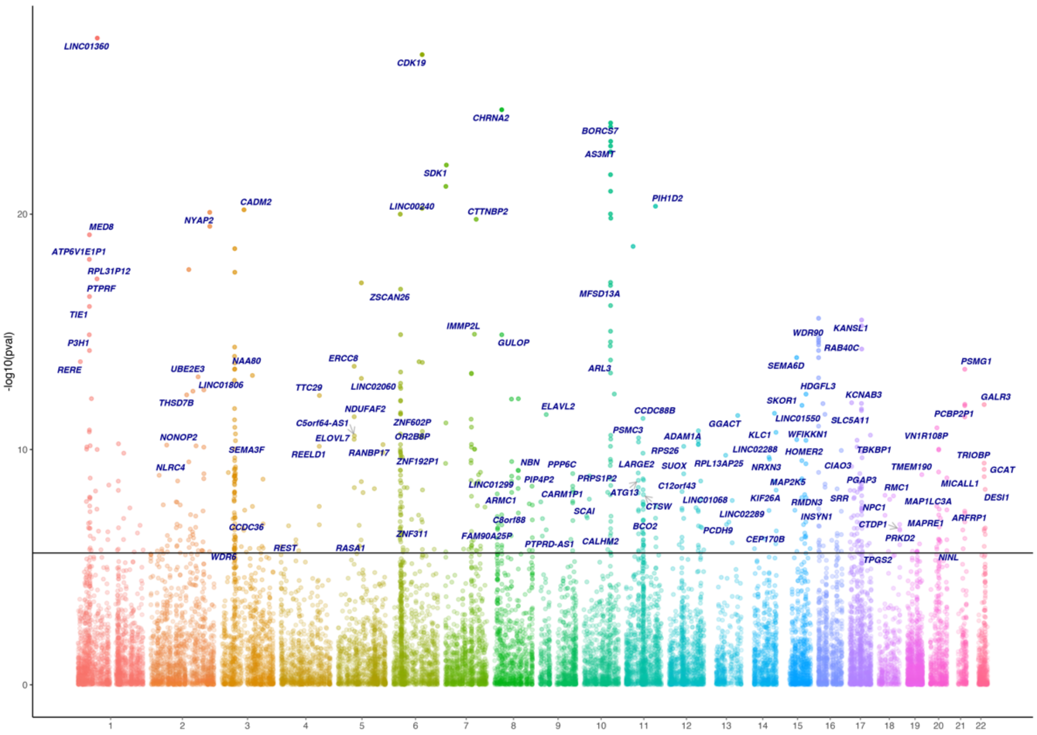

(b) Fine mapped genes with PIP > .90 on each chromosome for AgeInit multi-tissue TESLA results using GTEx.

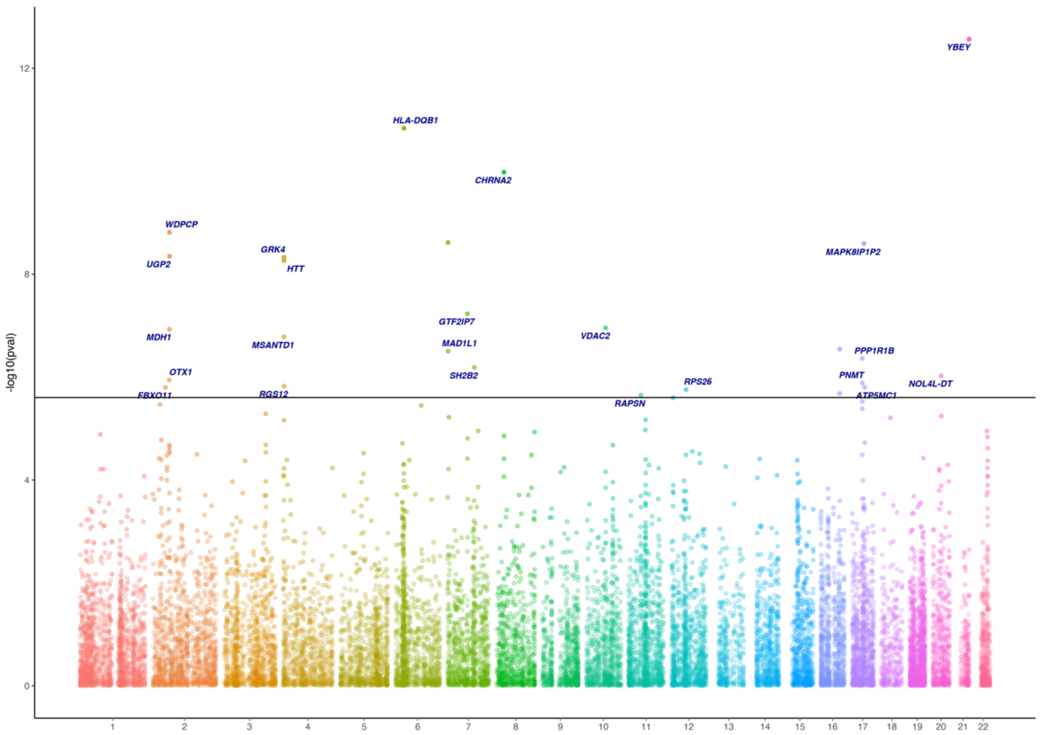

(c) Fine mapped genes with PIP > .90 on each chromosome for SmkCes multi-tissue TESLA results using GTEx.

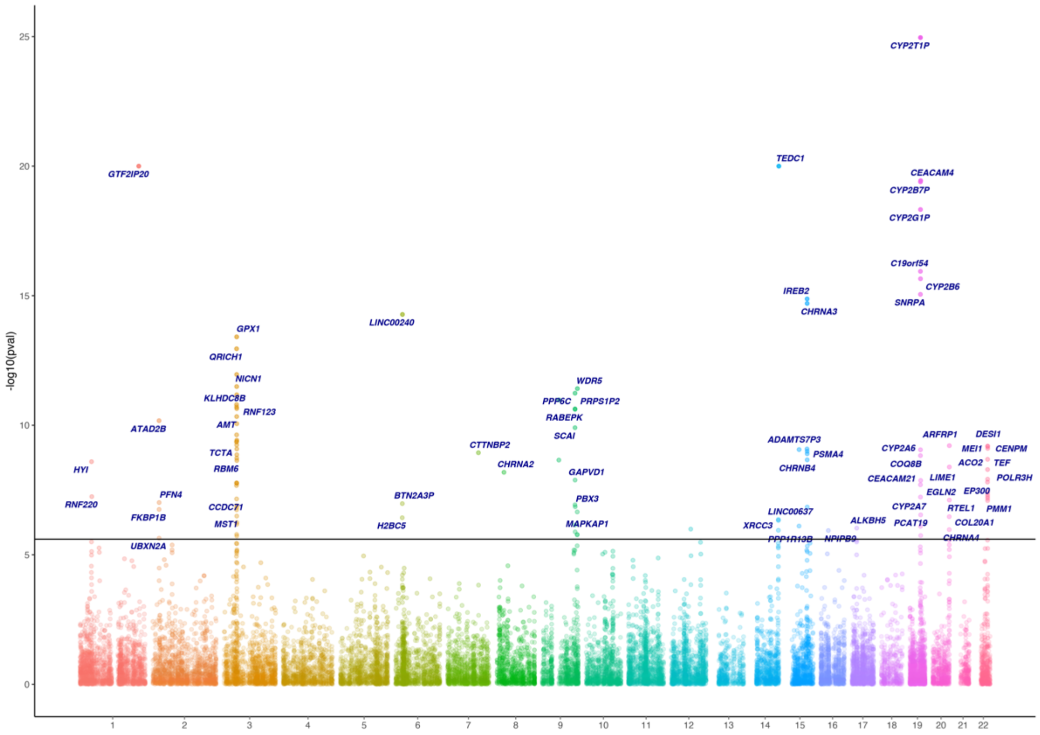

(d) Fine mapped genes with PIP > .90 on each chromosome for AgeInit TESLA results using LIBD European eQTL dataset.

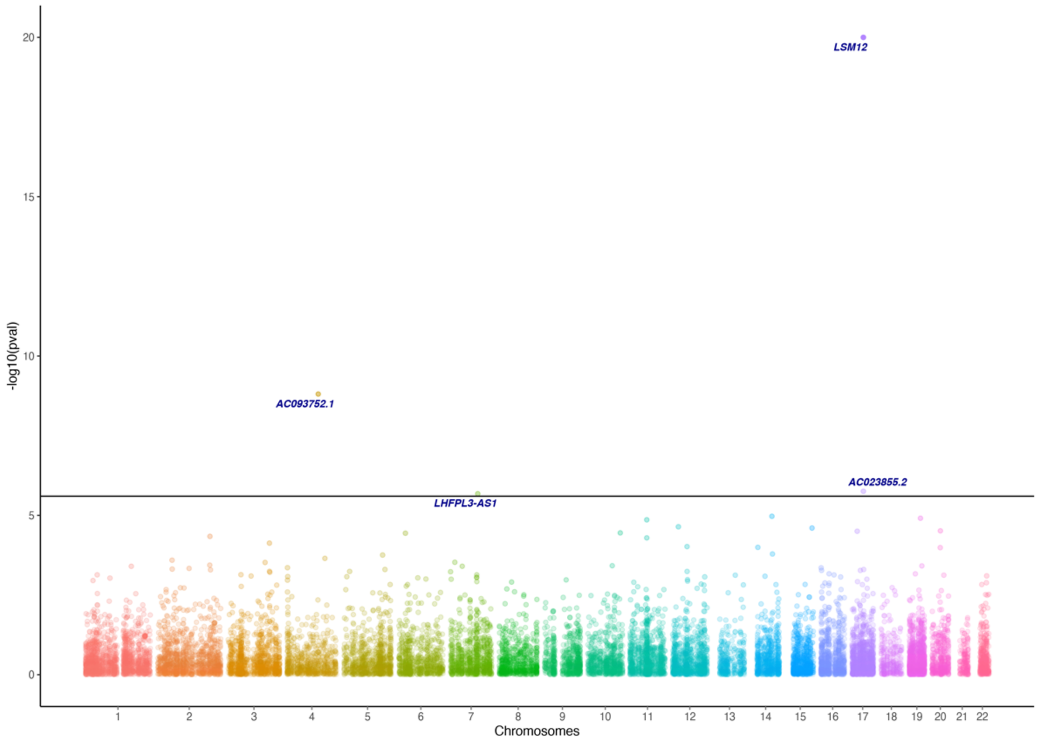

(e) Fine mapped genes with PIP > .90 on each chromosome for SmkInit TESLA results using LIBD European eQTL dataset.

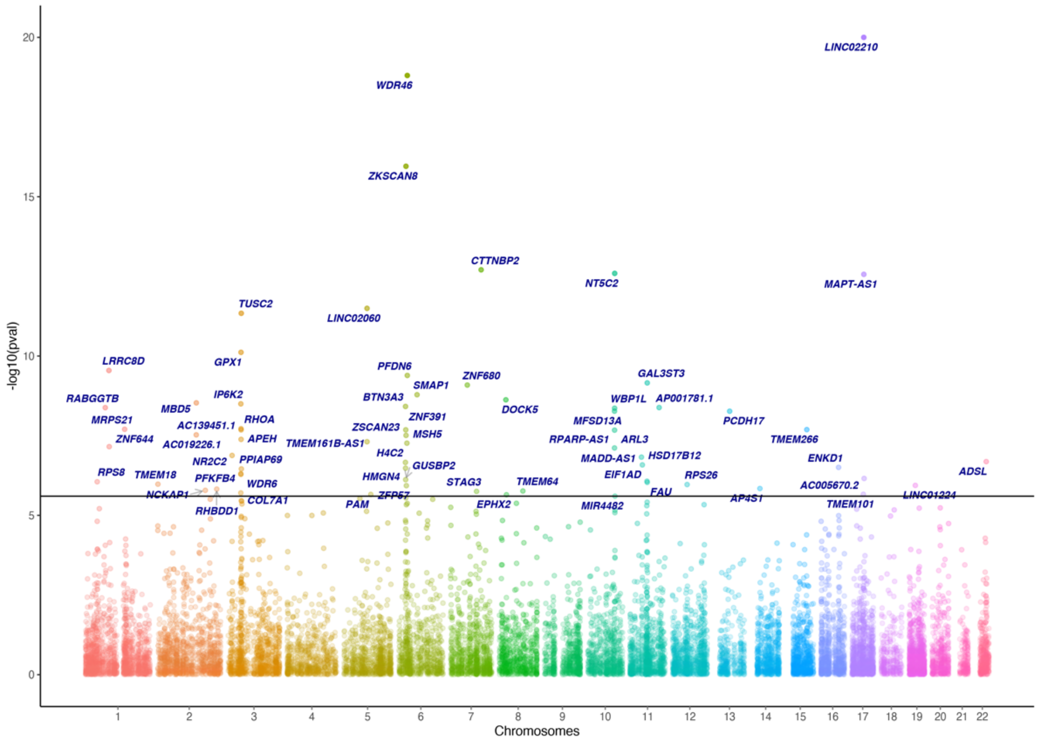

(f) Fine mapped genes with PIP > .90 on each chromosome for SmkCes TESLA results using LIBD European eQTL dataset.

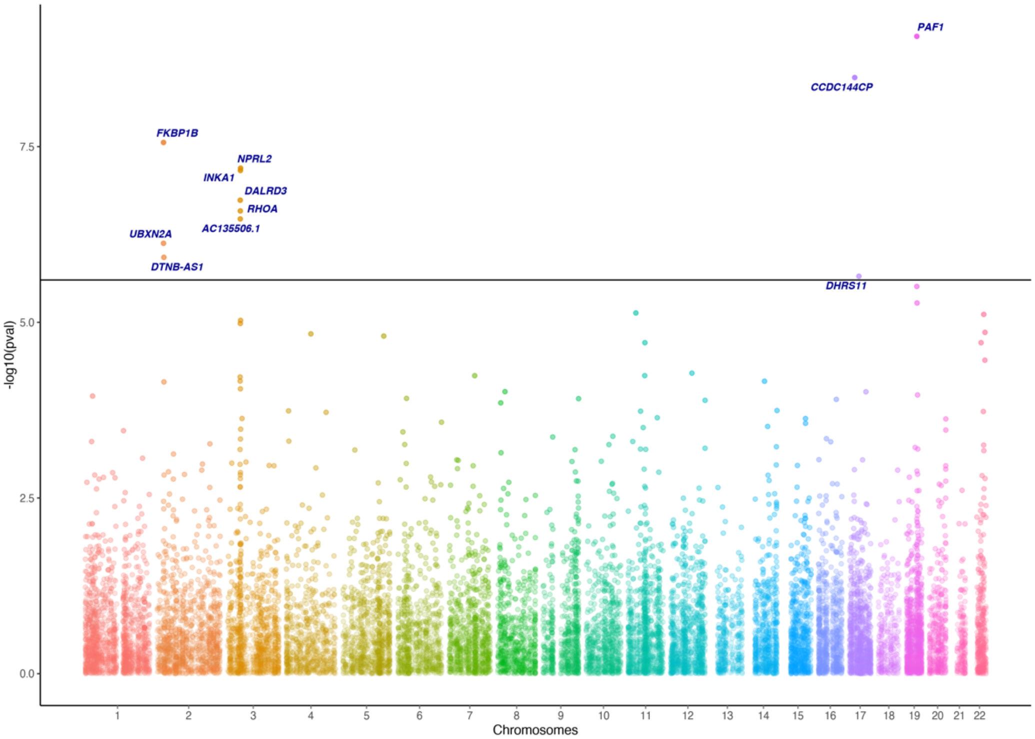

(g) Fine mapped genes with PIP > .90 on each chromosome for CigDay TESLA results using LIBD European eQTL dataset.

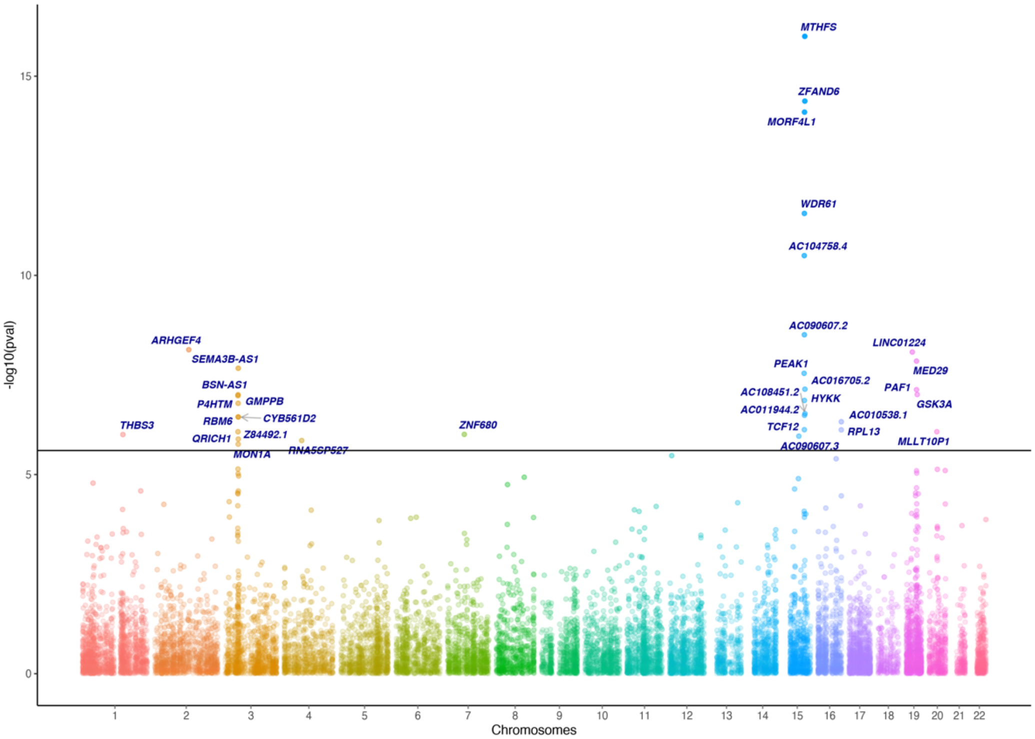

(h) Fine mapped genes with PIP > .90 on each chromosome for AgeInit TESLA results using LIBD African eQTL dataset.

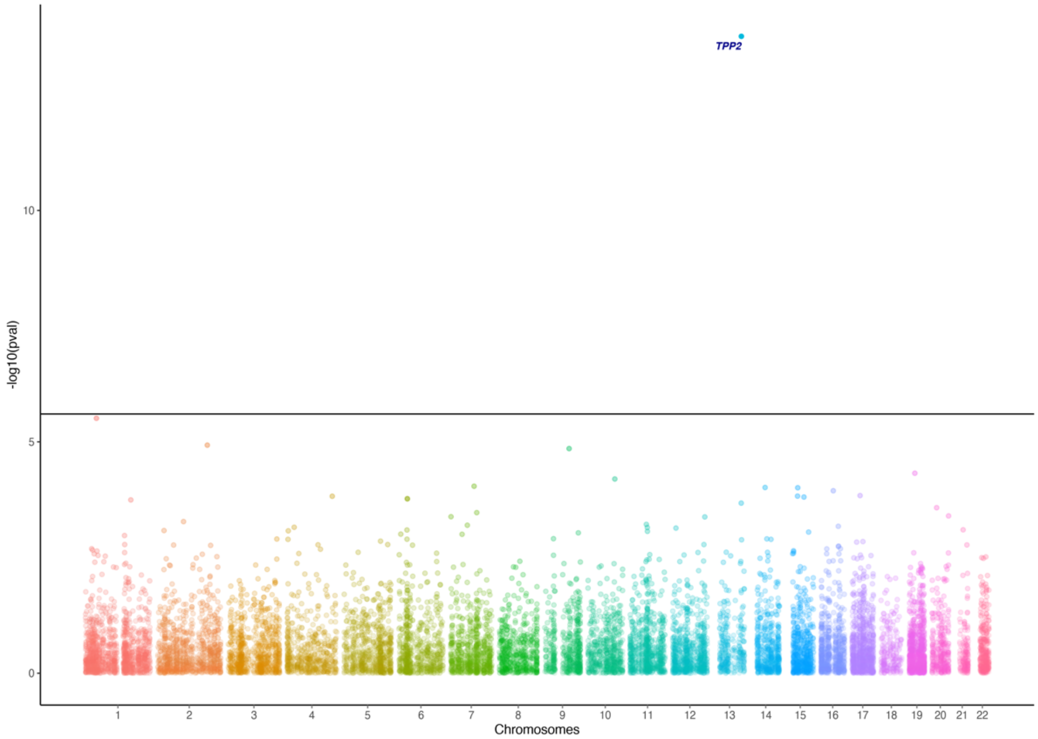

(i) Fine mapped genes with PIP > .90 on each chromosome for SmkInit TESLA results using LIBD African eQTL dataset.

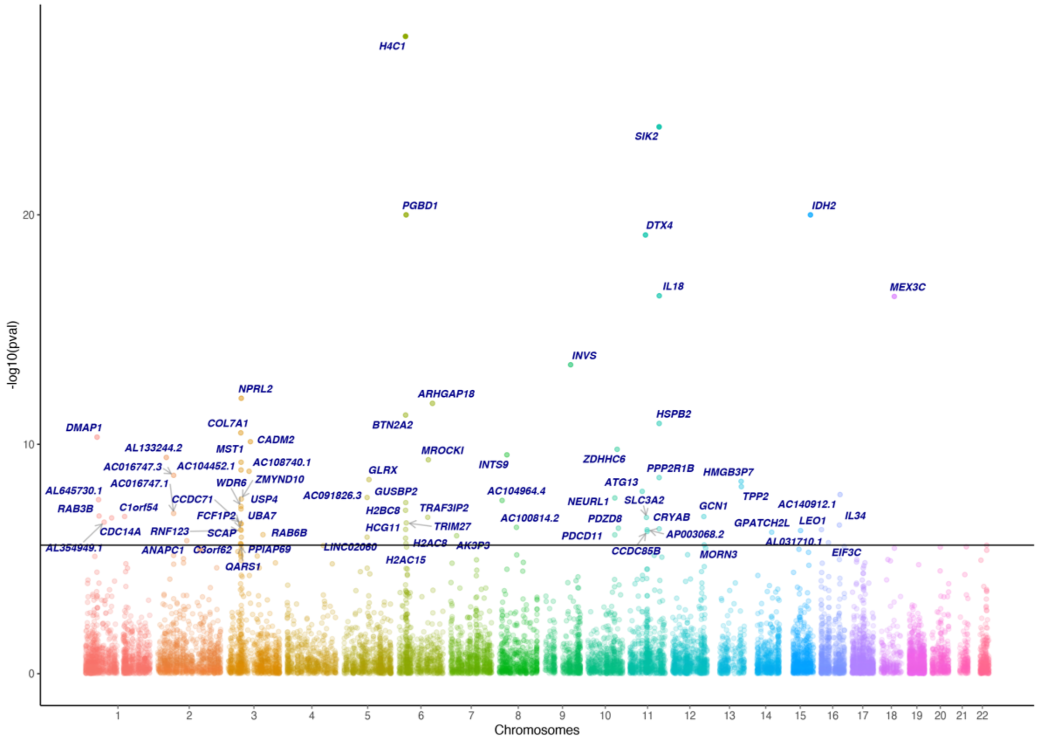

(j) Fine mapped genes with PIP > .90 on each chromosome for SmkCes TESLA results using LIBD African eQTL dataset.

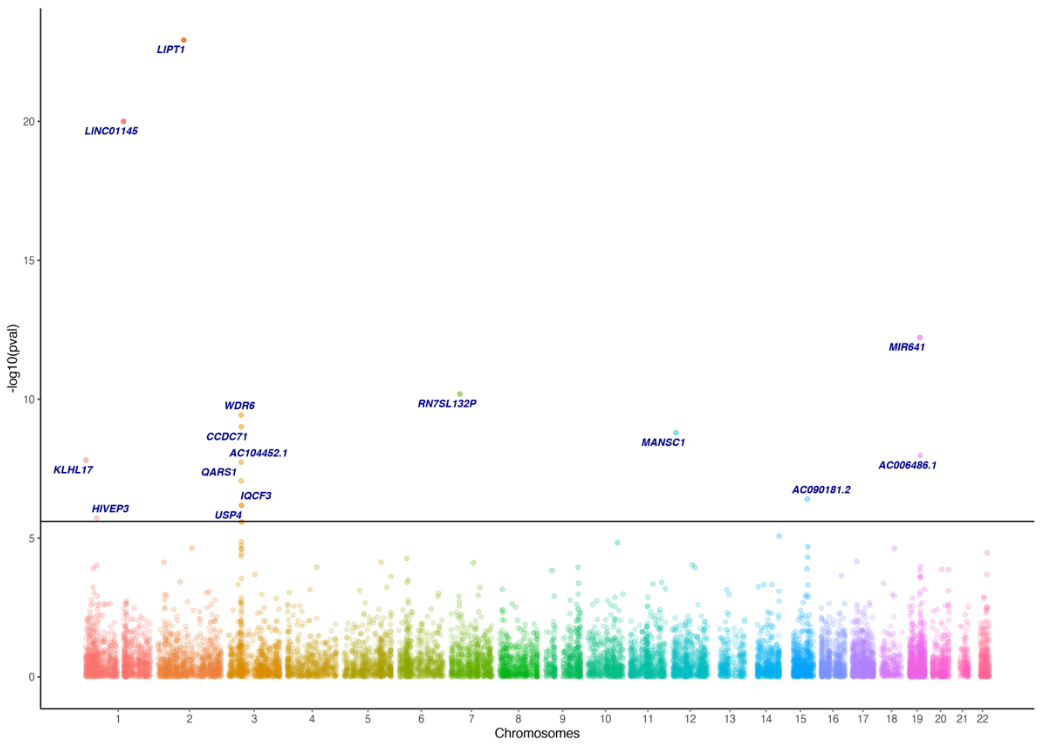

(k) Fine mapped genes with PIP > .90 on each chromosome for CigDay TESLA results using LIBD African eQTL dataset.

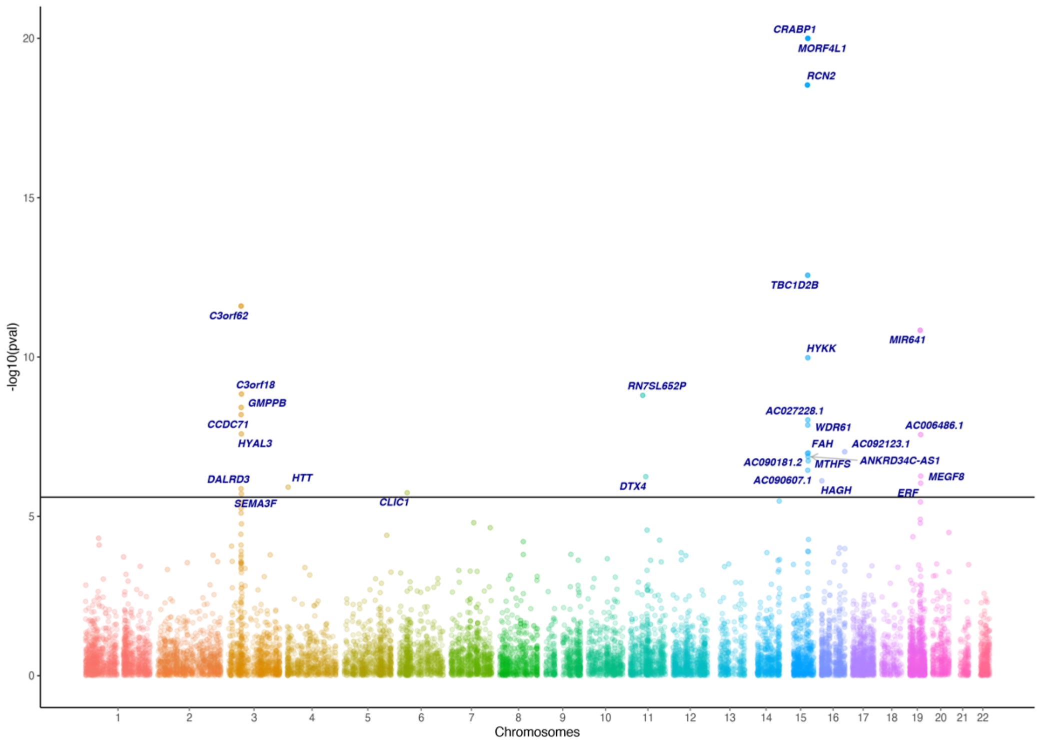

Supplementary Figure 3: The number of significant genes identified by TESLA. The number of statistically significant gene associations (two-sided p-value $< 2.5 \times 10^{-6}$) identified in different tissues for four smoking phenotypes are shown in the lower panel. The intensity of the color reflects the number of loci. In the last column, we showed the number of significant gene associations using multi-tissue TESLA method. Results from brain tissues were highlighted in a blue box. The sample sizes for GTEx tissues are shown in the upper panel.

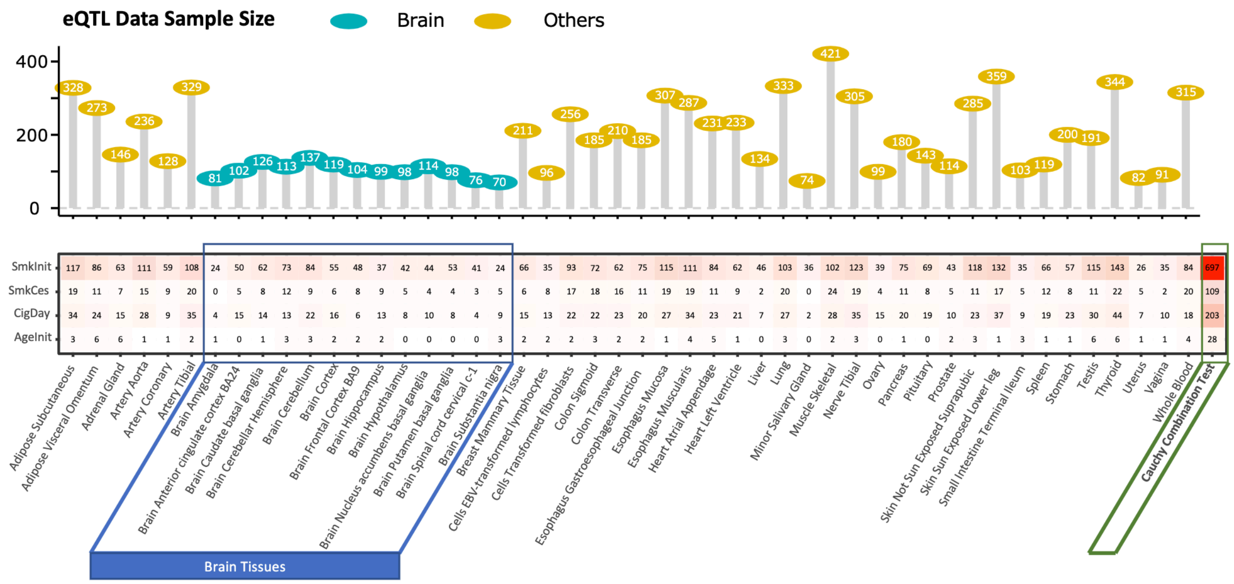

Supplementary Figure 4: **Principal component map of genome-wide allele frequencies from participating cohorts.** We calculated principal components from genome-wide allele frequencies of variant sites that are commonly measured in all studies. Allele frequencies from 1000 Genome Project ancestry groups were also included. Ancestries of the participating studies were classified based on the closest 1000 Genome Project ancestry group using Euclidean distance.

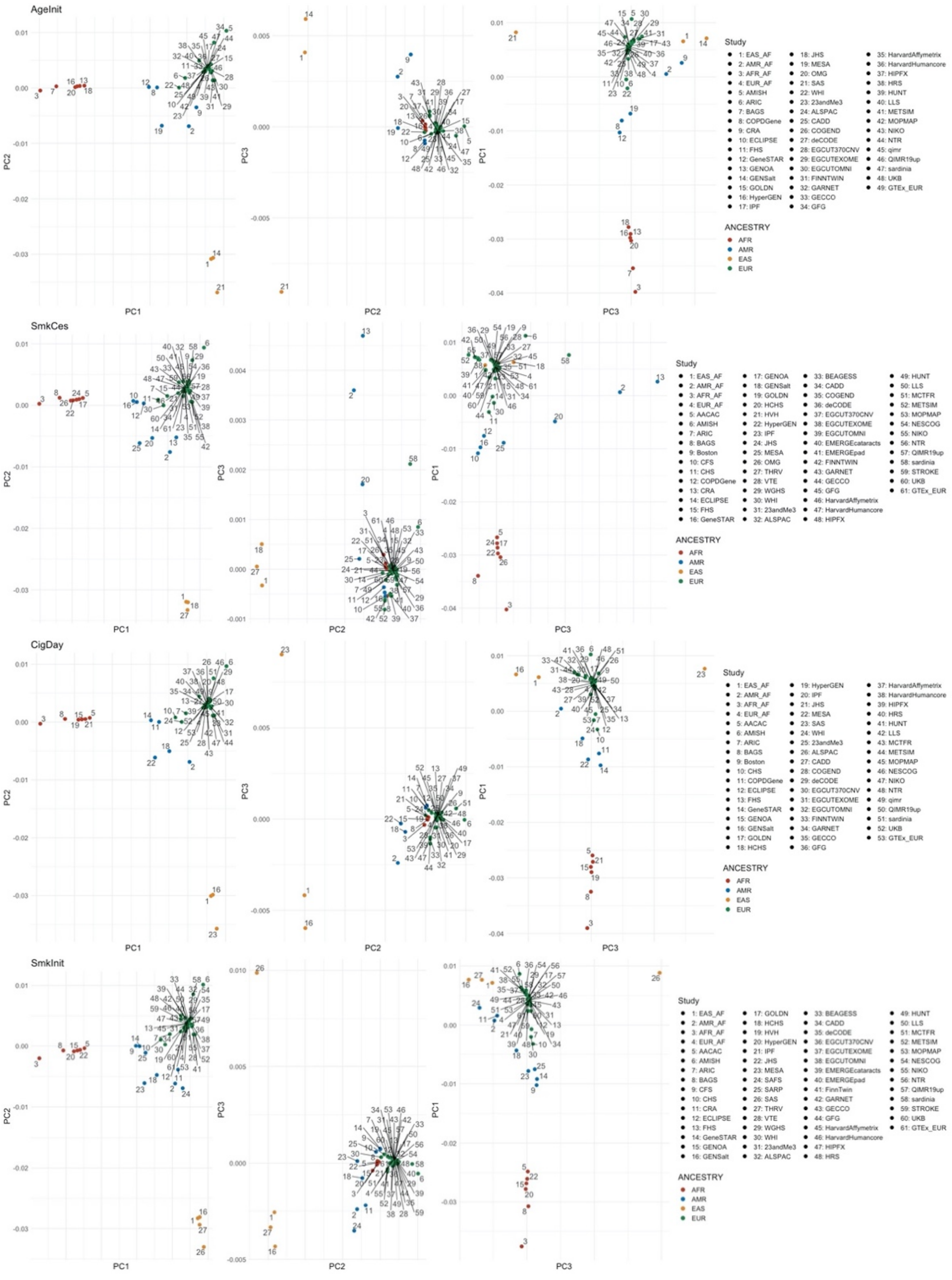

Supplementary Figure 5: Meta-regression models that generate minimal p-values inform the extent of heterogeneity in phenotypic effect sizes. TESLA fits multiple meta-regression models with varying number of allele frequency principal components. Based on estimated phenotypic effects in each meta-regression model, we perform TWAS and combine the results using minimal p-values (two-sided). The meta-regression model that yields the minimal p-value will inform the extent of phenotypic heterogeneity between ancestries. For example, the meta-regression model with 0 PC included is equivalent to fixed effect meta-analysis. The first PC separates African and non-African ancestry. Meta-regression model with 1 PC is likely to give the minimal p-value for genes that show different effects in African American ancestry. Here, among genes that reach significance level $\alpha = 2.5 \times 10^{-6}$, we report the fraction and number of different meta-regression models that produce minimal p-values for each tissue. The distribution of different models from all tissues were summarized in Supplementary Table 6. Panels a-d represent results for SmkCes, SmkInit, CigDay and AgeInit respectively.

(a)

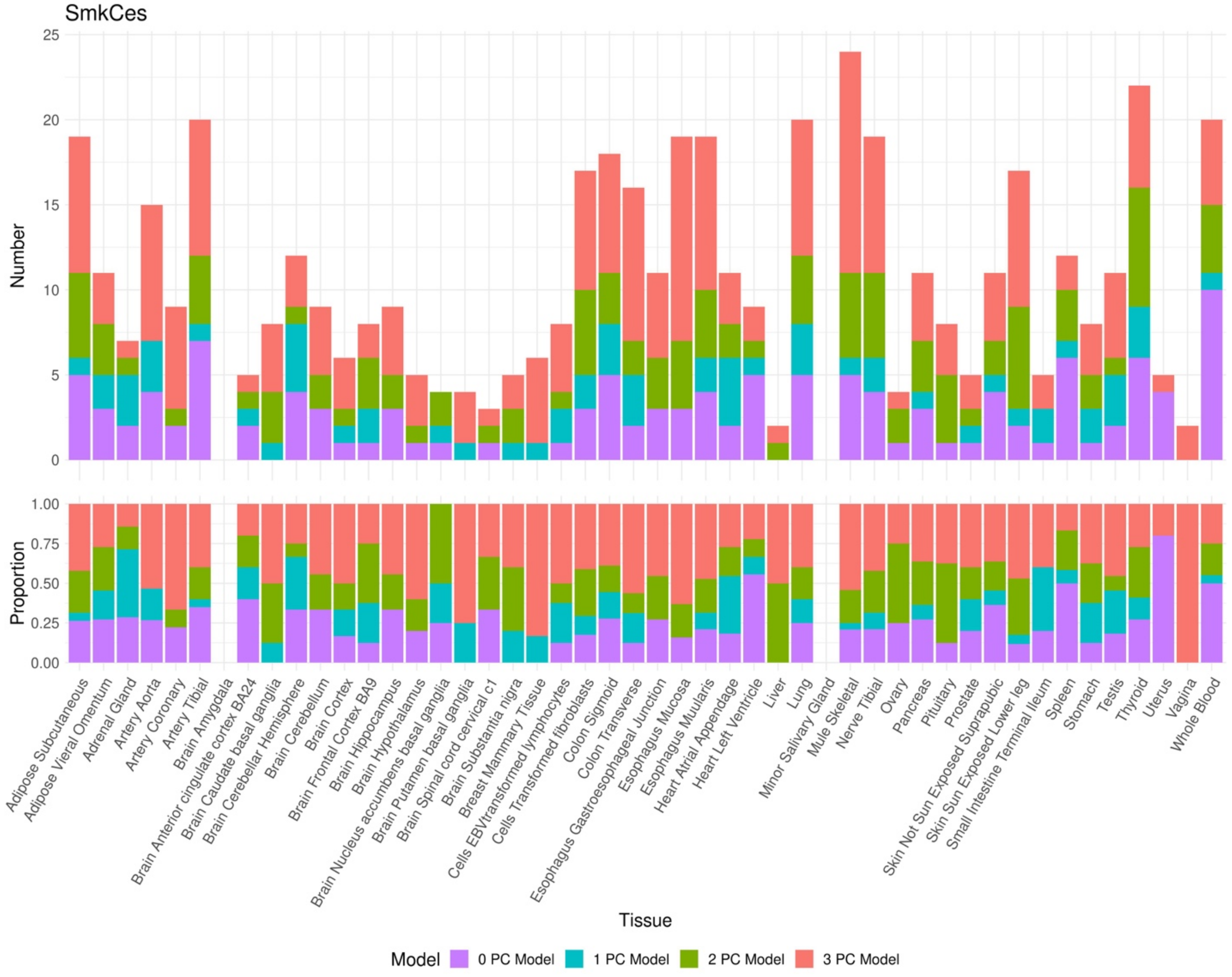

(b)

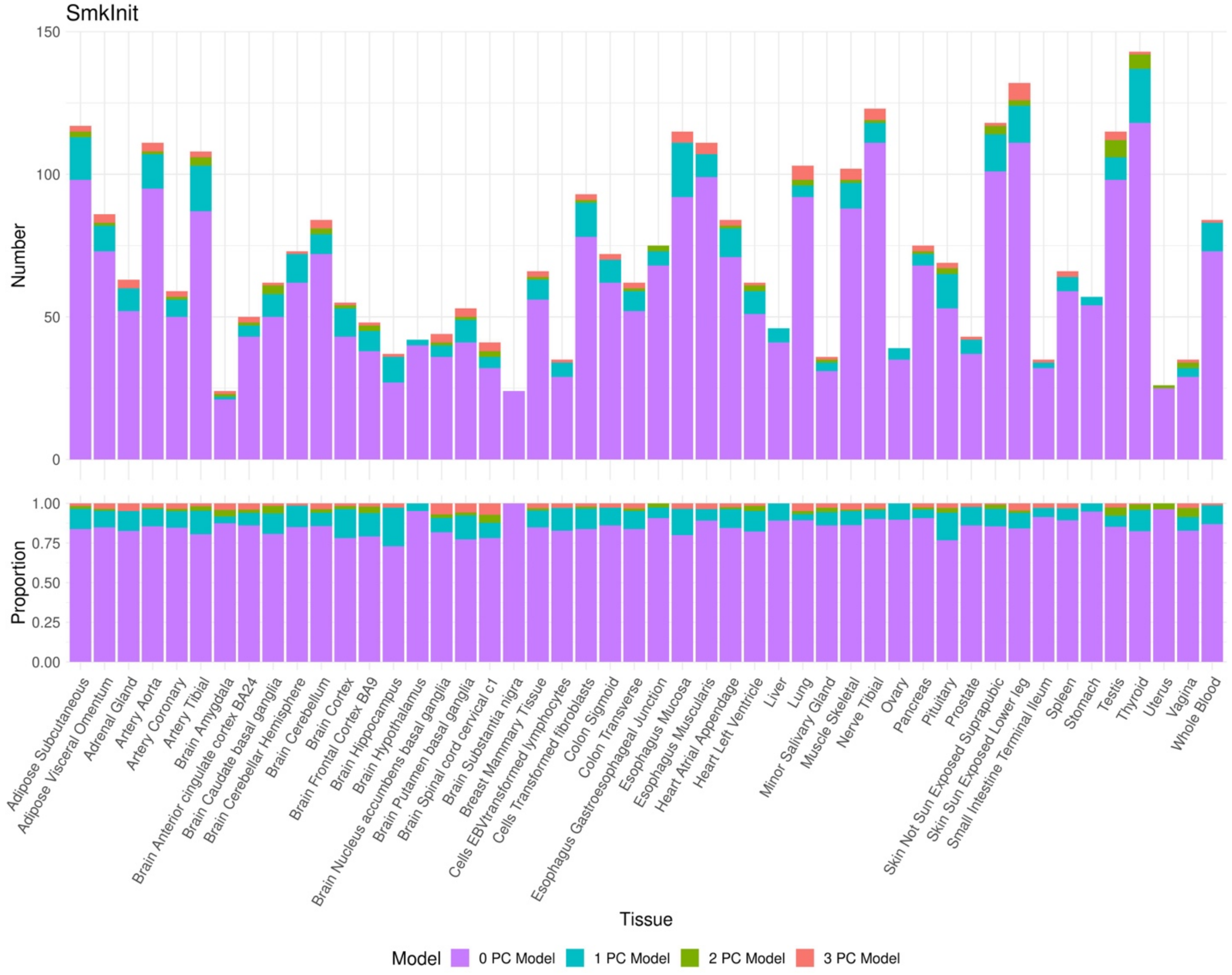
SmkInit
Number
150
100
50
0
Proportion
1.00
0.75
0.50
0.25
0.00
Adipose Subcutaneous
Adipose Visceral Omentum
Adrenal Gland
Artery Aorta
Artery Coronary
Artery Tibial
Brain Amygdala
Brain Anterior cingulate cortex BA24
Brain Caudate basal ganglia
Brain Cerebellar Hemisphere
Brain Cerebellum
Brain Cortex
Brain Frontal Cortex BA9
Brain Hippocampus
Brain Hypothalamus
Brain Nucleus accumbens basal ganglia
Brain Putamen basal ganglia
Brain Spinal cord cervical c1
Brain Substantia nigra
Breast Mammary Tissue
Cells EBVtransformed lymphocytes
Cells Transformed fibroblasts
Colon Sigmoid
Colon Transverse
Esophagus Gastroesophageal Junction
Esophagus Mucosa
Esophagus Muscularis
Heart Atrial Appendage
Heart Left Ventricle
Liver
Lung
Minor Salivary Gland
Muscle Skeletal
Nerve Tibial
Ovary
Pancreas
Pituitary
Prostate
Skin Not Sun Exposed Suprapubic
Skin Sun Exposed Lower leg
Small Intestine Terminal Ileum
Spleen
Stomach
Testis
Thyroid
Uterus
Vagina
Whole Blood
Tissue
Model
0 PC Model
1 PC Model
2 PC Model
3 PC Model

(c)

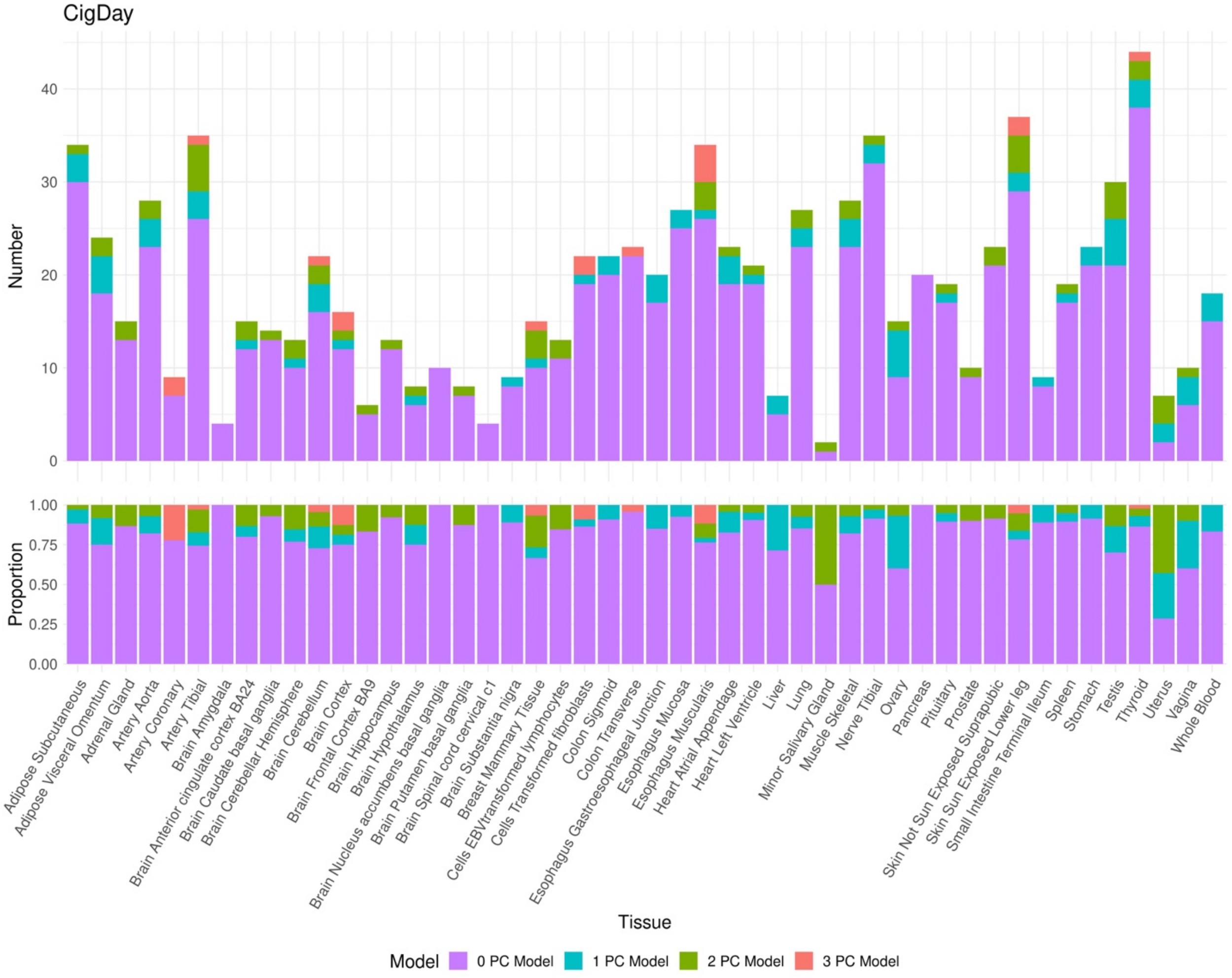

CigDay
Number
Proportion
Tissue
Model
0 PC Model
1 PC Model
2 PC Model
3 PC Model
Adipose Subcutaneous
Adipose Visceral Omentum
Adrenal Gland
Artery Aorta
Artery Coronary
Artery Tibial
Brain Amygdala
Brain Anterior cingulate cortex BA24
Brain Caudate basal ganglia
Brain Cerebellar Hemisphere
Brain Cerebellum
Brain Cortex
Brain Frontal Cortex BA9
Brain Hippocampus
Brain Hypothalamus
Brain Nucleus accumbens basal ganglia
Brain Putamen basal ganglia
Brain Spinal cord cervical c1
Brain Substantia nigra
Breast Mammary Tissue
Cells EBVtransformed lymphocytes
Cells Transformed fibroblasts
Colon Sigmoid
Colon Transverse
Esophagus Gastroesophageal Junction
Esophagus Mucosa
Esophagus Muscularis
Heart Atrial Appendage
Heart Left Ventricle
Liver
Lung
Minor Salivary Gland
Muscle Skeletal
Nerve Tibial
Ovary
Pancreas
Pituitary
Prostate
Skin Not Sun Exposed Suprapubic
Skin Sun Exposed Lower leg
Small Intestine Terminal Ileum
Spleen
Stomach
Testis
Thyroid
Uterus
Vagina
Whole Blood

(d)

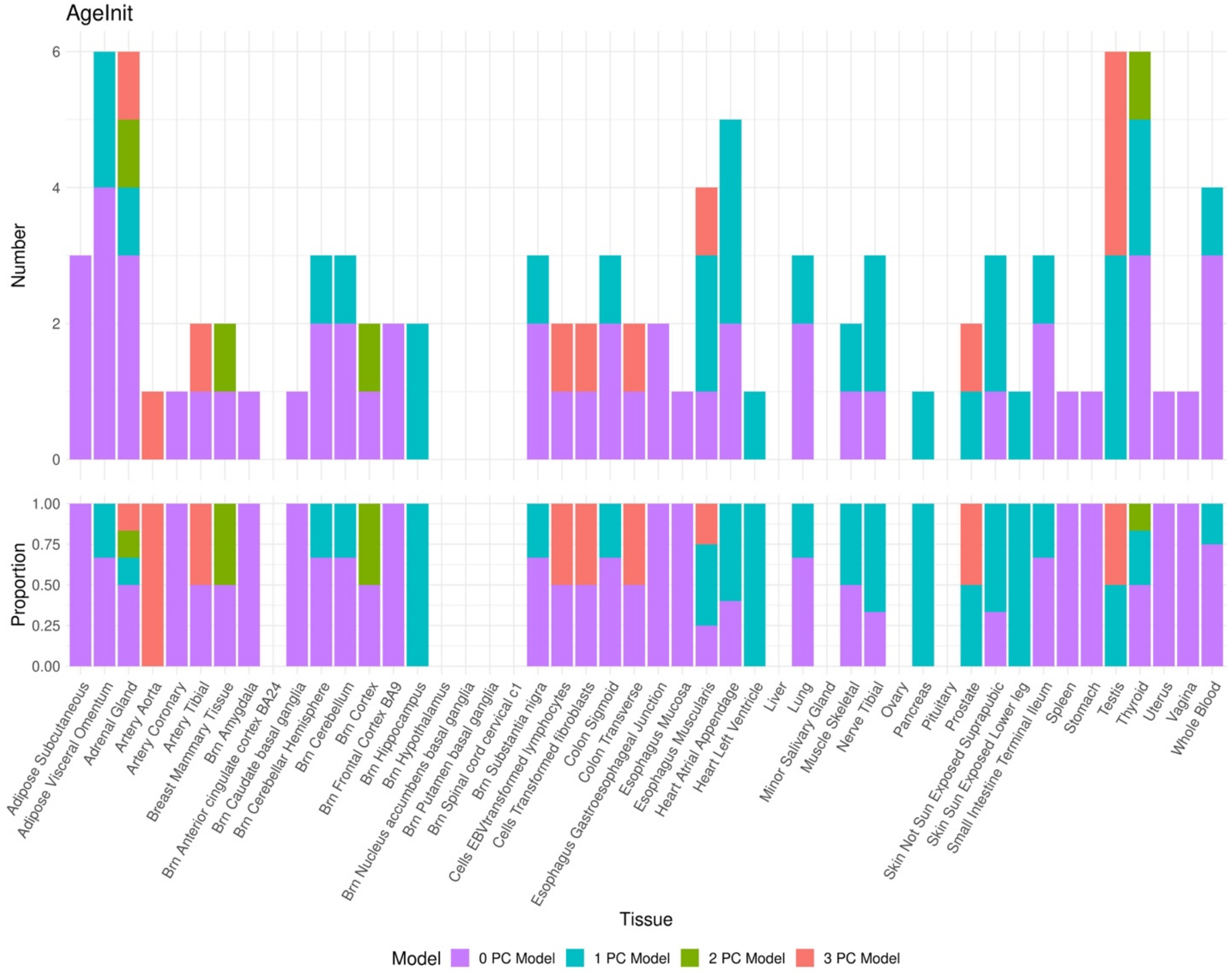

AgeInit
Number
Proportion
Tissue
Adipose Subcutaneous
Adipose Visceral Omentum
Adrenal Gland
Artery Aorta
Artery Coronary
Artery Tibial
Breast Mammary Tissue
Brn Amygdala
Brn Anterior cingulate cortex BA24
Brn Caudate basal ganglia
Brn Cerebellar Hemisphere
Brn Cerebellum
Brn Cortex
Brn Frontal Cortex BA9
Brn Hippocampus
Brn Hypothalamus
Brn Nucleus accumbens basal ganglia
Brn Putamen basal ganglia
Brn Spinal cord cervical c1
Brn Substantia nigra
Cells EBVtransformed lymphocytes
Cells Transformed fibroblasts
Colon Sigmoid
Colon Transverse
Esophagus Gastroesophageal Junction
Esophagus Mucosa
Esophagus Muscularis
Heart Atrial Appendage
Heart Left Ventricle
Liver
Lung
Minor Salivary Gland
Muscle Skeletal
Nerve Tibial
Ovary
Pancreas
Pituitary
Prostate
Skin Not Sun Exposed Suprapubic
Skin Sun Exposed Lower leg
Small Intestine Terminal Ileum
Spleen
Stomach
Testis
Thyroid
Uterus
Vagina
Whole Blood
Model
0 PC Model
1 PC Model
2 PC Model
3 PC Model

Supplementary Figure 6: Fine mapping results for genes *HEY1*, *ASIP*, and *PTPRD*. For each gene, we displayed the posterior inclusion probability (**PIP**), -log10(two-sided p-value), and the regional averaged correlation between genes within 1 million basepairs surrounding the top gene.

Panel a: HEY1 in Brain Hypothalamus with CigDay

Panel b: ASIP in Brain Cortex with CigDay

Panel c: PTPRID in Artery Aorta with SmkInit

a

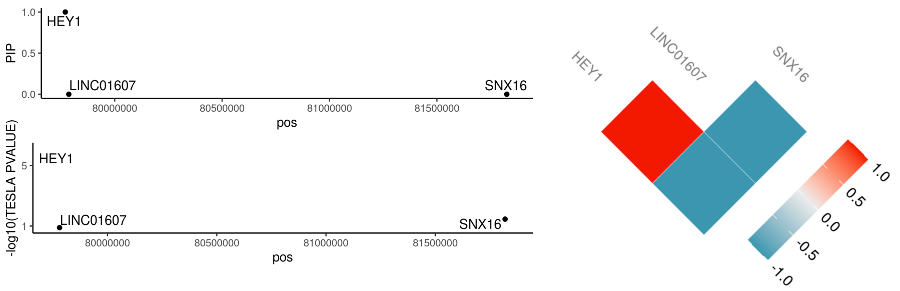

b

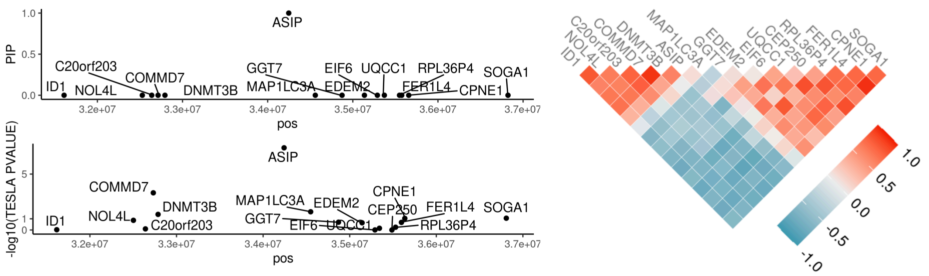

c

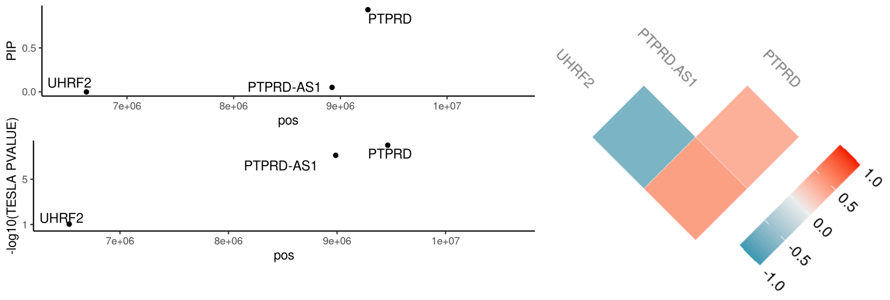

Supplementary Figure 7: TESLA hits were enriched in distinct pathways across 13 brain tissues for Cigday. The pathways enriched with TESLA hits differ between 13 brain tissues. We used REVIGO to reduce redundant GO terms and facilitate visualization of enrichment results. For each tissue, we highlighted GO terms that contains the largest portions of enriched pathways. The brain figures are generated by the R package ggseg[1].

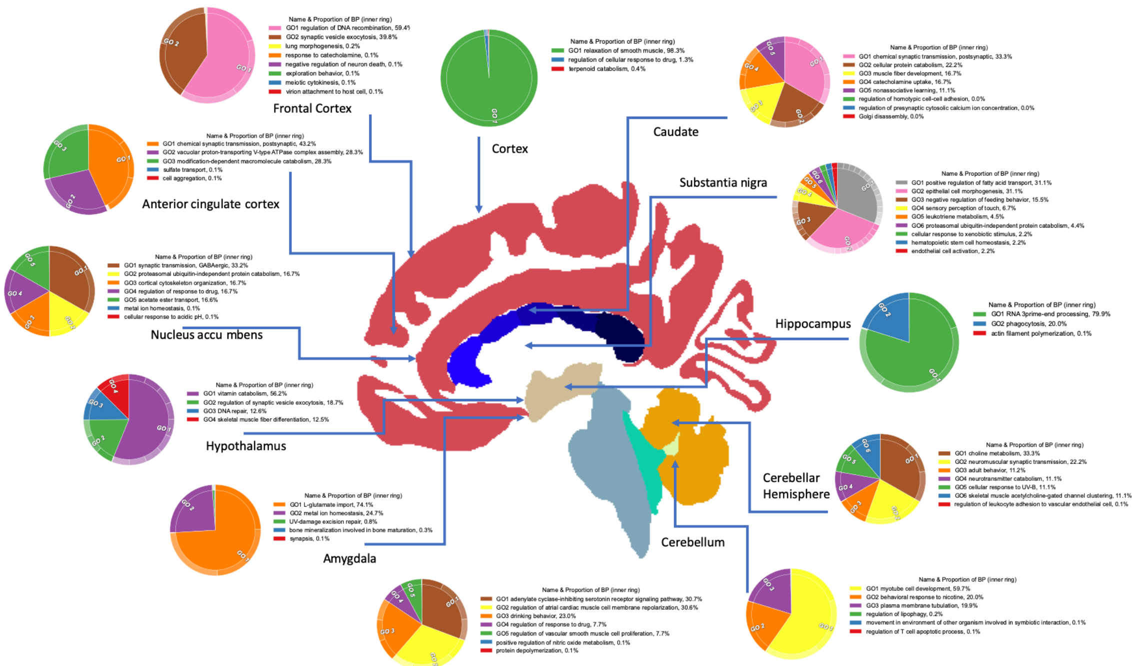

Supplementary Figure 8: **Cell type enrichment in 13 brain tissues with smoking traits.** We created cell type specific gene sets using top 10% more highly expressed genes from each cell type, and tested if these tissue-specific gene sets are enriched with TESLA hits. Panels a-d represent enrichment analysis results for AgeInit, CigDay, SmkInit, and SmkCes respectively. Enrichment p-values (two-sided) were shown for each cell type tested. Cell types with significant two-sided p-values after multiple testing correction is were shown in red, and the ones with insignificant two-sided p-values were shown in blue.

(a)

Smoking trait: AgeInit

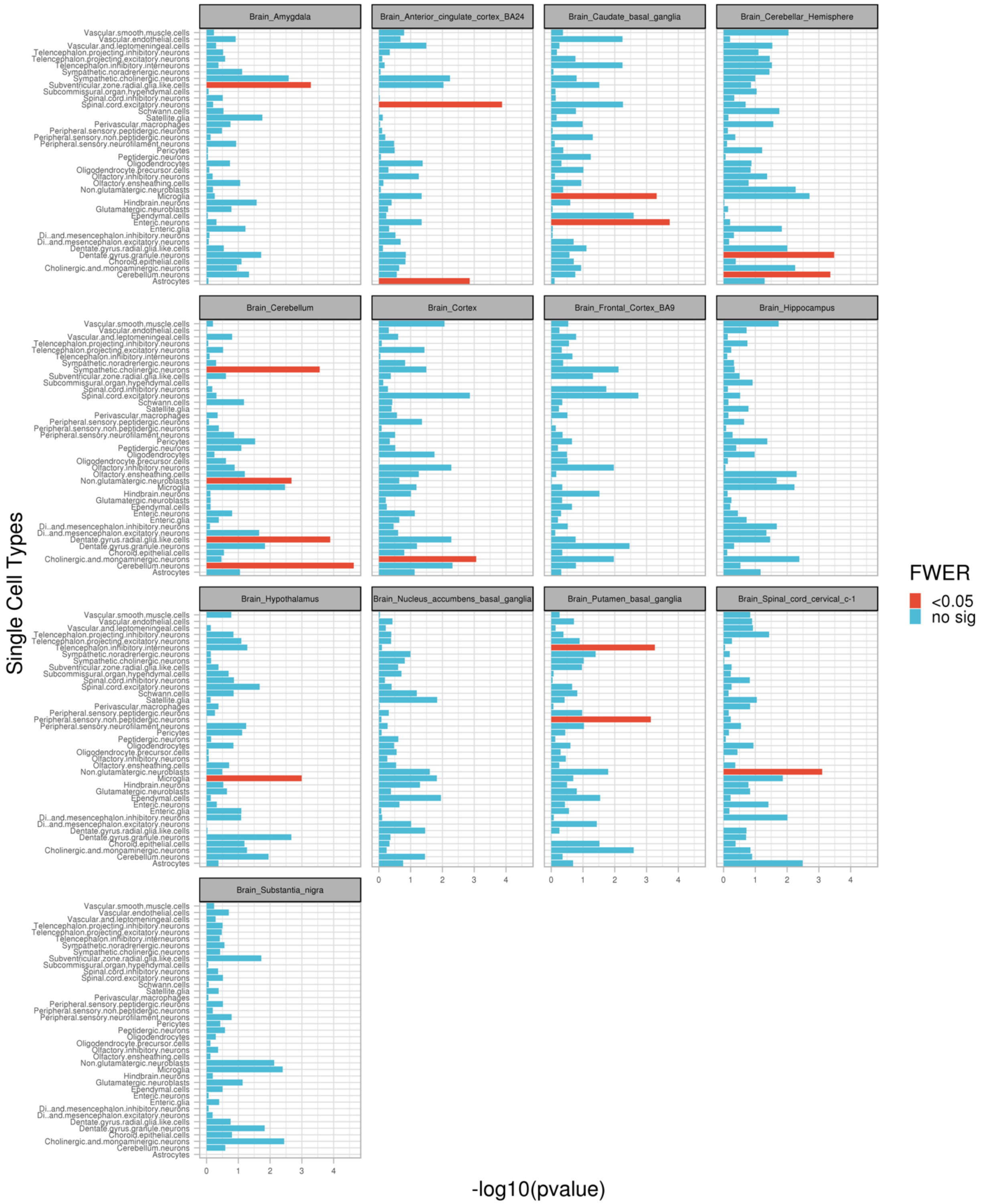

(b)

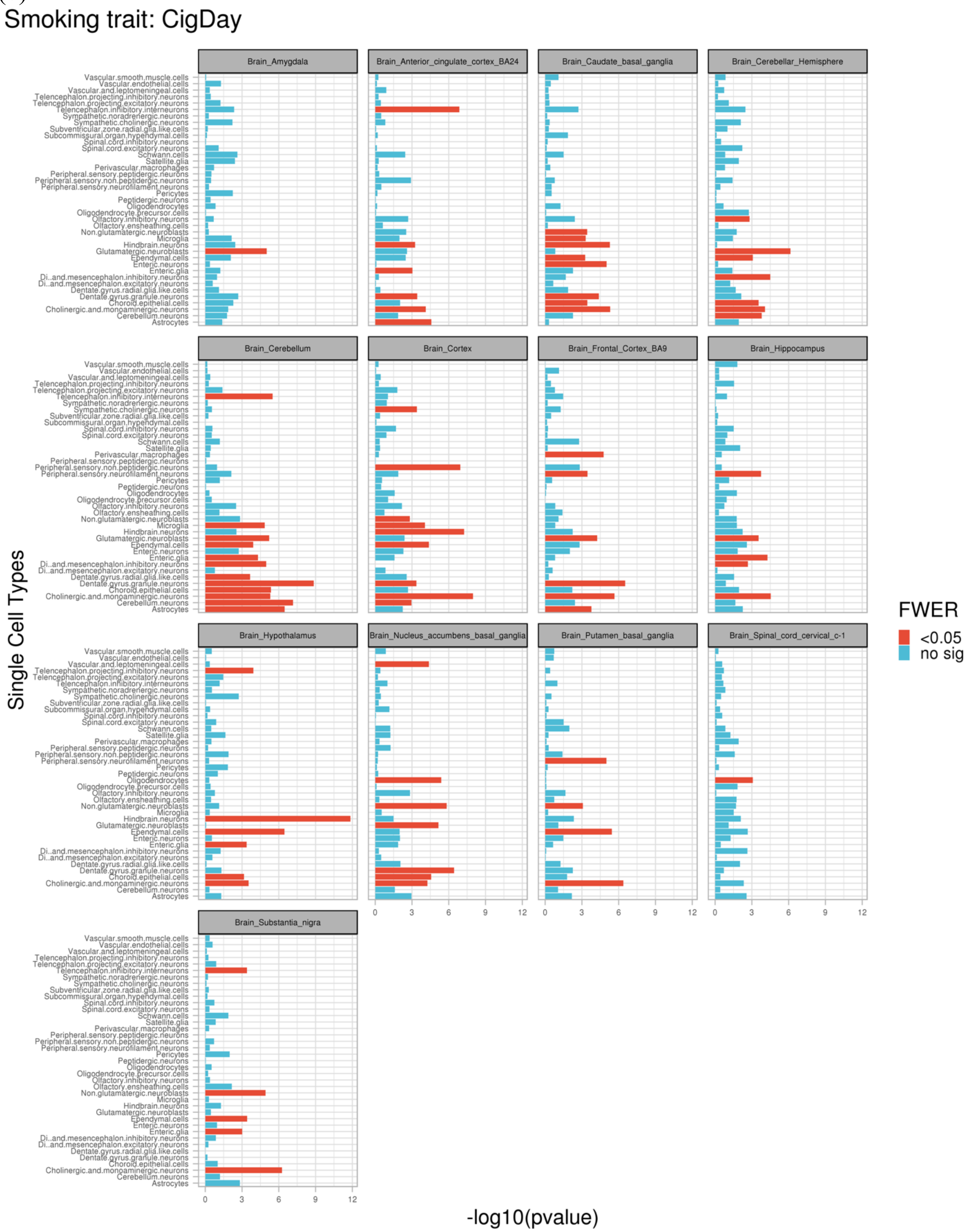

Smoking trait: CigDay
Brain_Amygdala
Brain_Anterior_cingulate_cortex_BA24
Brain_Caudate_basal_ganglia
Brain_Cerebellar_Hemisphere
Brain_Cerebellum
Brain_Cortex
Brain_Frontal_Cortex_BA9
Brain_Hippocampus
Brain_Hypothalamus
Brain_Nucleus_accumbens_basal_ganglia
Brain_Putamen_basal_ganglia
Brain_Spinal_cord_cervical_c-1
Brain_Substantia_nigra
Single Cell Types
-log10(pvalue)
FWER
<0.05
no sig
0
3
6
9
12

(c)

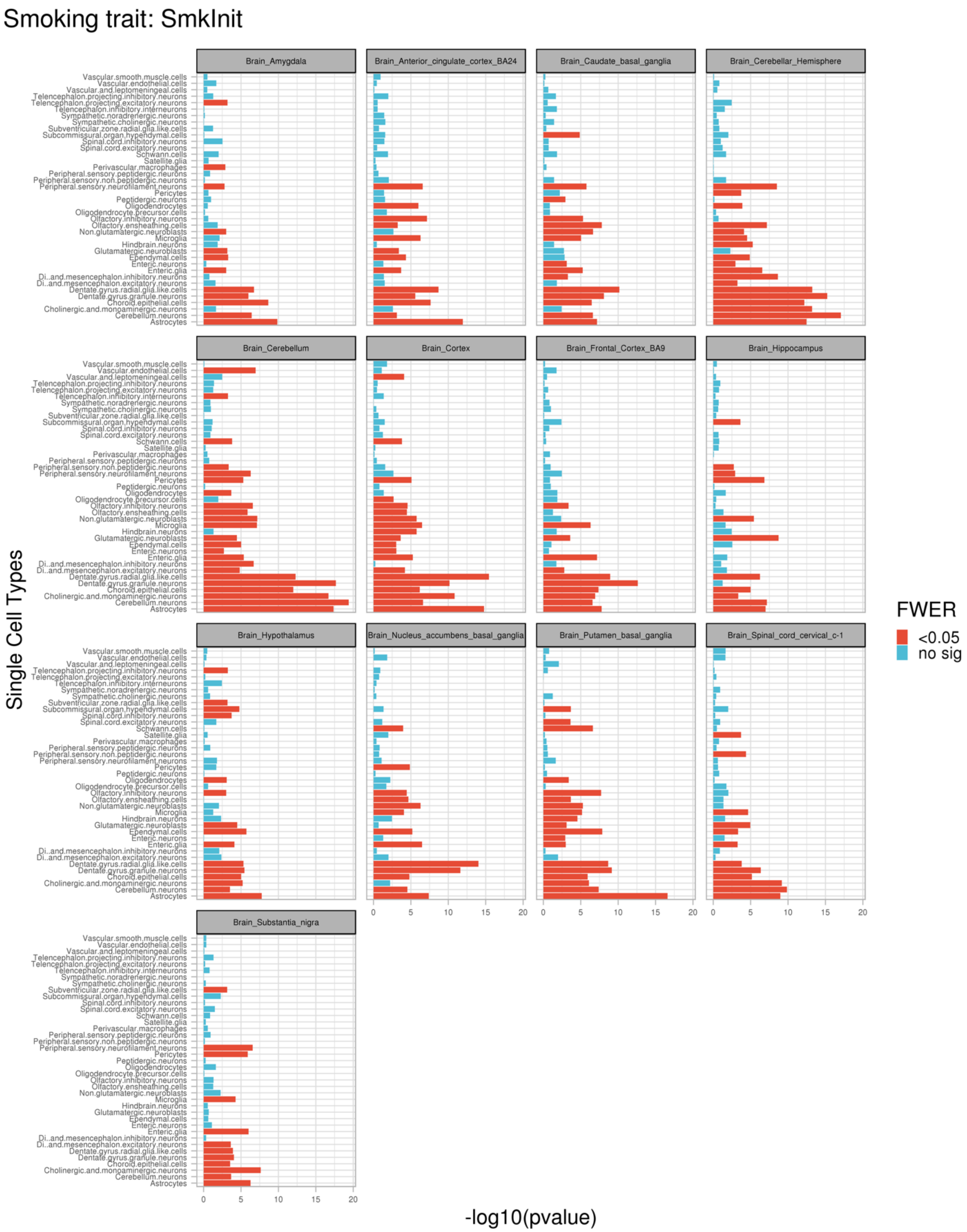
Smoking trait: SmkInit
Brain_Amygdala
Brain_Anterior_cingulate_cortex_BA24
Brain_Caudate_basal_ganglia
Brain_Cerebellar_Hemisphere
Brain_Cerebellum
Brain_Cortex
Brain_Frontal_Cortex_BA9
Brain_Hippocampus
Brain_Hypothalamus
Brain_Nucleus_accumbens_basal_ganglia
Brain_Putamen_basal_ganglia
Brain_Spinal_cord_cervical_c-1
Brain_Substantia_nigra
Vascular.smooth.muscle.cells
Vascular.endothelial.cells
Vascular.and.leptomeningeal.cells
Telencephalon.projecting.inhibitory.neurons
Telencephalon.projecting.excitatory.neurons
Telencephalon.inhibitory.interneurons
Sympathetic.noradrenergic.neurons
Sympathetic.cholinergic.neurons
Subventricular.zone.radial.glia.like.cells
Subcommissural.organ.hypendymal.cells
Spinal.cord.inhibitory.neurons
Spinal.cord.excitatory.neurons
Schwann.cells
Satellite.glia
Perivascular.macrophages
Peripheral.sensory.peptidergic.neurons
Peripheral.sensory.non.peptidergic.neurons
Peripheral.sensory.neurofilament.neurons
Pericytes
Peptidergic.neurons
Oligodendrocytes
Oligodendrocyte.precursor.cells
Olfactory.inhibitory.neurons
Olfactory.ensheathing.cells
Non.glutamatergic.neuroblasts
Microglia
Hindbrain.neurons
Glutamatergic.neuroblasts
Ependymal.cells
Enteric.neurons
Enteric.glia
Di..and.mesencephalon.inhibitory.neurons
Di..and.mesencephalon.excitatory.neurons
Dentate.gyrus.radial.glia.like.cells
Dentate.gyrus.granule.neurons
Choroid.epithelial.cells
Cholinergic.and.monoaminergic.neurons
Cerebellum.neurons
Astrocytes
Single Cell Types
0
5
10
15
20
-log10(pvalue)
FWER
<0.05
no sig

(d)

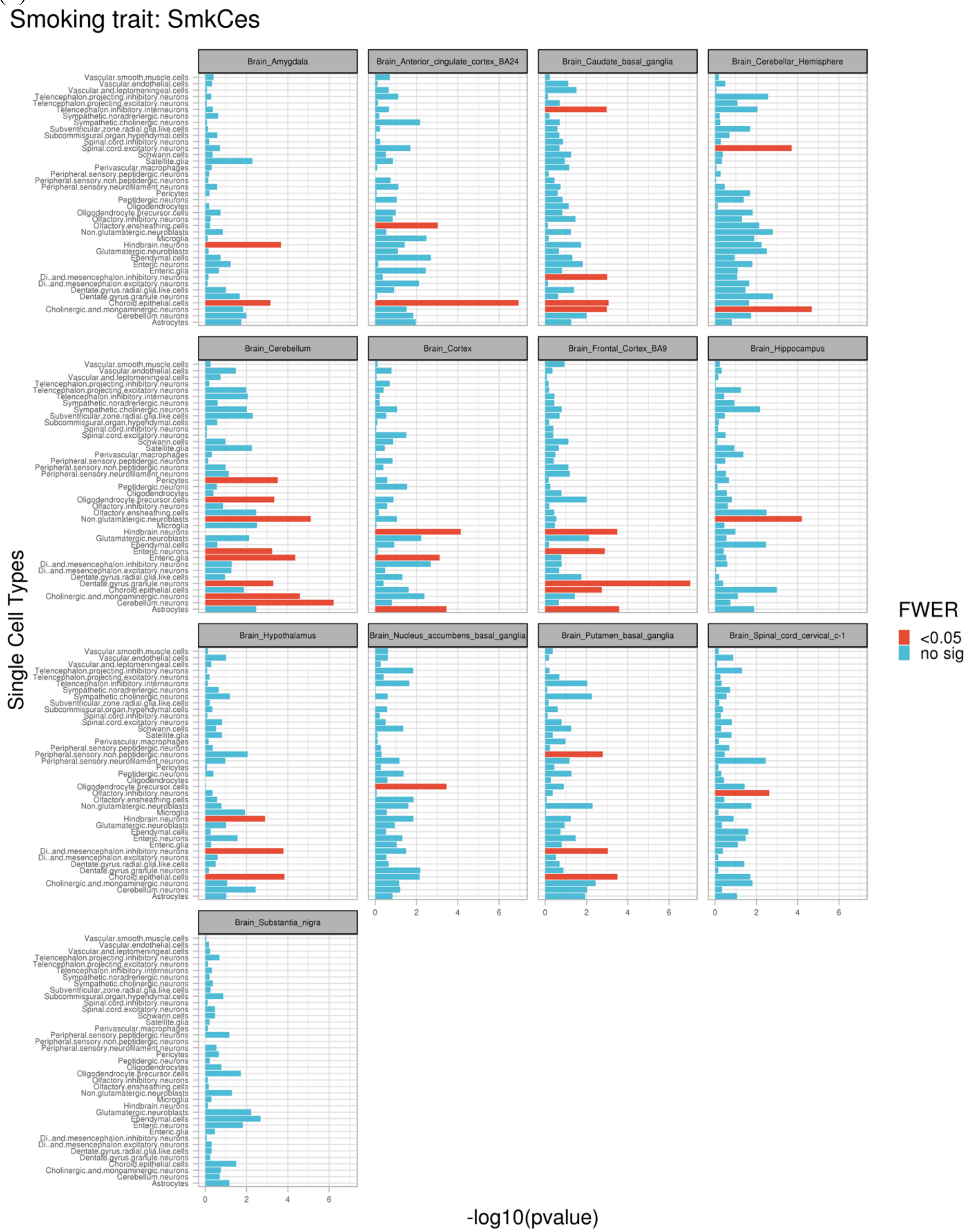
Smoking trait: SmkCes
Brain_Amygdala
Brain_Anterior_cingulate_cortex_BA24
Brain_Caudate_basal_ganglia
Brain_Cerebellar_Hemisphere
Brain_Cerebellum
Brain_Cortex
Brain_Frontal_Cortex_BA9
Brain_Hippocampus
Brain_Hypothalamus
Brain_Nucleus_accumbens_basal_ganglia
Brain_Putamen_basal_ganglia
Brain_Spinal_cord_cervical_c-1
Brain_Substantia_nigra
Vascular.smooth.muscle.cells
Vascular.endothelial.cells
Vascular.and.leptomeningeal.cells
Telencephalon.projecting.inhibitory.neurons
Telencephalon.projecting.excitatory.neurons
Telencephalon.inhibitory.interneurons
Sympathetic.noradrenergic.neurons
Sympathetic.cholinergic.neurons
Subventricular.zone.radial.glia.like.cells
Subcommissural.organ.hypendymal.cells
Spinal.cord.inhibitory.neurons
Spinal.cord.excitatory.neurons
Schwann.cells
Satellite.glia
Perivascular.macrophages
Peripheral.sensory.peptidergic.neurons
Peripheral.sensory.non.peptidergic.neurons
Peripheral.sensory.neurofilament.neurons
Pericytes
Peptidergic.neurons
Oligodendrocytes
Oligodendrocyte.precursor.cells
Olfactory.inhibitory.neurons
Olfactory.ensheathing.cells
Non.glutamatergic.neuroblasts
Microglia
Hindbrain.neurons
Glutamatergic.neuroblasts
Ependymal.cells
Enteric.neurons
Enteric.glia
Di..and.mesencephalon.inhibitory.neurons
Di..and.mesencephalon.excitatory.neurons
Dentate.gyrus.radial.glia.like.cells
Dentate.gyrus.granule.neurons
Choroid.epithelial.cells
Cholinergic.and.monoaminergic.neurons
Cerebellum.neurons
Astrocytes
Single Cell Types
-log10(pvalue)
0
2
4
6
FWER
<0.05
no sig

Supplementary Figure 9: Sanky plot of drug pathway enrichment analysis. We visualize enrichment results, linking smoking traits to mechanisms of action by the drug target genes and drug categories. The widths of the bands between trait and pathways represent the fractions of TESLA hits that belong to a given drug pathway. The widths of the bands between pathways and drugs indications represent the fraction of pathways that are targeted by the drugs with certain indications.

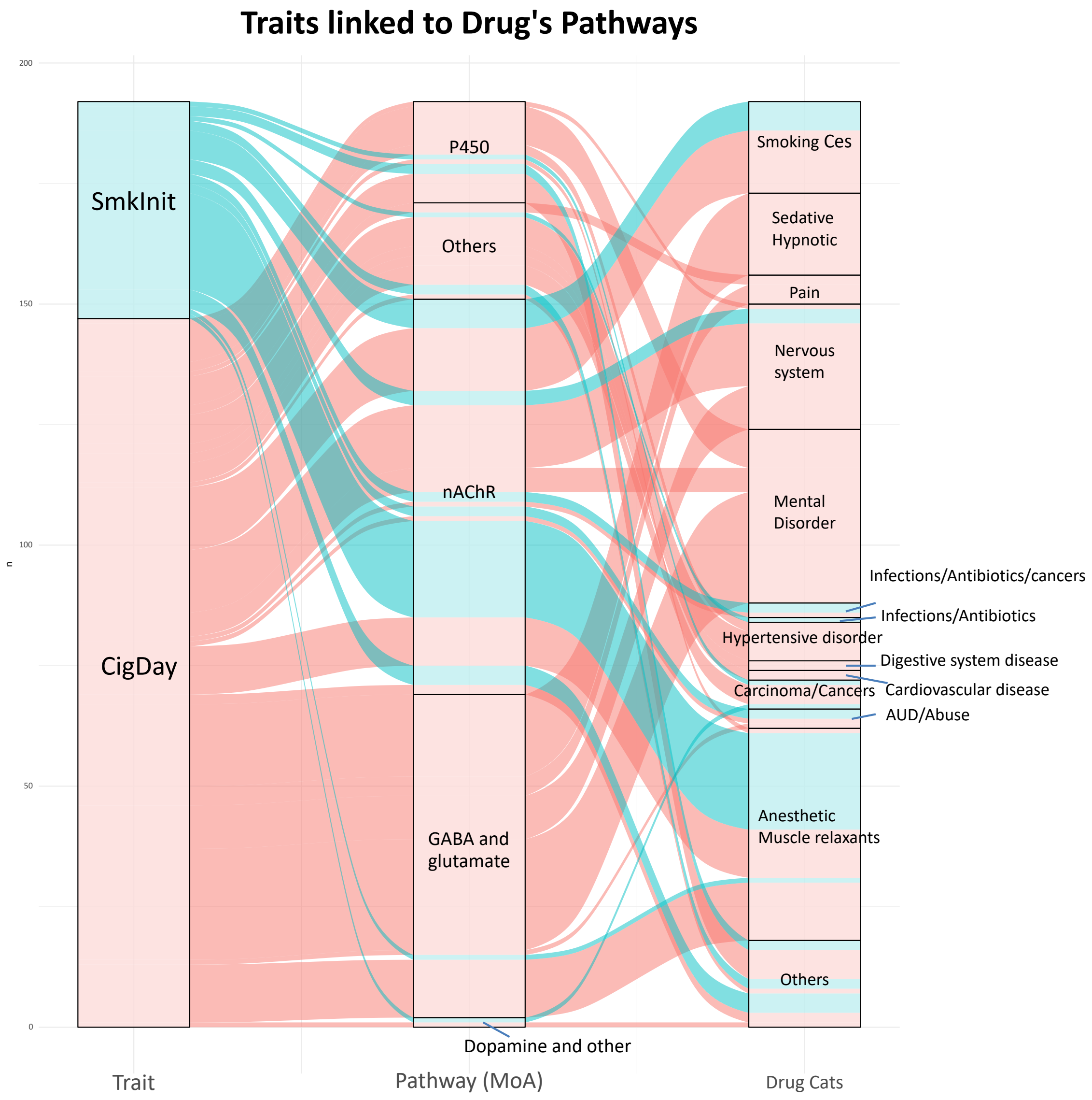

Supplementary Tables

Supplementary Table 1: Phenotypic models used in simulations. For each gene, we randomly pick one gene expression prediction model from whole blood tissue in PrediXcan database and use the weights to simulate phenotypic effects in samples of European ancestry. The genotypes for each individual were simulated by pairing two randomly chosen haplotypes that consist of the eQTL SNPs. The genetic variants (G_j) are assumed to influence phenotypes (Y) via their effects on the gene expression levels (E). We assumed different extent of heterogeneities of phenotypic effect across populations. Specifically, four scenarios were considered: 1) **Homogeneous effect model**: phenotypic effects were homogeneous between ancestries; 2) **European effect model**: phenotypic effects were only present in European populations; 3) **Eurasia effect model**: phenotypic effects were present in European and Asian population; 4) **Heterogeneous effect model**: the genetic variants have different effects on gene expression levels in different ancestries. The eQTL effects in European ancestry is based on PrediXcan database and eQTL effects in non-European populations are simulated based on $w_j^{ASN}, w_j^{AFR} \sim N\left(0, var\left(w_j^{EUR}\right)\right)$. The eQTL effects in different ancestries can be of different directions; 5) **Admixed effect model**: Only alleles of European ancestry influence phenotype (in European samples and in African American samples).

**Ancestry**	**Phenotypic Model**				
	**Homogeneous**	**Ancestry Specific**			**Admixed**
		**EUR-Only**	**Eurasian**	**Heterogeneous**	
**European**	$G_j \xrightarrow{w_j^{EUR}} E \xrightarrow{\theta} Y$	$G_j \xrightarrow{w_j^{EUR}} E \xrightarrow{c} Y$	$G_j \xrightarrow{w_j^{EUR}} E \xrightarrow{c} Y$	$G_j \xrightarrow{w_j^{EUR}} E \xrightarrow{c} Y$	$G_j^* \xrightarrow{w_j^{EUR}} E \xrightarrow{c} Y$
**African**		$G_j \overset{0}{\nrightarrow} Y$	$G_j \overset{0}{\nrightarrow} Y$	$G_j \xrightarrow{w_j^{AFR}} E \xrightarrow{c} Y$	
**Asian**		$G_j \overset{0}{\nrightarrow} Y$	$G_j \xrightarrow{w_j^{EUR}} E \xrightarrow{c} Y$	$G_j \xrightarrow{w_j^{ASN}} E \xrightarrow{c} Y$	

G_j^* measures the number of alleles of European ancestry in each variant site j for an admixed individual.

Supplementary Table 2: Type I error and power in samples with different fraction of non-European samples. We simulated data using the same method as Table 1, but varied the fraction of non-European cohorts in the sample from 20% to 80%. Half of the non-European cohorts come from African American ancestry and the other half come from East Asian Ancestry. Type I error and power were evaluated based on statistical significance level $\alpha = 2.5 \times 10^{-6}$ using 100 million replicates in each scenario.

Scenario			Method							
Model	Expression Effect	Percentage of EUR Cohorts	TESLA Power (SD)		EUR-TWAS Power (SD)		FE-TWAS Power (SD)		RE-TWAS Power (SD)	
Admixed	0.25	80%	0.71	0.000045	0.66	0.000047	0.68	0.000047	0.63	0.000048
		60%	0.65	0.000048	0.58	0.000049	0.61	0.000049	0.55	0.000050
		40%	0.58	0.000049	0.49	0.000050	0.55	0.000050	0.46	0.000050
		20%	0.41	0.000049	0.32	0.000047	0.37	0.000048	0.31	0.000046
	0.33	80%	0.83	0.000038	0.78	0.000041	0.79	0.000041	0.75	0.000043
		60%	0.79	0.000041	0.74	0.000044	0.75	0.000043	0.7	0.000046
		40%	0.7	0.000046	0.63	0.000048	0.66	0.000047	0.58	0.000049
		20%	0.55	0.000050	0.47	0.000050	0.5	0.000050	0.44	0.000050
	0.50	80%	0.88	0.000032	0.84	0.000037	0.84	0.000037	0.82	0.000038
		60%	0.84	0.000037	0.79	0.000041	0.8	0.000040	0.76	0.000043
		40%	0.79	0.000041	0.73	0.000044	0.74	0.000044	0.68	0.000047
		20%	0.65	0.000048	0.57	0.000050	0.59	0.000049	0.52	0.000050
European	0.25	80%	0.67	0.000047	0.68	0.000047	0.63	0.000048	0.61	0.000049
		60%	0.61	0.000049	0.63	0.000048	0.52	0.000050	0.49	0.000050
		40%	0.51	0.000050	0.54	0.000050	0.34	0.000047	0.32	0.000047
		20%	0.32	0.000047	0.36	0.000048	0.11	0.000031	0.11	0.000031
	0.33	80%	0.78	0.000041	0.78	0.000041	0.74	0.000044	0.72	0.000045
		60%	0.73	0.000044	0.74	0.000044	0.65	0.000048	0.61	0.000049
		40%	0.64	0.000048	0.66	0.000047	0.48	0.000050	0.44	0.000050
		20%	0.44	0.000050	0.49	0.000050	0.17	0.000038	0.16	0.000037
	0.50	80%	0.85	0.000036	0.85	0.000036	0.83	0.000038	0.8	0.000040
		60%	0.78	0.000041	0.8	0.000040	0.71	0.000045	0.67	0.000047
		40%	0.71	0.000045	0.74	0.000044	0.56	0.000050	0.51	0.000050
		20%	0.54	0.000050	0.57	0.000050	0.23	0.000042	0.2	0.000040
Eurasia	0.25	80%	0.75	0.000043	0.68	0.000047	0.71	0.000045	0.68	0.000047
		60%	0.75	0.000043	0.61	0.000049	0.71	0.000045	0.67	0.000047
		40%	0.78	0.000041	0.56	0.000050	0.71	0.000045	0.67	0.000047
		20%	0.8	0.000040	0.36	0.000048	0.72	0.000045	0.66	0.000047
	0.33	80%	0.87	0.000034	0.79	0.000041	0.83	0.000038	0.8	0.000040
		60%	0.88	0.000032	0.74	0.000044	0.83	0.000038	0.8	0.000040
		40%	0.88	0.000032	0.67	0.000047	0.83	0.000038	0.78	0.000041
		20%	0.9	0.000030	0.51	0.000050	0.84	0.000037	0.78	0.000041
	0.50	80%	0.91	0.000029	0.84	0.000037	0.86	0.000035	0.84	0.000037
		60%	0.91	0.000029	0.77	0.000042	0.86	0.000035	0.83	0.000038
		40%	0.93	0.000026	0.75	0.000043	0.88	0.000032	0.84	0.000037
		20%	0.94	0.000024	0.55	0.000050	0.87	0.000034	0.82	0.000038
Homogeneous	0.25	80%	0.71	0.000045	0.67	0.000047	0.72	0.000045	0.7	0.000046
		60%	0.69	0.000046	0.62	0.000049	0.72	0.000045	0.69	0.000046
		40%	0.72	0.000045	0.55	0.000050	0.75	0.000043	0.71	0.000045
		20%	0.71	0.000045	0.36	0.000048	0.76	0.000043	0.71	0.000045

Scenario			Method							
Model	Expression Effect	Percentage of EUR Cohorts	TESLA Power (SD)		EUR-TWAS Power (SD)		FE-TWAS Power (SD)		RE-TWAS Power (SD)	
	0.33	80%	0.81	0.000039	0.78	0.000041	0.83	0.000038	0.81	0.000039
		60%	0.82	0.000038	0.74	0.000044	0.84	0.000037	0.81	0.000039
		40%	0.84	0.000037	0.67	0.000047	0.85	0.000036	0.81	0.000039
		20%	0.83	0.000038	0.49	0.000050	0.86	0.000035	0.82	0.000038
	0.50	80%	0.86	0.000035	0.84	0.000037	0.87	0.000034	0.86	0.000035
		60%	0.87	0.000034	0.8	0.000040	0.89	0.000031	0.87	0.000034
		40%	0.88	0.000032	0.74	0.000044	0.9	0.000030	0.87	0.000034
		20%	0.89	0.000031	0.59	0.000049	0.91	0.000029	0.87	0.000034
Heterogeneous	0.25	80%	0.70	0.000046	0.67	0.000047	0.67	0.000047	0.64	0.000048
		60%	0.61	0.000049	0.58	0.000049	0.57	0.00005	0.54	0.00005
		40%	0.63	0.000048	0.55	0.00005	0.57	0.00005	0.54	0.00005
		20%	0.54	0.00005	0.35	0.000048	0.46	0.00005	0.43	0.00005
	0.33	80%	0.86	0.000035	0.83	0.000038	0.82	0.000038	0.79	0.000041
		60%	0.78	0.000041	0.73	0.000044	0.75	0.000043	0.73	0.000044
		40%	0.76	0.000043	0.67	0.000047	0.72	0.000045	0.69	0.000046
		20%	0.72	0.000045	0.47	0.00005	0.66	0.000047	0.59	0.000049
	0.50	80%	0.91	0.000029	0.87	0.000034	0.87	0.000034	0.84	0.000037
		60%	0.82	0.000038	0.79	0.000041	0.78	0.000041	0.77	0.000042
		40%	0.77	0.000042	0.69	0.000046	0.73	0.000044	0.71	0.000045
		20%	0.81	0.000039	0.59	0.000049	0.75	0.000043	0.69	0.000046
Null*	0.00	80%	2.3×10^{-6}	1.51657	2.3×10^{-6}	1.51657	2.1×10^{-6}	1.44914	2.1×10^{-6}	1.449
		60%	2.3×10^{-6}	1.51657	2.1×10^{-6}	1.44914	2.5×10^{-6}	1.58114	2.2×10^{-6}	1.483
		40%	2.3×10^{-6}	1.51657	2.4×10^{-6}	1.54919	2.5×10^{-6}	1.58114	2.7×10^{-6}	1.643
		20%	2.3×10^{-6}	1.51657	2.7×10^{-6}	1.64317	2.4×10^{-6}	1.54919	2.9×10^{-6}	1.702
		80%	2.3×10^{-6}	1.51657	2.3×10^{-6}	1.51657	2.3×10^{-6}	1.51657	2.1×10^{-6}	1.449
		60%	2.2×10^{-6}	1.48324	2.4×10^{-6}	1.54919	2.3×10^{-6}	1.51657	2.4×10^{-6}	1.549
		40%	2.4×10^{-6}	1.54919	2.3×10^{-6}	1.51657	2.3×10^{-6}	1.51657	2.5×10^{-6}	1.581
		20%	2.7×10^{-6}	1.64317	2.8×10^{-6}	1.67332	2.7×10^{-6}	1.64317	2.8×10^{-6}	1.673
		80%	2.4×10^{-6}	1.54919	2.6×10^{-6}	1.61245	2.4×10^{-6}	1.54919	2.4×10^{-6}	1.549
		60%	2.6×10^{-6}	1.61245	2.8×10^{-6}	1.67332	2.8×10^{-6}	1.67332	2.5×10^{-6}	1.581
		40%	2.6×10^{-6}	1.61245	2.5×10^{-6}	1.58114	2.8×10^{-6}	1.67332	2.6×10^{-6}	1.612
		20%	2.3×10^{-6}	1.51657	2.4×10^{-6}	1.54919	2.3×10^{-6}	1.51657	2.9×10^{-6}	1.702

* Due to the extremely small values, the scale of SD if type I error is based on $\times10^{-7}$

Supplementary Table 3: Summary of cohort samples by phenotypes. The ancestries for the cohorts were determined based upon the principal components (PC) of genome-wide allele frequencies of each cohort and 1000 Genomes Project ancestry groups. The ancestry of each cohort was assigned according to the closest 1000 Genomes Project ancestry on the PC map based on Euclidean distance.

Study Name	Phenotype				Ancestry
	AgeInit	CigDay	SmkCes	SmkInit	
**23andMe3**	78437	73380	234398	599289	EUR
**AACAC**	-	-	258	389	AFR
**ALSPAC**	4691	4314	4748	11345	EUR
**AMISH**	238	193	210	849	EUR
**ARIC**	5559	5689	5747	-	EUR
**BAGS**	50	107	95	532	AFR
**BEAGESS**	-	-	2805	4293	EUR
**Boston**	-	65	65	-	EUR
**CADD**	775	523	1002	1192	EUR
**CFS**	-	-	462	833	AMR
**CHS**	-	1495	1553	2848	EUR
**COGEND**	1952	1940	1954	-	EUR
**COPDGene**	6613	6613	6613	-	AMR
**CRA**	34		59	246	AMR
**deCODE**	40314	44505	34820	57097	EUR
**ECLIPSE**	1399	1273	1425	1458	EUR
**EGCUT370CNV**	755	787	822	1758	EUR
**EGCUTEXOME**	1784	1781	1835	4418	EUR
**EGCUTOMNI**	2953	3065	3121	7041	EUR
**EMERGEcataracts**	-	-	1884	3873	EUR
**EMERGEpad**	-	-	1002	1350	EUR
**FHS**	3025	3065	3075	6177	EUR
**FINNTWIN**	509	498	498	1006	EUR
**GARNET**	2081	2001	2072	4190	EUR
**GECCO**	766	733	763	1554	EUR
**GeneSTAR**	707	354	742	1622	AMR
**GENOA**	275	294	525	1252	AFR
**GENSalt**	552	511	580	1695	ASIAN
**GFG**	855	835	933	1829	EUR
**GOLDN**	223	128	261	903	EUR
**HarvardAffymetrix**	2404	2810	4377	7763	EUR
**HarvardHumancore**	2352	2461	4377	7763	EUR
**HCHS**	-	944	1275	2721	AMR
**HIPFX**	1615	1529	1605	3195	EUR
**HRS**	5585	5306	-	9989	EUR
**HUNT**	35311	33705	37964	66716	EUR
**HVH**	-	-	319	680	EUR
**HyperGEN**	802	870	937	1800	AFR
**IPF**	126	127	290	449	EUR
**JHS**	871	998	1048	3236	AFR
**LLS**	479	450	477	1099	EUR
**MCTFR**	-	2535	2808	6181	EUR
**MESA**	1183	1150	1206	2165	AMR
**METSIM**	1500	1507	5504	9607	EUR
**MOPMAP**	1179	1133	1175	2257	EUR
**NESCOG**	-	210	216	477	EUR
**NIKO**	1704	1716	1649	2052	EUR
**NTR**	2955	2725	3107	7266	EUR
**OMG**	168	155	178	540	AFR

**qimr**	4193	4409	-	-	EUR
**QIMR19up**	546	546	548	1238	EUR
**SAFS**	-	-	-	1529	AMR
**sardinia**	2057	2105	2105	5459	EUR
**SARP**	-	-	-	1296	AMR
**Samoan**	371	383	-	1187	Samoan
**STROKE**	-	-	1562	3665	EUR
**THRV**	-	-	573	2039	ASIAN
**UKB**	124590	120744	168970	383631	EUR
**VTE**	-	-	228	356	EUR
**WGHS**	-	-	57	115	EUR
**WHI**	2705	2564	2691	5573	EUR
**Total number of studies**	44	48	56	56	
**Total number of samples**	347243	345231	559573	1261083	

Supplementary Table 4. Gene-trait associations identified across 13 brain tissues. The number of gene-trait associations identified by each method is shown for the four smoking traits across all 13 brain-only tissues.

	Genes identified across 13 brain tissues			
**Trait**	**TESLA**	**FE-TWAS**	**EURO-TWAS**	**RE-TWAS**
**SmkInit**	558 (261, 53)	525 (245, 47)	492 (233, 35)	37 (16, 3)
**SmkCes**	73 (43, 2)	62 (39, 2)	66 (40, 2)	6 (4, 0)
**CigDay**	136 (68, 16)	126 (60, 8)	127 (56, 5)	77 (29, 4)
**AgeInit**	16 (12, 3)	11 (8, 2)	6 (5, 1)	0 (0, 0)
**Total**	783 (384, 74)	724 (352, 59)	691 (334, 43)	120 (49, 7)

* The numbers in the parentheses are unique genes results and novel genes, respectively

Supplementary Table 5 [Excel spreadsheet]: Complete list of novel loci identified by TESLA for SmkInit, SmkCes, AgeInit, and CigDay phenotypes. Genes with TWAS two-sided p-values $< 2.5 \times 10^{-6}$ are deemed statistically significant. A gene x trait association is considered novel it is > 1 million basepairs away from previously reported GWAS hits.

Supplementary Table 6: TESLA identified substantially more loci and novel loci than FE-TWAS, RE-TWAS, and EURO-TWAS using GTEx data and PrediXcan weights under more stringent significance threshold. Genes with TWAS two-sided p-values $< 5 \times 10^{-8}$ (adjusting for testing multiple genes in all 48 tissue) were deemed statistically significant. A gene x trait association was considered novel if it was 1 million basepairs away from previously reported GWAS hits. The numbers of gene x trait associations, the number of unique gene x trait associations (i.e., the gene x trait association that appears in multiple tissues is counted only once), and novel associations are shown for each TWAS method.

	Number of genes identified across 48 tissues (number of novel gene associations) with a more stringent threshold			
**Trait**	**TESLA**	**FE-TWAS**	**RE-TWAS**	**EURO-TWAS**
SmkInit	1523 (456, 57)	1482 (436, 50)	53(19, 2)	1356 (402, 43)
SmkCes	242 (84, 9)	209(72, 7)	6 (5, 0)	215 (72, 6)
CigDay	438 (140, 10)	426 (127, 10)	204 (66, 16)	438 (130, 5)
AgeInit	23 (16, 3)	17 (11, 1)	1 (1, 0)	7 (6, 0)
Total	2226 (696, 79)	2134 (646, 68)	264(91, 18)	2016 (610, 54)

Supplementary Table 7 Comparison of TESLA Results using eQTL datasets of European and African American ancestries from LIBD. We list the number of loci that were identified in each ancestry (with two-sided p-value $< 2.5 \times 10^{-6}$) and the number of loci that also remained significant in the other ancestry.

Trait	Significant Genes using EUR eQTLs	% Loci based on European eQTLs Also Significant using African American eQTLs	Significant Genes using AFR eQTLs	% Loci based on African American eQTLs Also Significant using European eQTLs
SmkInit	65	34.7%	75	29.2%
SmkCes	11	37.5%	16	37.5%
AgeInit	4	0.0%	1	100.0%
CigDay	33	44.0%	30	60.7%

Supplementary Table 8: Comparison between different TWAS methods using LIBD nucleus accumbens eQTL data of European and African American ancestries. Similar to Table 1, we report significant gene trait associations (two-sided $p < 2.5 \times 10^{-6}$), as well as novel associations that were > 1 million basepairs away from reported GWAS sentinel variants (shown in parenthesis).

Trait	EUR-eQTL				AFR-eQTL			
	TESLA	FE-TWAS	RE-TWAS	EURO-TWAS	TESLA	FE-TWAS	RE-TWAS	AFR-TWAS
SmkInit	65 (32)	25 (10)	5 (2)	27(12)	75 (43)	27 (9)	11 (6)	0 (0)
SmkCes	11 (6)	2 (1)	0 (0)	2 (1)	16 (12)	3 (1)	4 (2)	0 (0)
CigDay	33 (19)	24 (13)	6 (2)	24 (11)	30 (15)	33 (15)	13 (5)	0 (0)
AgeInit	4 (4)	0	0 (0)	0 (0)	1 (1)	1 (1)	2 (2)	0 (0)

Supplementary Table 9: The meta-regression models that produce minimal p-values inform the extent of phenotypic effect heterogeneity. TESLA fits multiple meta-regression models with varying number of allele frequency principal components. Based on estimated phenotypic effects in each meta-regression model, we perform TWAS using two-sided tests and combine the results using minimal p-values. The meta-regression model that yields the minimal p-value will inform the extent of phenotypic heterogeneity between ancestries. For example, the meta-regression model with 0 PC included is equivalent to fixed effect meta-analysis. As the first PC separates African and non-African ancestry, the meta-regression model with 1 PC is likely to produce the most significant p-values for genes showing different effects between African American and other ancestries. Here, among genes that reach significance level $\alpha = 2.5 \times 10^{-6}$, we report the fraction and number of different meta-regression models that produce minimal p-values.

	Meta-regression models that produce minimal p-values among genes with significant TESLA p-values								
	**0 PC Model**		**1 PC Model**		**2 PC Model**		**3 PC Model**		**Total**
**Trait**	# of gene	fraction*	# of gene	fraction	# of gene	fraction	# of gene	fraction	
**AgeInit**	16	0.64	7	0.28	0	0.00	2	0.08	25
**CigDay**	166	0.83	17	0.08	14	0.07	4	0.02	201
**SmkCes**	21	0.20	17	0.16	19	0.18	49	0.46	106
**SmkInit**	587	0.85	68	0.10	13	0.02	20	0.03	688
**Total**	790	0.77	109	0.11	46	0.05	75	0.07	**1020**

*: the fraction is calculated as N_l/N_{sig}, where

- N_l is the number of genes where TELSA p-value is significant and the meta-regression model with l PC yields the minimal p-value.
- N_{sig} is the number of genes with significant TESLA p-value.

Supplementary Table 10 [Excel spreadsheet]: Fine mapping of TESLA results. For each trait, we performed fine mapping for TESLA results. We define a locus as a 1 million basepair window surrounding a sentinel gene with most significant two-sided p-values ($<2.5 \times 10^{-6}$). Posterior inclusion probability was calculated for the sentinel gene, and the genes within 90% credible interval were also reported. For secondary association signals, iterative conditional analysis was performed, by conditioning on the most significant gene in the previous iteration and conditional TESLA two-sided p-value and converted Z-scores were used to estimate PIP for secondary signals.

Supplementary Table 11 [Excel spreadsheet]: GO terms enrichment results. We created gene sets based upon pathways in the Gene Ontology database, and performed enrichment analysis using TELSA results. We used parametric bootstrap to control for family-wide error rate (FWER). We reported GO terms with significant two-sided p-values (FWER<0.05), as well as the categories of the GO term. As a sensitivity analysis on the impact of the pathway database used, we also included significant enrichment hits using KEGG, Reactome, and wikiPathways following the same pipeline.

Supplementary Table 12 [Excel spreadsheet]: GO terms enrichment results using MAGMA and samples of European ancestry. We created gene sets based upon pathways in the Gene Ontology database, and performed enrichment analysis using fixed effect meta-analysis results of samples of European ancestry. We reported GO terms with significant two-sided p-values (FWER<0.05), as well as the categories of the GO term.

Supplementary Table 13 [Excel spreadsheet]: Cell types enrichment results. We created cell type specific gene sets using the top 10% of the genes that are most highly expressed in each cell type, and examined if these gene sets are enriched with TESLA hits. We used parametric bootstrap to control for family-wide error rate. Gene sets that are significantly enriched with TESLA hits (i.e. FWER<0.05) point to cell types relevant to smoking traits.

Supplementary Table 14 [Excel spreadsheet]: Drug pathway enrichment results. We created gene sets based upon drug target pathways, and assess if these results were enriched with TESLA hits. We used parametric bootstrap to control for the family-wise error rate (FWER<0.05). Results with significant two-sided enrichment p-values (with FWER<0.05) are reported.

Supplementary Table 15 [Excel spreadsheet]: Genomic control values for cohorts from GWAS and Sequencing Consortium of Alcohol and Nicotine Use (GSCAN) and Trans-Omics Precision Medicine (TOPMed). Genomic control values were separately calculated for common variants (with minor allele frequency >0.01) and rare variants (with minor allele frequency between 0.001 and 0.01).

Supplementary Text

1. Phenotype Definitions

Below we describe the definition of four smoking behavioral phenotypes.

A. *Age of Initiation of Regular Smoking (AgeInit)*

1. Age at which an individual started smoking cigarettes regularly
2. Does not include information about pipes/cigars/chew, or other non-cigarette forms of tobacco use.
3. Measured in a variety of ways:
   a. At what age did you begin smoking regularly?
   b. How long have you smoked combined with the question: what is your current age?

B. *Cigarettes per Day (CigDay)*

1. Defined as the average number of cigarettes smoked per day, either as a current smoker or former smoker, and whether self-rolled or manufactured are smoked (most studies did not distinguish). Individuals who either never smoked, or for whom there is no available data (e.g., someone was a former smoker, but for whom former smoking was never assessed) were set to missing.
2. For studies that collected a quantitative measure of cigarettes per day, where the respondent is free to provide any integer (e.g., 13 cigarettes per day) responses were binned as follows.
   a. 1=1-5
   b. 2 = 6-15
   c. 3 = 16-25
   d. 4 = 26-35
   e. 5=36+
3. For studies with pre-defined bins, the pre-defined bins were used.
4. Does not include information about pipes/cigars/chew, or other non-cigarette forms of tobacco use.
5. Cigarettes per day was measured with a single question for most contributing studies using, for example:
   a. How many cigarettes do you smoke per day?
   b. How many cigarettes did you smoke per day?

C. *Smoking Cessation (SmkCes)*

1. Binary phenotype with current smokers coded as "2" and former smokers coded as "1", and never smokers are coded as missing.
2. Does not include information about pipes/cigars/chew, or other non-cigarette forms of tobacco use.
3. Usually measured through a combination of questions, including:
   a. Do you currently smoke and have you ever smoked regularly?
   b. Do you smoke and have you smoked over 100 cigarettes in your entire life?

D. *Smoking Initiation (SmkInit)*

1. This is a binary phenotype. Any participant reporting ever being a regular smoker in their life (current or former) were coded "2", while any participant who reported never being a regular smoker in their life were coded "1".
2. Does not include information about pipes/cigar/chew, or other non-cigarette forms of tobacco use.
3. This phenotype was measured in a variety of ways according to the reference[2]
   a. Have you smoked over 100 cigarettes over the course of your life?
   b. Have you ever smoked every day for at least a month?
   c. Have you ever smoked regularly?

2. Dataset description

Below, we describe the transcriptomics datasets used in our manuscript.

Genotype Tissue Expression (GTEx) Project Data

We obtained pre-computed gene expression prediction model weights of 48 tissues from the PrediXcan website, which were based on GTEx (version 7)[3].

Lieber Institute for Brain Development (LIBD) Human Brain Repository Data

To complement GTEx nucleus accumbens data, we leveraged RNA-seq and genotype data from postmortem nucleus accumbens samples of physiologically normal human brains. Compared to GTEx data of the same tissue, our data is more ancestrally diverse and includes a greater fraction of African American samples, i.e., 53% individuals are from European (N=104) and 47% from African ancestry (N=94).

We used paired-end, stranded RNA-seq and Illumina genotyping array data from postmortem nucleus accumbens of physiologically normal human brains. Details on their data collection, RNA-seq and genotyping data processing, and quality control (QC) are described in Markunas et al[4].

For eQTL analyses, we included samples with RNA integrity number (RIN) $\geq$6, gene assignment rate (GAR; proportion of reads mapping to a gene annotation) $\geq$0.3, mitochondrial mapping rate (proportion of reads mapping to mitochondrial DNA) $\geq$0.11, and overall mapping rate $\geq$0.5. Among the samples passing these QC filters, lowly expressed genes (genes with $\leq$10% of samples having 1 transcript per million mapped reads (TPM) or $\leq$10% of samples having 10 raw counts) were excluded. Both TPM and raw count values were quantified using the software Salmon version 1.5.2 (https://salmon.readthedocs.io). Across sample normalization was then applied to the raw counts using median-of-ratios normalization in DESeq2 (https://bioconductor.org/packages/release/bioc/html/DESeq2.html), followed by a variance stabilizing transformation. Data processing was done separately by ancestry, resulting in 20,486 genes among European ancestry samples and 20,973 genes among African American ancestry samples for eQTL analyses.

We estimated latent factors using probabilistic estimation of expression residuals (PEER)[5] separately for each ancestry. Following PEER, we tested 12 million 1000 Genomes-imputed genetic variants for association with genes in *cis* (genes $\pm$1 MB of each variant) using Matrix eQTL[6]. eQTL models included age at death, sex, RIN, GAR, mitochondrial mapping rate, top 5 genotype principal components, and 4 PEER factors among European ancestry samples (N=104) and sex, RIN, top 5 genotype principal components, and 6 PEER factors as covariates among African American ancestry samples (N=94). Gene expression prediction model was also separately generated by ancestry using elastic net (as in PrediXcan), which resulted in 19,566 models for European ancestry and 16,526 models for African American ancestry.

Single Cell RNA-seq Data from Entire Mouse Nervous System

To identify smoking-relevant cell types, we made use of an existing gene expression dataset derived from 500,000 single cells from 19 regions in the mouse nervous system[7]. These single cells were then classified into 39 cell types. For each cell type, we created gene sets that consist of top 10% most highly expressed genes as "cell-type specific genes", which were used in enrichment analysis to identify cell types relevant for smoking behaviors[7].

3. Simulation Evaluation for TESLA

We conducted extensive simulations to evaluate the type I error and power of TESLA. We used real haplotype data to simulate genotype data that reflects realistic allele frequency and LD patterns. To assess power under the alternative hypothesis, for each simulated gene, we randomly selected a model from the PrediXcan database and used real eQTL weights as eQTL effects, which mediate the phenotypic effects in samples of European ancestry. For other ancestries, we considered different scenarios where the phenotypic effects and the set of causal variants are the same as European ancestry (i.e., homogeneous model) and where the causal variants and genetic effects are ancestry-specific (i.e., Eurasia, European only, or Admixed). We also examined the scenario

where the eQTL effects differ between ancestries (i.e., heterogeneous effect model), which may be due to different ancestries having different eQTL SNPs or effect heterogeneities. We varied the fraction of samples of European ancestry in the multi-ancestry studies to compare different methods. Type I errors and power were evaluated using 100 million replicates in each scenario under the Bonferroni threshold for testing up to 20,000 expressed genes ($\alpha = 2.5 \times 10^{-6}$). Details of different phenotypic effect models are shown in Suppl. Table 1 and **Supplementary Text**.

The type I error was controlled for all scenarios (Suppl. Table 2). In the scenarios with ancestry-specific effects, TESLA outperforms alternative methods in power. For example, in the Eurasia model, the phenotypic effects are only present in European and Asian samples. When 40% (20%) of the studies were of European (Asian) ancestry and the expression effect $c = .5$, the power for TESLA, FE-TWAS and EURO-TWAS were 93%, 88%, and 75%, respectively. If the phenotypic effect was only present in European samples, EURO-TWAS is expected to be the most powerful method, but its power was only slightly better than TESLA. In this case, FE-TWAS has much lower power as it ignores the heterogeneity of effect sizes (Suppl. Table 2). For the Admixed model, when the expression effect c was .25 and 40% of the cohorts were of European ancestry, TESLA was the most powerful method (58%). FE-TWAS does not incorporate effect heterogeneity, violates the proportionality condition, and has less power (55%). EURO-TWAS does not fully utilize non-European samples and was also underpowered (49%) (Suppl. Table 2).

In the presence of phenotypic effect heterogeneities, the power advantage for TESLA over FE-TWAS increases with the fraction of non-European samples. For example, in the European effects only model (*c=.25)*, when 80% of the samples were of European ancestry, the power for TESLA, FE-TWAS, and EURO-TWAS was 67%, 63%, and 68% respectively. As expected, EURO-TWAS was the most powerful method, but TESLA's power is comparable and FE-TWAS's power is only slightly lower. However, even when only 20% of the samples came from European ancestry, the power for the three methods was reduced to 32%, 11%, and 36%. The power for TESLA remains comparable to EURO-TWAS, the optimal method in this scenario, but FE-TWAS becomes severely underpowered.

The power comparison changed under the homogeneous phenotypic effect model: FE-TWAS was the most powerful method, yet the power for TESLA remained within 2% of FE-TWAS (Suppl. Table 2). The power of EURO-TWAS decreased dramatically when the fraction of European ancestry decreased. RE-TWAS consistently performed worse than FE-TWAS across all scenarios due to the conservativeness of the RE method in GWAS meta-analysis[8]. Some more advanced random effect meta-analysis methods do not produce effect size estimates, and hence cannot be used for TWAS.

Across all comparisons, TESLA was consistently the most powerful method or a close second, and the power advantage usually increased with the fraction of non-European samples, when phenotypic effect heterogeneity is present. On the other hand, FE-TWAS, RE-TWAS, and EURO-TWAS can be substantially underpowered in scenarios that do not favor their assumptions. Given the phenotypic model is unknown in practice and the expectation that human genetic studies will expand to include more non-European samples, TESLA established itself as a clear choice for TWAS.

4. **Extent of Phenotypic Effect Heterogeneity**

In TESLA, to model the phenotypic effects across studies, we fitted multiple meta-regression models with different numbers of PCs to capture the extent of phenotypic effect heterogeneities among ancestries. For each fitted model, we estimated phenotypic effects and performed TWAS separately. The meta-regression model that yielded the minimal p-value could inform the extent of heterogeneity of phenotypic effects across ancestries. We conduct the analyses using PrediXcan weights from GTEx sample of European ancestry as an example. Not

surprisingly, the phenotypic model with no PCs (equivalent to a fixed effects meta-analysis model) yielded minimal p-values for 77% of the genes, as a large proportion of phenotypic effects are expected to be homogeneous across ancestries. The first PC separates cohorts of African ancestry from the rest of the ancestries (Suppl. Figure 4). The model with 1 PC yielded minimal p-values for 11% of the genes, which indicated that these eQTL SNPs may show distinct effects in African ancestry samples. A small fraction of genes (12%) showed greater heterogeneity in phenotypic effects, as the minimal p-values were produced from models with more than 1 PC. (Suppl. Table 9, Suppl. Figure 5). As the phenotypic effects of eQTL SNPs vary between phenotypes, and different tissues have different sets of eQTL SNPs used in gene prediction models, the fractions of loci where models with 0/1/2/3 PCs yield minimal p-values differed slightly between phenotypes and tissue.

5. Fine-mapping of TESLA Identified Gene x Trait Associations

We performed fine mapping (see details in **Supplementary Text**) across the 4475 loci for all 48 tissues and four tobacco use phenotypes. Among these loci, 77% were fine mapped to a single gene in the 90%-credible set (Suppl. Table 10). Our results point to novel target genes with biological relevance, pleiotropic effects on neuropsychiatric traits, and tissue-specific effects.

First, fine-mapping identified potential causal genes with biological relevance to tobacco use. For example, in the hypothalamus, a brain region that regulates body homeostasis, stress hormone release, and circadian rhythm, *HEY1* (Hes Related Family BHLH Transcription Factor with YRPW Motif 1) was identified. Overexpression of *HEY1* in hypothalamus is associated with an increase in CigDay (TESLA max Z-score 4.83, multi-tissue two-sided p-value 2.4×10^{-6}, Posterior inclusion probability (PIP)=1) (Suppl. Figure 6a). This gene is a target for the Notch signaling pathway, an important regulator of neuronal development and proper network development in the brain[9]. It has also been identified as a potential candidate gene for the regulation of the dopamine transporter (*SLC6A3*) gene [10]. The TESLA analysis using African ancestry eQTL data from the LIBD nucleus accumbens dataset identified *DTX4* to be significantly associated with CigDay (two-sided $p=1.8\times10^{-7}$). Ubiquitylation of Notch1 by the E3 ubiquitin ligase *DTX4* is known to promote the internalization of Notch1 in response to ligand binding[11], which also highlighted the impact of the Notch signaling pathway. *DTX4* was not significant in TESLA analysis using the European ancestry eQTL datasets from GTEx.

Fine-mapping further identified the gene *ASIP* as a potential causal gene, where an increased level of genetically regulated gene expression level in brain cortex leads to decreased CigDay (TESLA Z-score statistic -4.63 with two-sided multi-tissue p-value 4.5×10^{-8}, PIP=1). (Suppl. Figure 6b). *ASIP* encodes the agouti-signaling protein, which acts as an antagonist to melanocortin receptors (MCR), similarly to agouti-related protein. The *MC1R* gene is typically associated with skin pigmentation, but alterations in the gene have also been associated with modulated pain sensitivity[12]. The *MC4R* gene has been associated with several psychological diseases such as depression and anxiety[13,14]. This receptor may impact hypothalamic-pituitary-adrenal (HPA) stress axis functionality[15]. Antagonists for the receptor have even been suggested for preventing or treating post-traumatic stress disorder (PTSD)[16]. Nicotine has also been shown to change the expression pattern of *MC4R* in the brain[17].

Additionally, fine-mapping identified *PTPRD* (protein tyrosine phosphatase receptor type D) that has been associated with cocaine addiction. Specifically, *PTPRD* was identified, where an increase in the genetically regulated gene expression level in amygdala leads to decreased risk of smoking initiation (TESLA Z-score statistic -4.0 with two-sided multi-tissue p-value 4.8×10^{-7}, PIP=.94) (Suppl. Figure 6c). Previous research has shown that *PTPRD* knock-out mice have reduced overall use of cocaine and reduced conditioned place preference for the drug[18].

6. Correlation between TESLA Statistics

In TESLA, we model the genetic effect heterogeneity using meta-regression models. Specifically, the meta-regression model with L PCs takes the form:

$$M^{[L]}: b_{\cdot k} = \sum_{l}^{L} X_{kl}\gamma_{l\cdot}^{[L]} + \epsilon_{\cdot k}$$

The regression coefficients can be estimated based on weighted least square method:

$$\hat{\gamma}_{\cdot j}^{[L]} = \left(\boldsymbol{X}^{[L]\prime}\boldsymbol{\Omega}_{j}\boldsymbol{X}^{[L]}\right)^{-1}\boldsymbol{X}^{[L]\prime}\boldsymbol{\Omega}_{j}\boldsymbol{b}_{j\cdot}$$

With $\hat{\gamma}_{\cdot j}^{[L]}$, we can estimate the phenotypic effect in the ancestry of the eQTL dataset. We denote the allele frequency PCs of the eQTL dataset as $\tilde{X}^{[L]}$ and the estimated phenotypic effect equals to

$$\hat{b}_{j}^{[L]} = \tilde{X}^{[L]}\hat{\gamma}_{\cdot j}^{[L]}$$

For each model $M^{[L]}$, we construct TWAS statistics

$$U_{TWAS}^{[L]} = \sum_{j=1}^{M} w_j \hat{b}_j^{[L]} / \hat{s}_j^{[L]}$$

With variance

$$V_{TWAS}^{[L]} = \mathbf{w}'\boldsymbol{\Sigma}_{\mathbf{b}}\mathbf{w}$$

Finally, we combine the results based upon different models using minimal p-value statistic.

In order to evaluate the statistical significance for minimal p-value statistic, we need to calculate the correlations between TWAS statistics $U_{TWAS}^{[L]}\big/\sqrt{V_{TWAS}^{[L]}}$, $L = 0,1,2$ and 3. As the calculated TWAS statistics are functions of phenotypic effect estimates, a critical step is to estimate the covariance between $\hat{b}_{j_1}^{[L_1]}$ and $\hat{b}_{j_2}^{[L_2]}$. It is straightforward to verify that

$$\mathrm{cov}\left(\hat{b}_{j_1}^{[L_1]}, \hat{b}_{j_2}^{[L_2]}\right) = \left(\boldsymbol{X}^{[L_1]\prime}\boldsymbol{\Omega}_{j_1}\boldsymbol{X}^{[L_1]}\right)^{-1}\boldsymbol{X}^{[L_1]\prime}\boldsymbol{\Omega}_{j_1}\mathrm{cov}(\boldsymbol{b}_{j_1\cdot}, \boldsymbol{b}_{j_2\cdot})\boldsymbol{\Omega}_{j_2}\boldsymbol{X}^{[L_2]}\left(\boldsymbol{X}^{[L_2]\prime}\boldsymbol{\Omega}_{j_2}\boldsymbol{X}^{[L_2]}\right)^{-1}$$

So we only need to find out $\mathrm{cov}(\boldsymbol{b}_{j_1\cdot}, \boldsymbol{b}_{j_2\cdot})$, which is a $K \times K$ diagonal matrix

$$\mathrm{cov}(\boldsymbol{b}_{j_1\cdot}, \boldsymbol{b}_{j_2\cdot}) = diag\left(\mathrm{cov}(b_{j_1,1}, b_{j_2,1}), \ldots, \mathrm{cov}(b_{j_1,K}, b_{j_2,K})\right)$$

The correlation between phenotypic effects can be approximated by the LD coefficients. Given that the ancestry of participating cohorts may differ, it is important to choose an appropriate LD reference panel for each cohort based upon its ancestry. In our analysis, we used TOPMed sequence data as reference panel. For each cohort, we choose individuals of the same ancestry from TOPMed for use as reference panel and estimate correlations between phenotypic effect estimates. We assumed and verified that the cohorts are independent of each other.

We denote the covariance matrix between $\hat{b}_{j_1}^{[L_1]}$'s and $\hat{b}_{j_2}^{[L_2]}$'s as

$$\mathrm{COV}(\hat{\boldsymbol{b}}_{\cdot}^{[L_1]}, \hat{\boldsymbol{b}}_{\cdot}^{[L_2]}) = \mathrm{cov}\left(\hat{b}_{j_1}^{[L_1]}, \hat{b}_{j_2}^{[L_2]}\right)_{1\le j_1, j_2 \le M}$$

As the TWAS statistics are linear combinations of the phenotypic effect estimates, their covariance is straightforward to calculate, i.e.,

$$\mathrm{cov}\left(U_{TWAS}^{[L_1]}, U_{TWAS}^{[L_2]}\right) = \left(w_1/\hat{s}_1^{[L_1]}, \ldots, w_M/\hat{s}_M^{[L_1]}\right)' \mathrm{cov}(\hat{b}_{\cdot}^{[L_1]}, \hat{b}_{\cdot}^{[L_2]})\left(w_1/\hat{s}_1^{[L_2]}, \ldots, w_M/\hat{s}_M^{[L_2]}\right)$$

With the covariance between TWAS statistics, the minimal p-values can be evaluated based on multivariate normal distribution functions.

7. Equivalence of FE-TWAS and meta-analysis of TWAS statistics from participating studies.

FE-TWAS is a special case of TESLA where no allele frequency PC is included. In this section, we will establish theoretically that when eQTL weights used in different studies are the same, FE-TWAS is equivalent to meta-TWAS, which is to conduct TWAS for each participating cohort and then combine the TWAS statistics across studies using inverse-variance weighted meta-analysis.

The equivalence is intuitively clear: meta-TWAS performs TWAS within each ancestry/study, aggregates information across variant sites, and then conducts meta-analysis across studies/ancestries. On the other hand, FE-TWAS first aggregates information across studies/ancestries and then across variant sites. The two methods only differ in the order of data integration, i.e., either aggregating over variant sites first (meta-TWAS) or over studies first (FE-TWAS). As summation is commutative, exchanging the order of summation (across variant sites vs. across studies/ancestries) yields identical results.

Specifically, FE-TWAS method conducts TWAS using fixed-effect GWAS meta-analysis results, i.e.,

$$Z_j^{FE} = \frac{1}{C}\sum_k b_{jk} s_{jk}^{-2}$$

where C is a normalizing constant:

$$C = \left(\sum_k s_{jk}^{-2}\right)^{1/2}$$

The FE-TWAS statistic is given by

$$T^{FE-TWAS} = \left(\sum_j w_j Z_j^{FE}\right)^2 \Bigg/ var\left(\left(\sum_j w_j Z_j^{FE}\right)\right) = \left(\sum_j w_j \sum_k b_{jk} s_{jk}^{-2}\right)^2 \Bigg/ C^2 var\left(\left(\sum_j w_j Z_j^{FE}\right)\right)$$

It is easy to verify that

$$T^{FE-TWAS} = \frac{(U^{FE-TWAS})^2}{V^{FE-TWAS}}$$

where

$$U^{FE-TWAS} = \sum_j w_j \sum_k b_{jk} s_{jk}^{-2}$$

and

$$V^{FE-TWAS} = var(U^{FE-TWAS}) = C^2 var\left(\left(\sum_j w_j Z_j^{FE}\right)\right)$$

$V^{FE-TWAS}$ can be calculated based on cohort specific LD panels, as we describe in the previous section.

On the other hand, the meta-TWAS first performs TWAS in each ancestry/study and then combines the results using inverse-variance weighted meta-analysis. In study/ancestry k, TWAS analyzes imputed gene expression in linear models. Without loss of generality, we assume that the trait residuals (after adjusting for non-genetic covariates) are standardized to have mean of 0 and variance of 1, to simplify notations.

The regression model for TWAS takes the form of

$$Y_{ik} = \left(\sum_j w_j G_{ijk}\right)\beta + \epsilon_{ik}$$

where

- Y_{ijk} is phenotype residual for individual i in study/ancestry k
- G_{ijk} is the genotype for individual i at variant j in study k.
- ϵ_{ik} is residual for the regression model.

The least square estimates for β is given by

$$b_k^{TWAS} = \left(\sum_i \left(\sum_j w_j G_{ijk} \right)^2 \right)^{-1} \sum_i \left(\sum_j w_j G_{ijk} \right) Y_{ik}$$

We further define:

$$U_k^{TWAS} = \sum_i \left(\sum_j w_j G_{ijk} \right) Y_{ik} = \sum_j w_j \left(\sum_i G_{ijk} Y_{ik} \right) = \sum_j w_j b_{jk} s_{jk}^{-2}$$

$$V_k^{TWAS} = \sum_i \left(\sum_j w_j G_{ijk} \right)^2 = var\left(U_k^{TWAS}\right)$$

It is easy to verify that

$$b_k^{TWAS} = U_k^{TWAS} / V_k^{TWAS}$$

The meta-TWAS statistic is given by inverse-variance weighted meta-analysis of b_k^{TWAS}, with weights being $1/V_k^{TWAS}$, i.e.,

$$T^{meta-TWAS} = \left(\sum_k U_k^{TWAS} \right)^2 \left(\sum_k V_k^{TWAS} \right)^{-1}$$

It is important to note that

$$\sum_k U_k^{TWAS} = \sum_k \sum_j w_j b_{jk} s_{jk}^{-2} = \sum_j w_j \sum_k b_{jk} s_{jk}^{-2} = U^{FE-TWAS}$$

which establishes the equivalence of meta-TWAS and FE-TWAS.

8. Simulation Study:

We conducted extensive simulations to evaluate the type I error and power of TESLA. We used real haplotype data to simulate genotype data that reflects realistic allele frequency and LD patterns. We considered a number of scenarios where the phenotypic effects and the set of causal variants differ between ancestries and where the phenotypic effects and causal variants remain the same. We also varied the fraction of samples of European ancestry in the multi-ancestry studies.

More specifically, we first simulated a meta-analysis of 20 studies. Genotypes for each gene were simulated based upon pairing randomly selected haplotypes from the TOPMed for the given ancestry. We varied the fraction of European studies in the meta-analysis between 20% to 80%. Half of the non-European cohorts were generated using haplotypes from African American ancestry and the other half were simulated using haplotypes from East Asian ancestry. For each replicate, a gene was randomly chosen from the PrediXcan database of GTEx whole blood tissue. The phenotypic effect β_j was simulated using the weights w_j from the chosen gene and gene expression effect on phenotypes (c), i.e., $\beta_j = w_j c$. We varied c among a set of plausible values (i.e., 0.25, 0.33, or 0.5). We considered scenarios with different phenotypic effects in non-European populations, including the scenario where the phenotypic effects are homogenous across ancestries and where the phenotypic effects were only present in a subset of ancestries. We also considered a scenario of admixed effects, where only the allele of European descent has non-zero phenotypic effect (in samples of European ancestry and samples of African American ancestry). A summary for the simulation models can be found in (Suppl. Table 1).

9. Enrichment Analysis

Here we describe enrichment analyses using TESLA hits as well as the application to evaluate the drug target enrichment for drug repurposing. As a comparison, we also conducted enrichment analysis based on GWAS hits using MAGMA.

Quantifying Pathway Enrichment of TESLA Hits

Gene-level association results, when combined with pathway information, can be used to prioritize key pathways for tobacco use phenotypes. We used the same weighted regression approach[19] as MAGMA to quantify the enrichment of target genes in each pathway, which we call eTESLA. Contrary to MAGMA, which calculates a gene-level statistic from single SNP p-values in GWAS, the eTESLA statistic is based upon TESLA p-values from each tissue. To implement weighted regression, we need to calculate correlation between TESLA statistics of different genes using a Monte Carlo algorithm.

As the first step of eTESLA, we converted the TESLA p-values to Z-scores using inverse normal transformation. We denote the converted vector of Z-scores as:

$$\boldsymbol{Z} = \left(Z_1, \dots, Z_{N_{gene}}\right)^T$$

Where Z_g is the Z-score for gene g. We also encoded the membership of each gene in different pathways using an indicator matrix, i.e.,

$$\boldsymbol{C} = \left(C_{gp}\right)_{1 \le g \le N_{gene}, 1 \le p \le N_{pathway}}$$

Here, C_{gp} equals to 1 if gene g belongs to pathway p, and 0 otherwise. A weighted regression analysis was then conducted by regressing the gene-level Z-score over the pathway membership covariates, i.e.:

$$\boldsymbol{Z} = \boldsymbol{C\alpha} + \boldsymbol{\epsilon}$$

The model can be fitted using weighted least square, i.e.,

$$\widehat{\boldsymbol{\alpha}} = (\mathbf{C}'\boldsymbol{\Sigma}_{\mathbf{Z}}\mathbf{C})^{-1}\mathbf{C}'\boldsymbol{\Sigma}_{\mathbf{Z}}Z$$

$\boldsymbol{\Sigma}_{\mathbf{Z}}$ is the covariance matrix between Z-score statistics converted from TESLA p-values, which we will calculate using a Monte Carlo algorithm as described below.

Monte Carlo Algorithm for Calculating $\boldsymbol{\Sigma}_{\mathbf{Z}}$

For each pair of genes (for which we denote as gene 1 and gene 2, with variants $j_1 = 1, \dots, M_1$ and $j_2 = M_1 + 1, \dots, M_1 + M_2$). We repeat steps 1-3 10,000 times. In iteration a,

Step 1: We simulate phenotypic effects $b_1^{[0]}, \dots, b_1^{[3]}, \dots, b_{M_1+M_2}^{[0]}, \dots, b_{M_1+M_2}^{[3]} \sim MVN\left(0, \Sigma_{b_1,b_2}\right)$, where Σ_{b_1,b_2} is the correlation matrix between the estimated phenotypic effects (as detailed in Supplementary Text).

Step 2: For each simulated vector of the phenotypic effects, we calculate the TESLA statistic using different numbers of PCs and calculate the p-values for the TESLA statistic of genes 1 and 2 respectively.

Step 3: We convert the p-values for genes 1 and 2 to Z statistics $Z_{1,a}$ and $Z_{2,a}$.

The covariance between the TESLA p-value converted Z-scores is given by $\frac{1}{10000}\sum_a (Z_{1a} - \bar{Z}_1)(Z_{2a} - \bar{Z}_2)$, where $\bar{Z}_1 = \frac{1}{10000}\sum_a Z_{1a}$, and $\bar{Z}_2 = \frac{1}{10000}\sum_a Z_{2a}$.

Go enrichment and semantic similarity analysis.

GO items gene sets were retrieved from The Molecular Signatures Database (MSigDB) ontology gene set collection (c5), which consists of a comprehensive catalog of known disease-associated proteins[20,21]. To reduce the redundancy of GO items for each trait and tissue pair, we leveraged REVIGO[22] to calculate the semantic similarity measures between GO terms and then clustered similar GO terms. REVIGO uses a simple clustering algorithm to summarize a list of GO terms using their sematic similarity measures. It relies on pre-computed information content for GO terms. This method could reduce the redundant and tangled raw GO analysis results by choosing a representative subset of the terms, which facilitates visualization and interpretation. We also compared the results with other tools (e.g. simplifyEnrichment[23]) that use information theoretic similarity[24], to

verify the robustness of the results. The REVIGO results are visualized by using CirGO[25] to deliver more comprehensive and intuitive information. As a sensitivity analysis on the impact of the pathway database used, we also performed enrichment analysis using KEGG[26], Reactome[27], and wikiPathways[28] following the same pipeline. All reported p-values are two-sided.

Drugbank sets enrichment Analysis for Drug Repurposing Analysis

We leveraged enrichment analysis to prioritize key drug pathways that are enriched with TESLA hits, which were then used to identify putative drugs that may be repurposed for smoking cessation treatment. We made use of DrugBank[29], a publicly available database that contains >10,000 FDA-approved drugs along with data on ~5000 unique drug targets, to compile gene sets that consist of target genes for each drug. We removed drugs with less than two drug target genes, as enrichment analysis cannot be evaluated for gene sets with only one gene[30,31]. Resulting gene sets consist of 1642 drugs for enrichment analysis. All reported p-values are two-sided.

To further explore the relationships between original drug indications and different underlying molecular pathways, we classified all the identified drugs based on indications and molecular mechanism of action, respectively.

First, we manually reviewed and curated all the significant drugs' indications and major target gene groups. We grouped drug indications into 15 groups including:

1. Pains.
2. Mental Disorder: Anxiety, ADHD, Schizophrenia, Insomnia, PTSD, and Panic.
3. Nervous system: PD, AD, MS, Migraine, Epilepsy, and seizures.
4. Sedative/Hypnotic.
5. Hypertensive disorder.
6. Carcinoma/Cancers.
7. Anesthetic/Muscle relaxants.
8. Rheumatoid arthritis.
9 Respiratory system: Asthma.
10. Obesity/Diabetes.
11. Infections/Antibiotics.
12. AUD/Abuse.
13. Digestive system disease: Bowel disorder, Ulcerative colitis, Inflammatory Bowel Disease, and Psoriasis.
14. Cardiovascular disease.
15. Others.

We also grouped drug's mechanisms of action into four different categories, including
1. Nicotine metabolism genes.
2. nAChR (Nicotinic receptor subunit genes).
3. Dopamine and other relevant neurotransmitter systems.
4. γ-aminobutyric acid (GABA)ergic signaling system.

MAGMA Enrichment Analysis for Identifying Relevant Tissues and Cell Types

In order to pinpoint tissues or cell types for tobacco use phenotypes, MAGMA (v1.08) was used to assess enrichment of GWAS signals in the top 10% highly expressed genes in each tissue or cell type. As MAGMA was developed for samples from a single ancestry, we only used GWAS fixed effect meta-analysis of samples with

European ancestry. We conducted the analysis using default parameters, and calculated p-values for enrichment as well as false discovery rates for each gene set. All reported p-values are two-sided.

10. Fine Mapping TESLA Results

TESLA statistics adaptively combine the p-values from each sub-model with different number of principal components, in order to accommodate different extent of phenotypic effect heterogeneities between ancestries. Here, we performed fine-mapping of TWAS hits by extending existing methods based upon Gaussian Copula. We first transformed two-sided TWAS p-values of each gene to Z-score statistics [i.e., $Z = 1 - \Phi^{-1}(p)$, where Φ is the cumulative distribution function for standard normal random variables], then estimated correlation between converted Z-scores using the Monte Carlo approach as in enrichment analysis, and finally calculated Bayes factors and posterior inclusion probabilities to quantify the probability that each gene was causal. This is conceptually similar to other GWAS/TWAS fine-mapping methods using approximate Bayes factors[32]. However, through working with p-values, this method allows us to work with a broader class of statistical methods including the ones based upon combined p-values.

Specifically, for fine-mapping, we defined each locus being a 1 Mb window surrounding a significant TESLA signal. The TESLA p-values are denoted by $\tilde{p}_1, \dots, \tilde{p}_g$ and the single SNP p-values in the gene region that are not used in gene expression models are denoted as $p_1, \dots, p_v$. We converted these p-values to Z-score statistics using inverse normal transformation, which we denoted as $\tilde{T}_1, \dots, \tilde{T}_g, T_1, \dots, T_v$ (i.e., $T = \Phi^{-1}(1-p)$, where Φ is the cumulative distribution function for standard normal random variable). Next, we estimated correlations between converted statistics. Under the null hypothesis, the single variant association statistics follow multivariate normal distribution, with correlation matrix equal to the LD coefficients. To calculate the correlation between statistics $\tilde{T}_1, \dots, \tilde{T}_g, T_1, \dots, T_v$, we employ a Monte Carlo approach as described for eTESLA.

Similar to several other fine mapping methods[33,34], we took an iterative approach which allows us to fine map the top signal in the locus first and then for secondary signals using conditional association results. We calculated the approximate Bayes factor for the primary signal, i.e., gene g_0 using the approximate Bayes factor:

$$ABF(g_0) = \frac{1}{\sqrt{\tau^2 + 1}} \exp\left(\frac{\tau^2 Z_{g_0}^2}{2(1+\tau^2)}\right)$$

with $\tau = 0.1$ being the standard deviation of the prior on the effect sizes, following Wakefield[32].

The PIP for gene g_0 can be calculated by

$$PIP(g_0) = \frac{ABF(g_0)}{\sum_l ABF(l)}$$

When multiple association signals are present, conditional analysis of Z-scores is performed conditioning on the top gene/variants in each locus. The fine-mapping for secondary association signals is conducted using conditional Z-scores.

11. Funding acknowledgement for participating studies.

Molecular data for the Trans-Omics in Precision Medicine (TOPMed) program was supported by the National Heart, Lung and Blood Institute (NHLBI). Genome Sequencing for "NHLBI TOPMed: MESA AA_CAC" (phs001416.v1.p1) was performed at Broad Institute Genomics Platform (HHSN268201500014C). Genome Sequencing for "NHLBI TOPMed: Amish" (phs000956.v1.p1) was performed at Broad Institute Genomics Platform (3R01HL121007-01S1). Genome Sequencing for "NHLBI TOPMed: ARIC" (phs001211.v1.p1) was performed at Baylor College of Medicine Human Genome Sequencing Center (3U54HG003273-12S2 / HHSN268201500015C). Genome Sequencing for "NHLBI TOPMed: BAGS" (phs001143.v1.p1) was performed at

Illumina (3R01HL104608-04S1). Genome Sequencing for "NHLBI TOPMed: EOCOPD" (phs000946.v1.p1) was performed at NORTHWEST GENOMICS CENTER (3R01HL089856-08S1). Genome Sequencing for "NHLBI TOPMed: CFS" (phs000954.v1.p1) was performed at NORTHWEST GENOMICS CENTER (3R01HL098433-05S1, HHSN268201600032I). Genome Sequencing for "NHLBI TOPMed: CHS" (phs001368.v1.p1) was performed at Baylor College of Medicine Human Genome Sequencing Center (HHSN268201600033I). Genome Sequencing for "NHLBI TOPMed: COPDGene" (phs000951.v1.p1) was performed at Broad Institute Genomics Platform (HHSN268201500014C) and NORTHWEST GENOMICS CENTER (3R01HL089856-08S1). Genome Sequencing for "NHLBI TOPMed: CRA" (phs000988.v1.p1) was performed at NORTHWEST GENOMICS CENTER (3R37HL066289-13S1, HHSN268201600032I). Genome Sequencing for "NHLBI TOPMed: ECLIPSE" (phs001472.v1.p1) was performed at MCDONNELL GENOME INSTITUTE (HHSN268201600037I). Genome Sequencing for "NHLBI TOPMed: FHS" (phs000974.v1.p1) was performed at Broad Institute Genomics Platform (3U54HG003067-12S2). Genome Sequencing for "NHLBI TOPMed: GeneSTAR" (phs001218.v1.p1) was performed at Psomagen (3R01HL112064-04S1), Illumina (R01HL112064), and Broad Genomics (HHSN268201500014C). Genome Sequencing for "NHLBI TOPMed: GENOA" (phs001345.v1.p1) was performed at NORTHWEST GENOMICS CENTER (3R01HL055673-18S). Genome Sequencing for "NHLBI TOPMed: GenSalt" (phs001217.v1.p1) was performed at Baylor College of Medicine Human Genome Sequencing Center (HHSN268201500015C). Genome Sequencing for "NHLBI TOPMed: GOLDN" (phs001395.v1.p1) was performed at NORTHWEST GENOMICS CENTER (3R01HL104135-04S1). Genome Sequencing for "NHLBI TOPMed: HCHS_SOL" (phs001395.v1.p1) was performed at Baylor College of Medicine Human Genome Sequencing Center (HHSN268201600033I). Genome Sequencing for "NHLBI TOPMed: HVH" (phs000993.v1.p1) was performed at Broad Institute Genomics Platform (3R01HL092577-06S1). Genome Sequencing for "NHLBI TOPMed: GENOA" (phs001345.v1.p1) was performed at NORTHWEST GENOMICS CENTER (3R01HL055673-18S1). Genome Sequencing for "NHLBI TOPMed: IPF" (phs001607.v1.p1) was performed at MCDONNELL GENOME INSTITUTE (HHSN268201600037I). Genome Sequencing for "NHLBI TOPMed: JHS" (phs000964.v1.p1) was performed at NORTHWEST GENOMICS CENTER (HHSN268201100037C). Genome Sequencing for "NHLBI TOPMed: MESA" (phs001416.v1.p1) was performed at Broad Institute Genomics Platform (3U54HG003067-13S1). Genome Sequencing for "NHLBI TOPMed: OMG_SCD" (phs001608.v1.p1) was performed at Baylor College of Medicine Human Genome Sequencing Center (HHSN268201500015C). Genome Sequencing for "NHLBI TOPMed: SAFS" (phs001215.v1.p1) was performed at Illumina (3R01HL113323-03S1). Genome Sequencing for "NHLBI TOPMed: SARP" (phs001446.v1.p1) was performed at New York Genome Center Genomics (HHSN268201500016C). Genome Sequencing for "NHLBI TOPMed: Samoan" (phs000972.v1.p1) was performed at NORTHWEST GENOMICS CENTER (HHSN268201100037C). Genome sequencing for “NHLBI TOPMed: CAMP” (phs001726.v1.p1) was performed at the Northwest Genomics Center (HHSN268201600032I). Genome sequencing for “NHLBI TOPMed: THRV” (phs001387.v1.p1) was performed at the Baylor College of Medicine Human Genome Sequencing Center (3R01HL111249-04S1, HHSN26820150015C). Genome Sequencing for "NHLBI TOPMed: Mayo_VTE" (phs001402.v1.p1) was performed at Baylor College of Medicine Human Genome Sequencing Center (3U54HG003273-12S2 / HHSN268201500015C). Genome Sequencing for "NHLBI TOPMed: WGHS" (phs001040.v1.p1) was performed at Broad Institute Genomics Platform (3R01HL092577-06S1). Genome Sequencing for "NHLBI TOPMed: WHI" (phs001237.v1.p1) was performed at Broad Institute Genomics Platform (HHSN268201500014C). Core support including centralized genomic read mapping and genotype calling, along with variant quality metrics and filtering were provided by the TOPMed Informatics Research Center (3R01HL-117626-02S1; contract HHSN268201800002I). Core support including phenotype harmonization, data management, sample-identity QC, and general program coordination were provided by the TOPMed Data Coordinating Center (R01HL-120393; U01HL-120393; contract HHSN268201800001I). We gratefully acknowledge the studies and participants who provided biological samples and data for TOPMed. The LIBD data was supported by the National Institute on Drug Abuse R01 DA042090 (PI: DBH).”.

12. **The Information of Participating Cohorts in GSCAN and TOPMed.**

23andMe, Inc. (23andMe)

23andMe research participants provided informed consent to take part in this research under a protocol approved by the AAHRPP-accredited institutional review board, Ethical and Independent Review Services. We would like to thank the research participants and employees of 23andMe for making this work possible.

ALSPAC (Avon Longitudinal Study of Parents and Children)

We are extremely grateful to all the families who took part in this study, the midwives for their help in recruiting them, and the whole ALSPAC team, which includes interviewers, computer and laboratory technicians, clerical workers, research scientists, volunteers, managers, receptionists and nurses. The UK Medical Research Council and Wellcome (Grant ref: 102215/2/13/2) and the University of Bristol provide core support for ALSPAC. This research was specifically funded by the UK Medical Research Council and the University of Bristol (MC_UU_12013/1, MC_UU_00011/7). LZ is supported by the UK Medical Research Council (G0902144). AET and MRM are members of the UK Centre for Tobacco Control Studies, a UKCRC Public Health Research: Centre of Excellence. Funding from British Heart Foundation, Cancer Research UK, Economic and Social Research Council, Medical Research Council, and the National Institute for Health Research, under the auspices of the UK Clinical Research Collaboration, is gratefully acknowledged. GWAS data was generated by Sample Logistics and Genotyping Facilities at Wellcome Sanger Institute and LabCorp (Laboratory Corporation of America) using support from 23andMe. This publication is the work of the GWAS and Sequencing Consortium of Alcohol and Nicotine use (GSCAN), which serves as guarantors for the contents of this paper.

Descriptions of the ALSPAC cohort can be found in the two following articles: (1) Boyd A, Golding J, Macleod J, Lawlor DA, Fraser A, Henderson J, Molloy L, Ness A, Ring S, Davey Smith G. Cohort Profile: The 'Children of the 90s'; the index offspring of The Avon Longitudinal Study of Parents and Children (ALSPAC). International Journal of Epidemiology 2013; 42:111-127; (2) Fraser A, Macdonald-Wallis C, Tilling K, Boyd A, Golding J, Davey Smith G, Henderson J, Macleod J, Molloy L, Ness A, Ring S, Nelson SM, Lawlor DA. Cohort Profile: The Avon Longitudinal Study of Parents and Children: ALSPAC mothers cohort. International Journal of Epidemiology 2013; 42:97- 110. Study data for individuals 22 years and older were collected and managed using REDCap electronic data capture tools hosted at the University of Bristol. REDCap (Research Electronic Data Capture) is a secure, web-based application designed to support data capture for research studies, providing 1) an intuitive interface for validated data entry; 2) audit trails for tracking data manipulation and export procedures; 3) automated export procedures for seamless data downloads to common statistical packages; and 4) procedures for importing data from external sources. The tool is described in detail in the following article: Paul A. Harris, Robert Taylor, Robert Thielke, Jonathon Payne, Nathaniel Gonzalez, Jose G. Conde, Research electronic data capture (REDCap) – A metadata-driven methodology and workflow process for providing translational research informatics support, Journal of Biomedical Informatics 2009; 42(2):377-381.

Luisa Zuccolo was partially funded by the UK Medical Research Council through grants G0902144 and MC_UU_12013/1.

Please note that the ALSPAC study website contains details of all the data that is available through a fully searchable data dictionary available here: http://www.bris.ac.uk/alspac/researchers/data-access/data-dictionary/. Ethical approval for the study was obtained from the ALSPAC Ethics and Law Committee and the Local Research Ethics Committees. Details on the ethics committee/institutional review board that approved aspects of the study can be found here: http://www.bristol.ac.uk/alspac/researchers/research-ethics/. For more information about this dataset, see http://www.bristol.ac.uk/alspac/.

Amish (Genetics of Cardiometabolic Health in the Amish)

The Amish Complex Disease Research Program includes a set of large community-based studies focused largely on cardiometabolic health carried out in the Old Order Amish (OOA) community of Lancaster, Pennsylvania (http://medschool.umaryland.edu/endocrinology/amish/research-program.asp). The OOA population of Lancaster County, PA immigrated to the Colonies from Western Europe in the early 1700's. There are now over 30,000 OOA individuals in the Lancaster area, nearly all of whom can trace their ancestry back 12-14 generations to approximately 700 founders. Investigators at the University of Maryland School of Medicine have been studying the genetic determinants of cardiometabolic health in this population since 1993. To date, over 7,000 Amish adults have participated in one or more of our studies.

Due to their ancestral history, the OOA are enriched for rare exonic variants that arose in the population from a single founder (or small number of founders) and propagated through genetic drift. Many of these variants have large effect sizes and identifying them can lead to new biological insights about health and disease. The parent study for this WGS project provides one (of multiple) examples. In our parent study, we identified through a genome-wide association analysis a haplotype that was highly enriched in the OOA that is associated with very high LDL-cholesterol levels. At the present time, the identity of the causative SNP – and even the implicated gene – is not known because the associated haplotype contains numerous genes, none of which are obvious lipid candidate genes. A major goal of the WGS that will be obtained through the NHLBI TOPMed Consortium will be to identify functional variants that underlie some of the large effect associations observed in this unique population.

The TOPMed component of the Amish Research Program was supported by NIH grants R01 HL121007, U01 HL072515, and R01 AG18728. Email Rhea Cosentino (rcosenti@som.umaryland.edu) for additional input.

ARIC (Atherosclerosis Risk in Communities)

The Cohort Component began in 1987, and each ARIC field center randomly selected and recruited a cohort sample of approximately 4,000 individuals aged 45-64 from a defined population in their community, to receive extensive examinations, including medical, social, and demographic data. Follow-up also occurs semi-annually, by telephone, to maintain contact and to assess health status of the cohort.

In the Community Surveillance Component, the four communities are investigated to determine the long term trends in hospitalized myocardial infarction (MI) and coronary heart disease (CHD) deaths in approximately 470,000 men and women aged 35-84 years.

Objectives of the study includes: (1) Examine the ARIC cohort to characterize heart failure stages in the community, identify genetic and environmental factors leading to ventricular dysfunction and vascular stiffness, and assess longitudinal changes in pulmonary function and identify determinants of its decline. (2) Cohort follow-up for cardiovascular events, including CHD, heart failure, stroke, and atrial fibrillation; and for the study of risk factors related to progression of subclinical to clinical CVD. (3) Enhance the ARIC cohort study with cardiovascular outcomes research to assess quality and outcomes of medical care for heart failure and heart failure risk factors. (4) Community surveillance to monitor long-term trends in hospitalized MI, CHD deaths, and heart failure (inpatient and outpatient). (5) Provide a platform for ancillary studies, training for new investigators, and data sharing.

The Atherosclerosis Risk in Communities study has been funded in whole or in part with Federal funds from the National Heart, Lung, and Blood Institute, National Institutes of Health, Department of Health and Human Services, under Contract nos. (HHSN268201700001I, HHSN268201700002I, HHSN268201700003I, HHSN268201700005I, HHSN268201700004I). The authors thank the staff and participants of the ARIC study for their important contributions.

BAGS (Barbados Asthma Genetics Study)
Epidemiologic studies of asthma have been underway in Barbados since 1991, when PI Barnes reported a relationship between modernization of the domestic environment in Barbados and increased risk of asthma. The baseline prevalence of asthma in Barbados is high (~20%), and from admixture analyses, we have determined that the proportion of African ancestry among Barbadian founders is similar to U.S. African Americans, rendering this a unique population to disentangle the genetic basis for asthma disparities among African ancestry populations in general. The primary outcome measure is asthma, and the approach for characterizing asthma in the Barbados population is based on the validated Respiratory Health Questionnaire (RHQ) designed from the 1978 American Thoracic Society questionnaire. Additional phenotype data include lung function measures, asthma severity, total serum IgE, and serum levels of various cytokines. In 1993, the Barbados Asthma Genetics Study (BAGS) was initiated on nuclear and extended asthmatic families who self-reported as African Caribbean, resulting in the first evidence for linkage for asthma and tIgE in an African-ancestry population, and the development of novel family-based methods. Recruitment into the BAGS program was enhanced through its involvement in the international Genetics of Asthma International Network (1999-2001) and the current sample of >1300 participants continues to grow through the efforts of collaborators and nursing staff at the Chronic Disease Research Centre in Barbados. Pediatric probands were recruited through referrals at local polyclinics or the Accident and Emergency Department at the Queen Elizabeth Hospital, and their nuclear and extended family members were subsequently recruited. All subjects gave verbal and written consent as approved by the Johns Hopkins Institutional Review Board (IRB) and the Barbados Ministry of Health.

In 2007 we performed a genome-wide association study (GWAS) on 655,352 SNPs using the Illumina Infinium™ II HumanHap650Y BeadChip v.1.0 (Illumina Inc.) on a subset of 1,000 Barbados participants. This represented the first GWAS of asthma focusing exclusively on populations of African ancestry, and data from this study also contributed to the NHLBI-supported EVE Consortium. BAGS also contributed 96 samples to Phase 2 of the Thousand Genomes Project (TGP). Subsequently, BAGS samples were included in the NHLBI-supported parent grant, entitled New Approaches for Empowering Studies of Asthma in Populations of African Descent" (R01 HL104608-01), in which whole genome sequencing (WGS) was performed on ~1,000 individuals from North, Central, and South American and Caribbean and two West African populations. These populations constitute the Consortium on Asthma among African-ancestry Populations in the Americas (CAAPA), which aims to discover genes influencing risk for asthma, and catalog genetic diversity in descendants of the African Diaspora in the Americas. So far, CAAPA sequencing has greatly expanded the lexicon of human diversity, as we have observed >20% more variants than reported in the 1000 Genome Project (TGP). Using these WGS data, a custom, gene-centric SNP genotyping array was developed by Illumina, Inc., called the African Diaspora Power Chip (ADPC), to complement current, commercially available genome-wide chips, which provide sub-optimal tagging of genes among individuals of African ancestry. This ADPC was recently genotyped on all BAGS samples, with a goal of combining ADPC data with existing GWAS data from the 650Y to test for association with asthma. The initial goals of the parent grant did not include validating the ADPC. Moreover, the ADPC, combined with existing GWAS data, will be limited in detecting contributions of rare and structural variants, which may account for some of the "missing heritability" of asthma. We therefore are performing WGS on 1,100 asthmatics and family members from the BAGS, in order to (i) expand the CAAPA WGS dataset and thereby the genomic catalog of African ancestry for the research community; (ii) validate the ADPC by capturing information from both common and rare variants; and (iii) generate additional discovery of rare and structural variants that may control risk to asthma. Tools resulting from this study will result in substantial advancements in the technology available for identifying genes relevant to disease in under-represented minorities.

Given the data available on this large, deeply genotyped cohort from a relatively homogeneous environment representing an underrepresented minority group suffering most from asthma, the BAGS sample provides a unique opportunity to employ novel genomics.

We gratefully acknowledge the contributions of Pissamai and Trevor Maul, Paul Levett, Anselm Hennis, P. Michele Lashley, Raana Naidu, Malcolm Howitt and Timothy Roach, and the numerous health care providers, and community clinics and co-investigators who assisted in the phenotyping and collection of DNA samples, and the families and patients for generously donating DNA samples to the Barbados Asthma Genetics Study (BAGS). Funding for BAGS was provided by National Institutes of Health (NIH) R01HL104608, R01HL087699, and HL104608 S1.

BEAGESS (The Barrett's and Esophageal Adenocarcinoma. Genetic Susceptibility Study)
This study made use of data generated by investigators in the BEACON consortium through a grant funded by the US National Institutes of Health (NIH) (RO1CA136725) to Thomas L. Vaughan and David C. Whiteman (multiple PIs). In support of this work, T.L.V. was also supported by NIH grant KO5CA124911 and D.C.W. by a Future Fellowship grant FT0990987 from the Australia Research Council. Additional collaborators, sources of support and origin of the data and biospecimens are listed in the following publication: Levine DM, Ek WE, Zhang R, Liu X, Onstad L, Sather C, et al. A genome-wide association study identifies new susceptibility loci for esophageal adenocarcinoma and Barrett's esophagus. Nat Genet. 2013 Dec;45(12):1487-93. The dataset used for the analyses described in this manuscript was obtained from dbGaP at http://www.ncbi.nlm.nih.gov/gap through dbGaP accession number phs000869.v1.p1.

BLTS (Brisbane Longitudinal Twin Study)
The Brisbane Longitudinal Study acknowledges funding from the Australian National Health and Medical Research Council grants 496682, 1009064, 1009064, and 49662. Nathan A. Gillespie is supported by NIH R00DA023549, and Anna R. Docherty is supported by MH109765. For more information about this study, contact Nathan A. Gillespie (nathan.gillespie@vcuhealth.org).

CADD (Center on Antisocial Drug Dependence)
The Center on Antisocial Drug Dependence (CADD) data were funded by grants from the National Institute on Drug Abuse (P60 DA011015, R01 DA012845, R01 DA021913, R01 DA021905, R01 DA035804). For more information about this study, contact John K. Hewitt (john.hewitt@colorado.edu).

EOCOPD (Boston Early-Onset COPD Study)
The Boston Early-Onset COPD (EOCOPD) study was designed to study genetic factors for early-onset and severe COPD.14 Probands were selected to be physician-diagnosed COPD cases with FEV1≤ 40% predicted and age ≤ 53. Subjects with severe alpha-1 antitrypsin deficiency and other chronic lung diseases (except asthma) were excluded. All subjects completed a questionnaire and spirometry testing before and after bronchodilator administration. Blood samples and written informed consent were obtained for each study subject. A subset of the most severe unrelated probands from this study were sent for whole-genome sequencing through the TOPMed project. For the current cross-sectional WGS effort, only baseline spirometry data were available and utilized for analyses.

The Boston Early-Onset COPD Study was supported by R01 HL113264 and U01 HL089856 from the National Heart, Lung, and Blood Institute.

CFS (The Cleveland Family Study)

Obstructive Sleep Apnea (OSA) affects more than 10% of the population, especially minorities, and is associated with significant cardio-metabolic morbidity. We propose using data from the Cleveland Family Study (CFS), a genetic epidemiological study of OSA, as well as data from cohorts studied as part of our collaborations with other NHLBI cohorts to enhance the identification of genes that increase susceptibility to OSA, with a focus on those variants that increase susceptibility in African Americans.

The CFS is a genetic epidemiological study of 352 rigorously phenotyped families ascertained through probands with OSA identified through Cleveland, OH area sleep centers, neighborhood controls, and the spouses and first and second-degree relatives of probands. Participants were studied on up to 4 exams between 1990-2006 with overnight sleep studies, standardized anthropometry; questionnaires; blood pressure; and spirometry. Fasting serum and ECGs are available from the last exam. Participants have a mean age of 37.7 years (African Americans) and 41.4 years (European Americans). Slightly more than 50% of the sample is female and 31% have moderate to severe OSA; 12.6% have diabetes, and 34.0% have hypertension. Asthma is reported in 19% and 13% of the African Americans and European Americans, respectively. Heritability analysis of traditional OSA traits as well as novel traits such as hypopnea duration (a marker of respiratory arousability) as well as overnight oxygenation (a marker of susceptibility to hypoxemia occurring with recurrent apneas) has shown that the latter traits are more heritable ($h2 > 0.50$) than traditional measures. Linkage analysis has identified peaks (and individual families contributing to peaks) for these traits. Through the Life After Linkage initiative (5R01HL113338), we further have aggregated and analyzed data on 19,798 individuals from 7 cohorts (Cleveland Family Study [CFS] plus ARIC, FHS, HCHS/SOL, MESA, MrOS, and Starr County) and conducted the largest GWAS to date of OSA traits.

We used WGS and highly sophisticated statistical tools to completely characterize the genetic variation in richly phenotyped multi-ethnic populations and in families enriched for OSA as well as CVD and pulmonary traits. We aim to more completely and definitively characterize the allelic spectrum of functional genetic variation associated with OSA, as well as to contribute to consortia-wide activities to identify causal variation for other HLB phenotypes. We propose to conduct WGS in 1000 Cleveland Family Study family as well as to collaborate with other WGS consortium members (e.g., Jackson Heart Study) where sleep phenotyping is available. Complete characterization of genetic variation with WGS will allow for direct interrogation of causal functional variation irrespective of whether it is coding or regulatory; common, rare, private or de novo, thus improving upon data from exome sequencing. We will apply existing and newly developed analytical tools for detecting associations informed by linkage, and for conducting gene-based tests, bioinformatics pathway analyses, fine-mapping of GWAS and linkage signals using functional annotation, cross-phenotype analyses, and heritability partitioning to identify causal variants and reveal the allelic architecture of OSA, facilitating the discovery of physiological pathways. More comprehensive sequencing data, including a complete catalogue of genetic variation in each sequenced participant, will improve the ability to identify important inherited and de novo functional coding and regulatory variants outside of exomic regions for OSA, fine-map GWAS and linkage signals as well as will contribute to the discovery and fine-mapping of variants for a broad range of CVD, blood and pulmonary phenotypes collected in these cohorts. We will focus on the major metrics that characterize OSA such as the Apnea Hypopnea Index, as well as highly heritable traits that provide information on physiological mechanisms underlying OSA such as hypopnea duration as well as overnight oxygenation.

More information can be viewed at https://sleepdata.org/

The Cleveland Family Study has been supported in part by National Institutes of Health grants [R01-HL046380, KL2-RR024990, R35-HL135818, and R01-HL113338].

CHS (The Cardiovascular Health Study)

Cardiovascular Health Study (CHS) is a population-based, longitudinal study of risk factors for coronary heart disease and stroke (REF). The study included 5,888 adults 65 years of age or older from four field centers. Participants were sampled from local Medicare eligibility lists, and baseline visits were in 1989-90 for the first cohort (n=5,201) and 1992-94 for the second cohort (n=687, predominantly African-American). At each study visit, physical and laboratory evaluations were performed to identify the characterize the severity of cardiovascular disease risk factors. Blood samples for DNA extraction were from the baseline study visit. CHS was approved by institutional review committees at each field center and individuals in the present analysis had available DNA and gave informed consent including consent to use of genetic information for the study of cardiovascular disease.

Cardiovascular Health Study [SAG1]: This CHS research was supported by NHLBI contracts HHSN268201200036C, HHSN268200800007C, HHSN268201800001C, N01HC55222, N01HC85079, N01HC85080, N01HC85081, N01HC85082, N01HC85083, N01HC85086, 75N92021D00006; and NHLBI grants U01HL080295, R01HL087652, R01HL105756, R01HL103612, R01HL120393, and U01HL130114 with additional contribution from the National Institute of Neurological Disorders and Stroke (NINDS). Additional support was provided through R01AG023629 from the National Institute on Aging (NIA). A full list of principal CHS investigators and institutions can be found at CHS-NHLBI.org. The provision of genotyping data was supported in part by the National Center for Advancing Translational Sciences, CTSI grant UL1TR001881, and the National Institute of Diabetes and Digestive and Kidney Disease Diabetes Research Center (DRC) grant DK063491 to the Southern California Diabetes Endocrinology Research Center. The content is solely the responsibility of the authors and does not necessarily represent the official views of the National Institutes of Health.

COGEND (Collaborative Genetic Study of Nicotine Dependence)

This research was supported by P01 CA089392, U01 HG004422, and R01 DA036583. Funding support for genotyping which was performed at the Johns Hopkins University Center for Inherited Disease Research was provided by the NIH "Genome-wide Association Studies in the Genes and Environment Initiative" (U01HG004438) and the NIH contract "High throughput genotyping for studying the genetic contributions to human disease" (HHSN268200782096C). We also acknowledge funding from R01 DA026911, and we thank Weimin Duan for analytic assistance. For more information about this study, contact Laura J. Bierut (laura@wustl.edu).

COPDGene (Genetics of Chronic Obstructive Pulmonary Disease)

COPDGene (also known as the Genetic Epidemiology of COPD Study) is an NIH-funded, multicenter study. A study population of more than 10,000 smokers (1/3 African American and 2/3 non-Hispanic White) has been characterized with a study protocol including pulmonary function tests, chest CT scans, six minute walk testing, and multiple questionnaires. Five years after this initial visit, all available study participants are being brought back for a follow-up visit with a similar study protocol. This study has been used for epidemiologic and genetic studies. Previous genetic analysis in this study has been based on genome-wide SNP genotyping data.Approximately 1900 subjects will undergo whole genome sequencing in this NHLBI WGS project, including severe COPD subjects and resistant smoking controls. The COPDGene Study web site is: http://www.copdgene.org/ .

The COPDGene project described was supported by Award Number U01 HL089897 and Award Number U01 HL089856 from the National Heart, Lung, and Blood Institute. The content is solely the responsibility of the authors and does not necessarily represent the official views of the National Heart, Lung, and Blood Institute or the National Institutes of Health. The COPDGene project is also supported by the COPD Foundation through contributions made to an Industry Advisory Board comprised of AstraZeneca, Boehringer Ingelheim,

GlaxoSmithKline, Novartis, Pfizer, Siemens and Sunovion. A full listing of COPDGene investigators can be found at: http://www.copdgene.org/directory .

CRA_CAMP (The Genetic Epidemiology of Asthma in Costa Rica and the Childhood Asthma Management Program)

From February 2001 to August 2008, questionnaires were sent to the parents of 16,912 children (ages 6-14 years) enrolled in 140 Costa Rican schools; 9,180 (54.3%) questionnaires were returned. Children were eligible for the study if they had asthma (physician-diagnosed asthma and ≥ 2 respiratory symptoms or asthma attacks in the prior year) and a high probability of having ≥ 6 great-grandparents born in the Central Valley of Costa Rica (as determined by the study genealogist on the basis of the paternal and maternal last names of each of the child's parents). Of the 9,180 children screened, 3,113 (33.9%) had asthma. By the close of the recruitment and enrollment of the CRA Study in 2011, samples from 4,245 individuals have been collected for the main study and related subsequent studies. Children, parents and pedigree relatives gave blood samples for DNA extraction. All probands/children completed a protocol including questionnaires, spirometry, methacholine challenge testing (if their FEV1 was ≥ 65% of predicted), allergy skin testing, and collection of blood (for plasma, DNA and RNA extraction, and measurement of serum total and allergen-specific IgE) and house dust (for measurement of dust mite/cockroach allergens) samples.

The parent grant, R37 HL066289 was initially funded as an R01 and then was successfully renewed with a 1st percentile score and was then converted to an R37 MERIT Award that has subsequently been renewed. The grant is currently in its 13th year and has two years remaining in its current segment; thus, it is eligible for this administrative supplement.

The unique aspects of this population are that the asthma prevalence rates in Costa Rica (CR) are among the highest in the world and the Central Valley of Costa Rica, the primary recruitment/enrollment area, is a relative genetic isolate where we have had the ability to ascertain relatives in large extended pedigrees and phenotype these subjects for asthma and related traits. A total of 671 subjects in 8 large pedigrees were ascertained and phenotyped, and we have collected an additional 1053 trios. Initial efforts were focused on linkage studies and then we subsequently moved to genetic association studies, at which time we added the trios (N=720) in the Childhood Asthma Management Program (CAMP) to the grant as a replication population for our results, as these two populations have exactly the same study design (e.g., trios) and identical protocols for phenotyping subjects. We have approximately 100 phenotypes relevant to heart, lung, blood and sleep disorders, including asthma, obesity, height, COPD and blood and lipid disorders via metabolomics It is highly unlikely that any application has the ability to test private mutations in extended pedigrees, as well as generalize from inbred to outbred populations to the extent that we do in this proposal.

We have been productive in publishing our work. A total of 114 linkage and association papers have been written using these two study populations. Some of the most important findings from the initial 13 years of the study are the following: we have (1) identified MMP12 as an important gene for asthma, COPD and lung function decline; (2) identified allele specific chromatin remodeling via an insulator on chromosome 17 that co-regulates the ZPBP2/GSDMB/ORMDL3 locus; (3) identified, through linkage and fine- mapping, PRKCA as a novel gene for asthma and obesity; (4) replicated GWAS results for PDE4D from CAMP; (5) identified a novel gene for obesity, ROBO1; and (6) identified vitamin D deficiency as an important risk factor for increased asthma severity. In the third five-year cycle, we added GWAS and integrative genomics, including whole blood gene expression and methylation arrays on 384 probands. The current aims of the grant are to perform GWAS and association analysis on Costa Rica and replicate them in CAMP and other Hispanic populations. This aim is directly related with this administrative supplement. The GWAS analyses are just now being completed and the gene expression, microRNA and methylation arrays are just being run. This means that the aims of the parent

grant are directly aligned with the aims of the proposed administrative supplement. Therefore, with two years remaining on the parent grant, there is ample time to perform the whole genome sequencing proposed and report the results from the project. As described in the significance section of the administrative supplement.

deCODE (deCODE Genetics/AMGEN, Inc.)
The authors are thankful to the Icelandic participants and staff at the Patient Recruitment Center. The work at deCODE genetics / Amgen was supported in part by the National Institute of Drug Abuse (NIDA grants, R01-DA017932 and R01-DA034076). For more information about this study, email info@decode.is.

ECLIPSE (Evaluation of COPD longitudinally to Identify Predictive Surrogate Endpoints)
The "Evaluation of COPD Longitudinally to Identify Predictive Surrogate Endpoints" (ECLIPSE) study was a longitudinal, multicenter, observational investigation of 2164 COPD subjects and a smaller number of smoking controls (337) and nonsmoking controls (245) followed regularly for three years, with three chest CT scans (at baseline, one year, and three years) (Vestbo, European Respiratory Journal 2008; 31: 869). The COPD cases and smoking controls have been included in TOPMed. Inclusion criteria included age 40-75, at least 10 pack-years of smoking, and spirometry in GOLD grades 2-4 (COPD cases) or normal spirometry with post-bronchodilator FEV1>85% predicted and FEV1/FVC>0.7 (controls). Study visits were performed at enrollment, three months, and every six months thereafter with spirometry, questionnaires, and other clinical evaluations. The ECLIPSE CT scans have been analyzed with the VIDA software for emphysema and airway phenotypes. ECLIPSE has provided key insights into the clinical epidemiology of COPD, including COPD exacerbations (Hurst, NEJM 2010; 363: 1128) and lung function decline in COPD (Vestbo, NEJM 2011; 365: 1184). ECLIPSE has been used in a number of genetic studies of COPD susceptibility and protein biomarkers (Faner, Thorax 2014; 69: 666).

The ECLIPSE study (NCT00292552) was sponsored by GlaxoSmithKline. The ECLIPSE investigators included: ECLIPSE Investigators — Bulgaria: Y. Ivanov, Pleven; K. Kostov, Sofia. Canada: J. Bourbeau, Montreal; M. Fitzgerald, Vancouver, BC; P. Hernandez, Halifax, NS; K. Killian, Hamilton, ON; R. Levy, Vancouver, BC; F. Maltais, Montreal; D. O'Donnell, Kingston, ON. Czech Republic: J. Krepelka, Prague. Denmark: J. Vestbo, Hvidovre. The Netherlands: E. Wouters, Horn-Maastricht. New Zealand: D. Quinn, Wellington. Norway: P. Bakke, Bergen. Slovenia: M. Kosnik, Golnik. Spain: A. Agusti, J. Sauleda, P. de Mallorca. Ukraine: Y. Feschenko, V. Gavrisyuk, L. Yashina, Kiev; N. Monogarova, Donetsk. United Kingdom: P. Calverley, Liverpool; D. Lomas, Cambridge; W. MacNee, Edinburgh; D. Singh, Manchester; J. Wedzicha, London. United States: A. Anzueto, San Antonio, TX; S. Braman, Providence, RI; R. Casaburi, Torrance CA; B. Celli, Boston; G. Giessel, Richmond, VA; M. Gotfried, Phoenix, AZ; G. Greenwald, Rancho Mirage, CA; N. Hanania, Houston; D. Mahler, Lebanon, NH; B. Make, Denver; S. Rennard, Omaha, NE; C. Rochester, New Haven, CT; P. Scanlon, Rochester, MN; D. Schuller, Omaha, NE; F. Sciurba, Pittsburgh; A. Sharafkhaneh, Houston; T. Siler, St. Charles, MO; E. Silverman, Boston; A. Wanner, Miami; R. Wise, Baltimore; R. ZuWallack, Hartford, CT. ECLIPSE Steering Committee: H. Coxson (Canada), C. Crim (GlaxoSmithKline, USA), L. Edwards (GlaxoSmithKline, USA), D. Lomas (UK), W. MacNee (UK), E. Silverman (USA), R. Tal-Singer (Co-chair, GlaxoSmithKline, USA), J. Vestbo (Co-chair, Denmark), J. Yates (GlaxoSmithKline, USA). ECLIPSE Scientific Committee: A. Agusti (Spain), P. Calverley (UK), B. Celli (USA), C. Crim (GlaxoSmithKline, USA), B. Miller (GlaxoSmithKline, USA), W. MacNee (Chair, UK), S. Rennard (USA), R. Tal-Singer (GlaxoSmithKline, USA), E. Wouters (The Netherlands), J. Yates (GlaxoSmithKline, USA).

EGCUT (Estonian Genome Center)
The EGCUT studies were financed by Estonian Government (grants IUT20-60 and IUT24-6) and by European Commission through the European Regional Development Fund in the frame of grant Estonian Center of Genomics/Roadmap II (project No. 2014-2020.4.01.16-0125) and grant GENTRANSMED (Project No. 2014-2020.4.01.15-0012) and through H2020 grant no 692145 (ePerMed). For more information, please contact Tõnu Esko (tonu.esko@ut.ee).

eMERGE (Electronic Medical Records and Genomics)
Samples and associated genotype and phenotype data used in this study were provided by the Mayo Clinic. Funding support for the Mayo Clinic was provided through a cooperative agreement with the National Human Genome Research Institute (NHGRI), Grant #: UOIHG004599; and by grant HL75794 from the National Heart Lung and Blood Institute (NHLBI). Funding support for genotyping, which was performed at The Broad Institute, was provided by the NIH (U01HG004424). Assistance with phenotype harmonization and genotype data cleaning was provided by the eMERGE Administrative Coordinating Center (U01HG004603) and the National Center for Biotechnology Information (NCBI). The datasets used for analyses described in this manuscript were obtained from dbGaP at http://www.ncbi.nlm.nih.gov/gap through dbGaP accession number phs000203.v1.p1. Funding support for the Personalized Medicine Research Project (PMRP) was provided through a cooperative agreement (U01HG004608) with the National Human Genome Research Institute (NHGRI), with additional funding from the National Institute for General Medical Sciences (NIGMS) The samples used for PMRP analyses were obtained with funding from Marshfield Clinic, Health Resources Service Administration Office of Rural Health Policy grant number D1A RH00025, and Wisconsin Department of Commerce Technology Development Fund contract number TDF FYO10718. Funding support for genotyping, which was performed at Johns Hopkins University, was provided by the NIH (U01HG004438). Assistance with phenotype harmonization and genotype data cleaning was provided by the eMERGE Administrative Coordinating Center (U01HG004603) and the National Center for Biotechnology Information (NCBI). The datasets used for the analyses described in this manuscript were obtained from dbGaP at http://www.ncbi.nlm.nih.gov/gap through dbGaP accession number phs000360.v3.p1.

FHS (Framingham Heart Study)
Whole genome sequencing (WGS) in the Framingham Heart Study (FHS) would provide a unique opportunity to study the association of genome-wide genetic variation with heart, lung, blood, and sleep traits that are available in our six constituent cohorts (with nearly 15,000 participants). The Framingham Heart Study (FHS) acknowledges the support of contracts NO1-HC-25195, HHSN268201500001I and 75N92019D00031 from the National Heart, Lung and Blood Institute and grant supplement R01 HL092577-06S1 for this research. We also acknowledge the dedication of the FHS study participants without whom this research would not be possible. Dr. Vasan is supported in part by the Evans Medical Foundation and the Jay and Louis Coffman Endowment from the Department of Medicine, Boston University School of Medicine.

GeneSTAR (Genetic Studies of Atherosclerosis Risk)
GeneSTAR began in 1982 as the Johns Hopkins Sibling and Family Heart Study, a prospective longitudinal family-based study conducted originally in healthy adult siblings of people with documented early onset coronary disease under 60 years of age. Commencing in 2003, the siblings, their offspring, and the coparent of the offspring participated in a 2 week trial of aspirin 81 mg/day with pre and post ex vivo platelet function assessed using multiple agonists in whole blood and platelet rich plasma. Extensive additional cardiovascular testing and risk assessment was done at baseline and serially. Follow-up was carried out to determine incident cardiovascular disease, stroke, peripheral arterial disease, diabetes, cancer, and related comorbidities, from 5 to 30 years after study entry. The goal of several additional phenotyping and interventional substudies has been to discover and amplify understanding of the mechanisms of atherogenic vascular diseases and attendant comorbidities.

GeneSTAR was supported by the National Institutes of Health/National Heart, Lung, and Blood Institute (U01 HL72518, HL087698, HL112064) and by a grant from the National Institutes of Health/National Center for Research Resources (M01-RR000052) to the Johns Hopkins General Clinical Research Center.

GENOA (Genetic Epidemiology Network of Arteriopathy)
GENOA is one of four research networks that form the NHLBI Family Blood Pressure Program (FBPP). From its inception in 1995, GENOA's long-term objective was to elucidate the genetics of hypertension and its arteriosclerotic target-organ damage, including both atherosclerotic (macrovascular) and arteriolosclerotic (microvascular) complications involving the heart, brain, kidneys, and peripheral arteries. Two GENOA cohorts were originally ascertained (1995-2000) through sibships in which at least 2 siblings had essential hypertension diagnosed prior to age 60 years. All siblings in the sibship were invited to participate, both normotensive and hypertensive. These include non-Hispanic White Americans from Rochester, MN (n =1583 at the 1st exam) and African Americans from Jackson, MS (N=1854 at the 1st exam). During the second exam (2000-2005), approximately 80% of participants were re-recruited. The GENOA data consists of biological samples (DNA, serum, urine) as well as demographic, anthropometric, environmental, clinical, biochemical, physiological, and genetic data for understanding the genetic predictors of diseases of the heart, brain, kidney, and peripheral arteries.

Support for GENOA was provided by the National Heart, Lung and Blood Institute (HL054457, HL054464, HL054481, HL119443, and HL087660) of the National Institutes of Health. Whole genome sequencing (WGS) for the Trans-Omics in Precision Medicine (TOPMed) program was supported by the National Heart, Lung and Blood Institute (NHLBI). WGS for the TOPMed GENOA Study (phs001345.v1.p1) was performed at the Northwest Genomics Center at the University of Washington for the HyperGen/GENOA project (PI: Donna Arnett) and the Broad Institute of MIT and Harvard for the AACAC project (PI: Kent Taylor). Centralized read mapping and genotype calling, along with variant quality metrics and filtering were provided by the TOPMed Informatics Research Center (3R01HL-117626-02S1). Phenotype harmonization, data management, sample-identity QC, and general study coordination, were provided by the TOPMed Data Coordinating Center (3R01HL-120393-02S1). We gratefully acknowledge the studies and participants who provided biological samples and data for TOPMed.

GenSalt (Genetic Epidemiology Network of Salt Sensitivity)
The GenSalt study is aimed at identifying novel genes which interact with the effect of dietary sodium and potassium intake or cold pressor on blood pressure.

The Genetic Epidemiology Network of Salt-Sensitivity (GenSalt) was supported by research grants (U01HL072507, R01HL087263, and R01HL090682) from the National Heart, Lung and Blood Institute, National Institutes of Health, Bethesda, MD.

GfG (Genes for Good)
The Genes for Good study is funded through discretionary funds, provided to Dr. Gonçalo Abecasis by the University of Michigan. The authors sincerely thank all study participants for their time and dedication, as well as the hard-working Genes for Good administrative staff and colleagues at the UM DNA Sequencing Core. For more information about this study, see https://genesforgood.sph.umich.edu/ or contact the study directly at genesforgood@umich.edu.

GOLDN (Genetics of Lipid Lowering Drugs and Diet Network Lipidomics Study)
The GOLDN study was initiated to assess how genetic factors interact with environmental (diet and drug) interventions to influence blood levels of triglycerides and other atherogenic lipid species and inflammation markers (registered at clinicaltrails.gov, number NCT00083369). The study recruited Caucasian participants primarily from three-generational pedigrees from two NHLBI Family Heart Study (FHS) field centers (Minneapolis, MN and Salt Lake City, UT). Only families with at least two siblings were recruited and only participants who did not take lipid-lowering agents (pharmaceuticals or nutraceuticals) for at least 4 weeks prior

to the initial visit were included. A total of 1048 GOLDN participants were included in the diet intervention. The diet intervention followed the protocol of Patsch et al. (1992). The whipping cream (83% fat) meal had 700 Calories/m2 body surface area (2.93 MJ/m2 body surface area): 3% of calories were derived from protein (instant nonfat dry milk) and 14% from carbohydrate (sugar). The ratio of polyunsaturated to saturated fat was 0.06 and the cholesterol content of the average meal was 240 mg. The mixture was blended with ice and flavorings. Blood samples were drawn immediately before (fasting) and at 3.5 and 6 hours after consuming the high-fat meal. For the GOLDN lipidomics study, sterols and fatty acids were measured from stored plasma (-80 degrees Celsius) collected at fasting and 3.5 hours after the diet intervention using TrueMass Panels from Lipomics (West Sacramento, CA). A total of 11 sterols were quantified in nmols/gram of sample including total cholesterol, 7-dehydrocholesterol, desmosterol, lanosterol, lathasterol, cholestanol, coprostanol, beta-sitosterol, campesterol, stigmasterol, and 7alpha-hydroxycholesterol. A total of 35 fatty acids were quantified in nmols/gram of sample inlcuding myristic acid (14:0); pentadecanoic acid (15:0); palmitic acid (16:0); stearic acid (18:0); arachidic acid (20:0); behenic acid (22:0); lignoceric acid (24:0); myristoleic acid (14:1n5); palmitoleic acid (16:1n7); palmitelaidic acid (t16:1n7); oleic acid (18:1n9); elaidic acid (t18:1n9); vaccenic acid (18:1n7); linoleic acid (18:2n6); gamma-linolenic acid (18:3n6); alpha-linolenic acid (18:3n3); stearidonic acid (18:4n3); eicosenoic acid (20:1n9); eicosadienoic acid (20:2n6); mead acid (20:3n9); di-homo-gamma-linolenic acid (20:3n6); arachidonic acid (20:4n6); eicsoatetraenoic acid (20:4n3); eicosapentaenoic acid (20:5n3); erucic acid (22:1n9); docosadienoic acid (22:2n6); adrenic acid (22:4n6); docosapentaenoic acid (22:5n6); docosapentaenoic acid (22:5n3); docosahexaenoic acid (22:6n3); nervonic acid (24:1n9); and plasmalogen derivatives of 16:0, 18:0, 18:1n9, and 18:1n7.

GOLDN biospecimens, baseline phenotype data, and intervention phenotype data were collected with funding from National Heart, Lung and Blood Institute (NHLBI) grant U01 HL072524. Whole-genome sequencing in GOLDN was funded by NHLBI grant R01 HL104135 and supplement R01 HL104135-04S1.

HyperGEN_GENOA (Hypertension Genetic Epidemiology Network)
The Hypertension Genetic Epidemiology Network Study (HyperGEN) - Genetics of Left Ventricular (LV) Hypertrophy is a familial study aimed to understand genetic risk factors for LV hypertrophy by conducting genetic studies of continuous traits from echocardiography exams. The originating HyperGEN study aimed to understand genetic risk factors for hypertension. Data from detailed clinical exams as well as genotyping data for linkage studies, candidate gene studies and GWAS have been collected and is shared between HyperGEN and the ancillary HyperGEN - Genetics of LV Hypertrophy study.

HyperGEN recruited 470 multiply-affected population-based hypertensive AA sibships (N=1224 siblings) from 1996-1999. HyperGEN probands were ascertained by early onset hypertension (i.e., before 60 years); to participate, they had to have at least one hypertensive sibling who was also willing to participate. Hypertension was defined according to antihypertensive treatment or BP (systolic BP ≥140 or diastolic BP ≥90 measured on at least at two time points); Type 1 diabetes and renal failure were exclusion criteria. The HyperGEN study extended recruitment to include all offspring (n=546) of the proband and his/her siblings during the second 5-year funding period. HyperGEN also recruited participants randomly selected from the source cohorts where the families were ascertained to represent population allele frequencies; 414 African American participants were recruited randomly. In total, ~2,200 African-American participants were examined and gave permission for DNA testing and sharing from sites in Birmingham, AL and Winston-Salem, NC. Of those ~2000 have data recorded from an echocardiography exam and were included in the TOPMed study of LV hypertrophy and related traits.

The HyperGEN Study is part of the National Heart, Lung, and Blood Institute (NHLBI) Family Blood Pressure Program; collection of the data represented here was supported by grants U01 HL054472 (MN Lab), U01 HL054473 (DCC), U01 HL054495 (AL FC), and U01 HL054509 (NC FC). The HyperGEN: Genetics of Left

Ventricular Hypertrophy Study was supported by NHLBI grant R01 HL055673 with whole-genome sequencing made possible by supplement -18S1.

NHS, NHS2, and HPFS (Nurses' Health Study, Nurses' Health Study II, and Health Professionals' Follow-up Study)

The contributions from the Nurses' Health Study, Nurses Health Study II, and Health Professionals' Follow-up Study were supported by the National Institute of Health grants P01CA87969, P01CA055075, P01DK070756, U01HG004728, UM1CA186107, UM1CA176726, UM1CA167552, R01CA49449, R01CA50385, R01CA67262, R01HL034594, R01HL088521, R01HL35464, R01EY015473, R01EY022305, P30EY014104, R03DC013373, and R03CA165131. For information about these studies, contact Peter Kraft pkraft@hsph.harvard.edu) or Marilyn C. Cornelis (marilyn.cornelis@northwestern.edu).

HCHS_SOL (Hispanic Community Health Study-Study of Latinos)

The Hispanic Community Health Study / Study of Latinos (HCHS/SOL) is a multi-center epidemiologic study in Hispanic/Latino populations to determine the role of acculturation in the prevalence and development of disease, and to identify risk factors playing a protective or harmful role in Hispanics/Latinos. The study is sponsored by the National Heart, Lung, and Blood Institute (NHLBI) and six other institutes, centers, and offices of the National Institutes of Health (NIH).

The goals of the HCHS/SOL include studying the prevalence and development of disease in Hispanics/Latinos, including the role of acculturation, and identifying disease risk factors that play protective or harmful roles in Hispanics/Latinos. A total of 16,415 persons of Cuban, Dominican, Mexican, Puerto Rican, Central American, and South American backgrounds were recruited through four Field Centers affiliated with San Diego State University, Northwestern University in Chicago, Albert Einstein College of Medicine in the Bronx area of New York, and the University of Miami. Seven additional academic centers serve as scientific and logistical support centers.

Study participants aged 18-74 years took part in an extensive clinic exam and assessments to ascertain socio-demographic, cultural, environmental and biomedical characteristics. Annual follow-up interviews are conducted to determine a range of health outcomes.

We thank the participants and staff of the Hispanic Community Health Study/Study of Latinos (HCHS/SOL) for their contributions to this study. The baseline examination of HCHS/SOL was carried out as a collaborative study supported by contracts from the NHLBI to the University of North Carolina (N01-HC65233), University of Miami (N01- HC65234), Albert Einstein College of Medicine (N01-HC65235), Northwestern University (N01-HC65236), and San Diego State University (N01-HC65237). The following institutes, centers, and offices contributed to the first phase of HCHS/SOL through a transfer of funds to the NHLBI: National Institute on Minority Health and Health Disparities, National Institute on Deafness and Other Communication Disorders, National Institute of Dental and Craniofacial Research (NIDCR), National Institute of Diabetes and Digestive and Kidney Diseases (NIDDK), National Institute of Neurological Disorders and Stroke, and NIH Office of Dietary Supplements. The Genetic Analysis Center at the University of Washington was supported by NHLBI and NIDCR contracts (HHSN268201300005C AM03 and MOD03). Additional analysis support was provided by 1R01DK101855-01 and 13GRNT16490017. Genotyping efforts were supported by the NIH Department of Health and Human Services (HSN26220/20054C), National Center for Advancing Translational Science Clinical Translational Science Institute (UL1TR000124), and NIDDK Diabetes Research Center (DK063491).

HRS (Health and Retirement Study)

HRS is supported by the National Institute on Aging (NIA U01AG009740). The genotyping was funded separately by the National Institute on Aging (RC2 AG036495, RC4 AG039029). Our genotyping was conducted by the NIH Center for Inherited Disease Research (CIDR) at Johns Hopkins University. Genotyping quality control and final preparation of the data were performed by the University of Michigan School of Public Health. See the HRS website (http://hrsonline.isr.umich.edu/gwas) for details.

HUNT (The Nord-Trøndelag Health Study)

HUNT is a collaboration between HUNT Research Centre (Faculty of Medicine, NTNU, Norwegian University of Science and Technology), the Nord-Trøndelag County Council, the Central Norway Health Authority, and the Norwegian Institute of Public Health. Bendik Slagsvold Winsvold is funded by the University of Oslo and the South Eastern Norway Health Trust. Anne Heidi Skogholt, Maiken Elvestad Gabrielsen, and Kristian Hveem are funded by NTNU, the Central Norway Health Trust, and the K.J. Jebsen Foundation. For more information about this study, email hunt@medisin.ntnu.no.

HVH (Heart and Vascular Health Study)

The Heart and Vascular Health Study (HVH) is a case-control study of risk factors for the development of myocardial infarction (MI), stroke, venous thrombosis (VT), and atrial fibrillation (AF). The study setting is Group Health, an integrated health care delivery system in Washington State.

The HVH originated in 1988 with the examination of risk factors for MI. Over the ensuing years, the study has been funded by a series of grants, which have added case subjects with stroke, VT, and AF, and used a common control group. Study aims have focused on the associations of medication use with cardiovascular events. Starting in 1997, the study aims expanded to include genetic associations with cardiovascular disease. Participants recruited in 2009 or later and who provided blood samples for genetic analysis were asked for consent to deposit genetic and phenotype data in dbGaP.

As part of the HVH study, case subjects were identified by searching for ICD-9 codes consistent with MI, stroke, VT, or AF, and medical records were reviewed to confirm the diagnosis. Control subjects were identified at random from the Group Health enrollment and were matched to MI cases. All subjects have an index date. For cases, the index date was assigned as the date that the cardiovascular event (MI, stroke, VT, or AF) came to clinical attention. For controls, the index date was a random date within the range of the case index dates. For both cases and controls, information was collected from the inpatient and outpatient medical record, by telephone interview with consenting survivors, and from the Group Health pharmacy and laboratory databases. Consenting participants provided a blood specimen.

The Heart and Vascular Health Study was supported by grants HL068986, HL085251, HL095080, and HL073410 from the National Heart, Lung, and Blood Institute.

IPF (Whole Genome Sequencing in Familiar and Sporadic Idiopathic Pulmonary Fibrosis)

This is a set of cases diagnosed with idiopathic pulmonary fibrosis, a fatal interstitial lung disease. These cases were included in the TOPMed phase three studies. The planned study will compare these cases to within-TOPMed controls for genome-wide association studies.

JHS (Jackson Heart Study)

The Jackson Heart Study (JHS) is supported and conducted in collaboration with Jackson State University (HHSN268201800013I), Tougaloo College (HHSN268201800014I), the Mississippi State Department of Health (HHSN268201800015I) and the University of Mississippi Medical Center (HHSN268201800010I, HHSN268201800011I and HHSN268201800012I) contracts from the National Heart, Lung, and Blood Institute

(NHLBI) and the National Institute on Minority Health and Health Disparities (NIMHD). The authors also wish to thank the staffs and participants of the JHS. The views expressed in this manuscript are those of the authors and do not necessarily represent the views of the National Heart, Lung, and Blood Institute; the National Institutes of Health; or the U.S. Department of Health and Human Services.

Whole genome sequencing (WGS) for the Trans-Omics in Precision Medicine (TOPMed) program was supported by the National Heart, Lung and Blood Institute (NHLBI). WGS for "NHLBI TOPMed: Jackson Heart Study" (phs000964) was performed at the Northwest Genomics Center (HHSN268201100037C). Core support including centralized genomic read mapping and genotype calling, along with variant quality metrics and filtering were provided by the TOPMed Informatics Research Center (3R01HL-117626-02S1; contract HHSN268201800002I). Core support including phenotype harmonization, data management, sample-identity QC, and general program coordination were provided by the TOPMed Data Coordinating Center (R01HL-120393; U01HL-120393; contract HHSN268201800001I). We gratefully acknowledge the studies and participants who provided biological samples and data for TOPMed.

LMR was additionally supported by KL2TR002490 from the National Center for Advancing Translational Sciences (NCATS).

LLS (WHI Long Life Study)
7,875 WHI Extension II Study participants participated in the WHI Long Life Study (LLS). The LLS consisted of a one-time in-person visit (conducted between March 2012 and May 2013) with a blood draw, a brief clinical assessment, and an assessment of functional status.

MCTFR (Minnesota Center for Twin and Family Research)
MCTFR was supported in part by USPHS Grants from the National Institute on Alcohol Abuse and Alcoholism (R01 AA09367 and R01 AA11886) and from the National Institute on Drug Abuse (R01 DA05147, R01 DA13240, R01DA042755, U01DA046413, R21DA046188, R01DA037904, R01HG008983, and U01 DA024417). GWAS and phenotypic data for MCTFR subjects who provided consent to place their data in a public repository are deposited into the database of Genotypes and Phenotypes (dbGaP, www.ncbi.nlm.nih.gov/gap) under phs000620. For further information, please contact Matt McGue (mcgue001@umn.edu).

MESA (Multi-Ethnic Study of Atherosclerosis) and MESA Family African-American Coronary Artery Calcium consortium study (AA_CAC)
Whole genome sequencing (WGS) for the Trans-Omics in Precision Medicine (TOPMed) program was supported by the National Heart, Lung and Blood Institute (NHLBI). WGS for "NHLBI TOPMed: Multi-Ethnic Study of Atherosclerosis (MESA)" (phs001416.v1.p1) was performed at the Broad Institute of MIT and Harvard (3U54HG003067-13S1). Centralized read mapping and genotype calling, along with variant quality metrics and filtering were provided by the TOPMed Informatics Research Center (3R01HL-117626-02S1, contract HHSN268201800002I). Phenotype harmonization, data management, sample-identity QC, and general study coordination, were provided by the TOPMed Data Coordinating Center (3R01HL-120393; U01HL-120393; contract HHSN268180001I).

MESA and the MESA SHARe projects are conducted and supported by the National Heart, Lung, and Blood Institute (NHLBI) in collaboration with MESA investigators. Support for MESA is provided by contracts 75N92020D00001, HHSN268201500003I, N01-HC-95159, 75N92020D00005, N01-HC-95160, 75N92020D00002, N01-HC-95161, 75N92020D00003, N01-HC-95162, 75N92020D00006, N01-HC-95163, 75N92020D00004, N01-HC-95164, 75N92020D00007, N01-HC-95165, N01-HC-95166, N01-HC-95167, N01-HC-95168, N01-HC-95169, UL1-TR-000040, UL1-TR-001079, and UL1-TR-001420, UL1TR001881, DK063491, and R01HL105756. Funding

for SHARe genotyping was provided by NHLBI Contract N02-HL-64278. Genotyping was performed at Affymetrix (Santa Clara, California, USA) and the Broad Institute of Harvard and MIT (Boston, Massachusetts, USA) using the Affymetrix Genome-Wide Human SNP Array 6.0. The authors thank the other investigators, the staff, and the participants of the MESA study for their valuable contributions. A fill list of participating MESA investigators and institutes can be found at http://www.mesa-nhlbi.org.

METSIM (Metabolic Syndrome in Men Study)
T2D-GENES (Type 2 Diabetes Genetic Exploration by Next-Generation Sequencing in Multi-Ethnic Samples) is a NIDDK-funded international research consortium which seeks to identify genetic variants for type 2 diabetes (T2D) through multiethnic sequencing studies. T2D-GENES Project 1 is a multi-ethnic sequencing study designed to assess whether less common variants play a role in T2D risk and to assess similarities and differences in the distribution of T2D risk variants across ancestry groups.

The individuals were obtained from 14 cohorts that are listed in Table 1. The strategy was to perform deep exome sequencing of 12,940 individuals, 6,504 with T2D and 6,436 controls, divided among five ancestry groups: Europeans, East Asians, South Asians, American Hispanics, and African Americans. Sequencing was performed at the Broad Institute using the Agilent v2 capture reagent on Illumina HiSeq machines. Please note that while we summarize the full sample list in publications and below, the Kooperative Gesundheitsforschung in der Region Augsburg (KORA) study does not have a sub study, as it is not consented to be deposited in dbGAP.

NESCOG (Netherlands Study on Cognition, Environment and Genes)
This research was part of Science Live, the innovative research program of science center NEMO that enables scientists to carry out real, publishable, peer-reviewed research using NEMO visitors as volunteers. P.R. Jansen was supported by the Sophia Foundation for Scientific Research (#S14-27).

FinnTwin & NAG-FIN (Finnish Twin Cohort)
The Finnish Twin Cohort/Nicotine Addiction Genetics-Finland study was supported by Academy of Finland (grants # 213506, 129680 to JK), NIH DA12854 (Pamela A F Madden), Global Research Award for Nicotine Dependence / Pfizer Inc. (JK), Wellcome Trust Sanger Institute, UK and the European Community's Seventh Framework Programme ENGAGE Consortium (HEALTH-F4-2007- 201413). In Finntwin12, support for data collection and genotyping has come from National Institute of Alcohol Abuse and Alcoholism (grants AA-12502, AA-00145, and AA-09203 to RJR and AA15416 and K02AA018755 to Danielle M Dick), the Academy of Finland (grants 100499, 205585, 118555, 141054 and 264146 to JK) & Wellcome Trust Sanger Institute, UK. JK has been supported by the Academy of Finland (grants # 265240 & 263278) and the Sigrid Juselius Foundation. For more information about this study, contact Jaakko Kaprio (jaakko.kaprio@helsinki.fi).

NTR (Netherlands Twin Register)
NTR would like to thank all the twins and family members for their participation. This work was supported by the Netherlands Organization for Scientific Research (NWO: MagW/ZonMW grants 904-61-090, 985-10-002,904-61-193,480-04-004, 400-05- 717, Addiction-31160008 Middelgroot-911-09-032, Spinozapremie 56-464-14192), Biobanking and Biomolecular Resources Research Infrastructure (BBMRI –NL, 184.021.007), the VU University's Institute for Health and Care Research (EMGO+) and Neuroscience Campus Amsterdam (NCA), the European Science Council (ERC Advanced, 230374), the Avera Institute for Human Genetics, Sioux Falls, South Dakota (USA) and the National Institutes of Health (NIH, R01D0042157-01A). Part of the genotyping was funded by the Genetic Association Information Network (GAIN) of the Foundation for the US National Institutes of Health (NIMH, MH081802) and by the Grand Opportunity grants 1RC2MH089951-01 and 1RC2 MH089995-01 from the NIMH. Part of the analyses were carried out on the Genetic Cluster Computer (http://www.geneticcluster.org), which is financially supported by the Netherlands Scientific Organization (NWO 480-05-003), the Dutch Brain

Foundation, and the department of Psychology and Education of the VU University Amsterdam. For access to NTR results, please contact Dorret I. Boomsma at di.boomsma@vu.nl.

OMG_SCD (Outcome Modifying Genes in Sickle Cell Disease)

Sickle cell disease (SCD) is caused by homozygosity for a single mutation of the beta hemoglobin gene. Despite the constancy of this genetic abnormality, the clinical course of patients with SCD is remarkably variable. SCD can affect the function and cause the failure of multiple organ systems through the pathophysiologic processes of vaso-occlusion and hemolysis. These pathophysiological processes are complex and expected to impact multiple organ systems in a variety of ways. This study, therefore, was designed to identify genetic factors that predispose SCD patients to develop specific end-organ complications and to experience more or less severe clinical courses. We enrolled > 700 patients with Hb SS, Hb S-beta0 thalassemia and HbSC being followed primarily at three southeastern U.S. regional institutions (Duke University Medical Center, University of North Carolina Medical Center, and Emory University Medical Center). Medical information obtained included the presence or absence of specific targeted outcomes (overall disease severity as well as specific types of end organ damage). Clinical data include medical status (history, physical, examination, and laboratory results) and information regarding potentially confounding environmental factors. Limited plasma samples are available for correlative studies (e.g. of cytokine levels, coagulation activation). Targeted SNP for candidate gene analysis as well as GWAS has been performed on most samples. Whole genome sequencing has been conducted through the TOPMed Consortium. The subjects in this analysis were collected as part of a larger study, "Outcome Modifying Genes in Sickle Cell Disease" (OMG-SCD) aimed at identifying genetic modifiers for sickle cell disease. More information about the study can be found in Elmariah et al. (2014), PMID: 24478166. Clinical and genetic data have been used to identify genetic characteristics predisposing patients with SCD to a more or less severe overall clinical course as well as to individual organ-specific complications. It is anticipated that identification of such genetic factors will reveal new therapeutic targets individualized to specific complications of SCD, leading to improved outcomes and increased life expectancy for patients with SCD.

The OMG-SCD study was administrated by Marilyn J. Telen, M.D. and Allison E. Ashley-Koch, Ph.D. from Duke University Medical Center and collection of the data set was supported by grants HL068959 and HL079915 from the National Heart, Lung, and Blood Institute (NHLBI) of the National Institute of Health (NIH).

OZALC (Australian Twin-Family Studies on Nicotine and Alcohol Genetics)

OZALC acknowledges the work over many years of staff of the Genetic Epidemiology group at QIMR Berghofer Medical Research Institute (formerly the Queensland Institute of Medical Research) in managing the studies which generated the data used in this analysis. We also acknowledge and appreciate the willingness of study participants to complete multiple, and sometimes lengthy, questionnaires and interviews. Many of the participants were contacted originally through the Australian Twin Registry. Funding for the original studies in which information on alcohol use and smoking status was obtained came from the US National Institutes of Health (AA07535, AA07728, AA11998, AA13320, AA13321, AA14041, AA17688, DA012854 and DA019951); the Australian National Health and Medical Research Council (241944, 339462, 389927, 389875, 389891, 389892, 389938, 442915, 442981, 496739, 552485 and 552498); and the Australian Research Council (A7960034, A79906588, A79801419, DP0770096, DP0212016 and DP0343921). Researchers interested in using this dataset may apply for access using dbGaP Study Accession phs000181.v1.p1.

BLTS (Brisbane Longitudinal Twin Study)

The Brisbane Longitudinal Study acknowledges funding from the Australian National Health and Medical Research Council grants 496682, 1009064, 1009064, and 49662. Nathan A. Gillespie is supported by NIH R00DA023549, and Anna R. Docherty is supported by MH109765. For more information about this study, contact Nathan A. Gillespie (nathan.gillespie@vcuhealth.org).

SAFS (San Antonio Family Studies)
Population of SAFS include Mexican American in SAFHS extended pedigrees. The San Antonio Family Heart Study (SAFHS) is a complex pedigree-based mixed longitudinal study designed to identify low frequency or rare variants influencing susceptibility to cardiovascular disease, using whole genome sequence (WGS) information from 2,590 individuals in large Mexican American pedigrees from San Antonio, Texas. The major objectives of this study are to identify low frequency or rare variants in and around known common variant signals for CVD, as well as to find novel low frequency or rare variants influencing susceptibility to CVD.

WGS of the SAFHS cohort has been obtained through three efforts. Approximately 540 WGS were performed commercially at 50X by Complete Genomics, Inc (CGI) as part of the large T2D-GENES Project. The phenotype and genotype data for this group is available at dbGaP under accession number phs000462. An additional ~900 WGS at 30X were obtained through Illumina as part of the R01HL113322 "Whole Genome Sequencing to Identify Causal Genetic Variants Influencing CVD Risk" project. Finally, ~1,150 WGS at 30X WGS were obtained through Illumina funded by a supplement as part of the NHLBI's TOPMed program.

Extensive phenotype data are provided for sequenced individuals primarily obtained from the P01HL45522 "Genetics of Atherosclerosis in Mexican Americans" for adults and R01HD049051 for children in these same families. Phenotype information was collected between 1991 and 2016. For this dataset, the SAFHS appellation represents an amalgamation of the original SAFHS participants and an expansion that reexamined families previously recruited for the San Antonio Family Diabetes Study (R01DK042273) and the San Antonio Family Gall Bladder Study (R01DK053889). Due to this substantial examination history, participants may have information from up to five visits. The clinical variables reported are coordinated with TOPMed and include major adverse cardiac events (MACE), T2D status and age at diagnosis, glycemic traits (fasting glucose and insulin), blood pressure, blood lipids (total cholesterol, HDL cholesterol, calculated LDL cholesterol and triglycerides). Additional phenotype data include the medication status at each visit, classified in four categories as any current use of diabetes, hypertension or lipid-lowering medications, and, for females, current use of female hormones. Anthropometric measurements include age, sex, height, weight, hip circumference, waist circumference and derived ratios. PBMC derived gene expression assays for a subset of ~1,060 individuals obtained using the Illumina Sentrix-6 chip is also available from the baseline examination. The WGS data have been jointly called and are available in the current TOPMed accession (phs001215).

Collection of the San Antonio Family Study data was supported in part by National Institutes of Health (NIH) grants R01 HL045522, MH078143, MH078111 and MH083824; and whole genome sequencing of SAFS subjects was supported by U01 DK085524 and R01 HL113323. We are very grateful to the participants of the San Antonio Family Study for their continued involvement in our research programs.

SardiNIA (SardiNIA project)
We thank all the volunteers who generously participated in this study and made this research possible. This research was supported by National Human Genome Research Institute grants HG005581, HG005552, HG006513, HG007022 and HG007089; by National Heart, Lung, and Blood Institute grant HL117626; by the Intramural Research Program of the US National Institutes of Health, National Institute on Aging, contracts N01-AG-1-2109 and HHSN271201100005C; by Sardinian Autonomous Region (L.R. 7/2009) grant cRP3-154; by the PB05 InterOmics MIUR Flagship Project; by grant FaReBio2011 'Farmaci e Reti Biotecnologiche di Qualità'. For information about this study, contact David Schlessinger (SchlessingerD@mail.nih.gov) or Francesco Cucca (fcucca@irgb.cnr.it).

SARP (Severe Asthma Research Program)

SARP is the world's most comprehensive study of adults and children with severe asthma, linking 7 leading asthma clinical university centers and 1 data coordinating center through a National Institutes of Health-sponsored network. SARP is not a clinical trial but rather an intensive characterization study of adults and children with asthma. Now in its third phase (SARP III), SARP III has enrolled over 700 participants in its program, including over 500 adults and 180 children aged 6-17 years.
The SARP network's mission is to improve the understanding of severe asthma in order to develop better treatments. Through SARP, we are gaining insight into how severe asthma develops in patients and learning about the molecular, cellular and biological mechanisms that lead to different types of asthma. Participants enrolled in SARP are being followed over at least three years in order to determine how these characteristics develop or change with time.

The overall goal of the Severe Asthma Research Program (SARP) is to identify and characterize subjects with severe asthma to understand pathophysiologic mechanisms in severe asthma. Subjects with mild and moderate asthma were recruited for comparison but the program was enriched for subjects with severe asthma from multiple centers. Subjects were comprehensively phenotyped for asthma related traits including lung function, atopy, questionnaires on medical and family history, exhaled nitric oxide and health care utilization including exacerbations and symptoms. Asthma is a heterogenous disease. Cluster analysis in SARP has shown multiple subphenotypes and endotypes.

Samoan (Samoan Adiposity Study)
The parent Samoan Adiposity Study ("Samoan", formerly "SAS") is a population-based genome-wide association study (GWAS) of adiposity and cardiometabolic phenotypes among adults from the independent nation of Samoa in the South Pacific. The research goal of this study is to identify genetic variation that increases susceptibility to obesity and cardiometabolic phenotypes. Over 3,400 individuals ages 25-65 years were recruited in 2010 from 33 villages from all census regions of the nation, which is experiencing economic development and the nutrition transition. Eligibility was based on self-report of having four Samoan grandparents, not being pregnant, and not having severe physical impairment which would prohibit collection of anthropometric, biomarker and questionnaire measures, nor cognitive impairment which would not allow informed consent about the genetic purposes of the study. We collected overnight fasting blood samples and assayed glucose, insulin, leptin, adiponectin, total cholesterol, high-density and low-density lipoprotein cholesterol, and triglycerides. Anthropometric and bioelectrical impedance measurements provided measures of weight, height, body circumferences, skinfold thicknesses, BMI and other indices, as well as estimation of percent body fat and lean tissue. Questionnaires assessed socio-demographic characteristics, physical activity, dietary intake using food frequency questionnaires, medication use, history of prior diagnoses of type 2 diabetes, hypertension and cardiovascular disease, and alcohol and tobacco use. DNA was collected and the Affymetrix 6.0 chip used for SNP genotyping. After quality control checks on genotyping and excluding individuals with key missing data we have a final sample of 3,122 adults with high-quality genome-wide marker data.

Participation in the NHLBI TOPMed WGS project will enable us to more thoroughly investigate the genetic architecture of Samoan cardiometabolic conditions by establishing a Samoan-specific reference panel for imputation. Specifically, the NHLBI TOPMed WGS project will perform whole-genome sequencing in an optimally-selected subset of more than 400 individuals from our GWAS sample. After quality control work, we will use this Samoan reference panel to impute genotypes for the rest of our discovery sample. Using the imputed genotypes, we will carry out association analyses for each of our cardiometabolic traits, primarily using gene-based association tests.

Date collection was funded by NIH grant R01-HL093093. We thank the Samoan participants of the study and local village authorities. We acknowledge the support of the Samoan Ministry of Health and the Samoa Bureau of Statistics for their support of this research.

NINDS SiGN (The National Institute of Neurological Disorders and Stroke Genetics Network)

The NINDS International Stroke Genetics Consortium Study dataset was funded by the National Institute of Neurological Disorders and Stroke Cooperative Agreement Award 1U01NS069208 (Steven Kittner). The dataset used for the analyses described in this manuscript was obtained from dbGaP at http://www.ncbi.nlm.nih.gov/gap through dbGaP accession number phs000615.v1.p1.

THRV (Taiwan Study of Hypertension using Rare Variants)

The THRV-TOPMed study consists of three cohorts: The SAPPHIRe Family cohort (N=1,271), TSGH (Tri-Service General Hospital, a hospital-based cohort, N=160), and TCVGH (Taichung Veterans General Hospital, another hospital-based cohort, N=922), all based in Taiwan. 1,271 subjects were previously recruited as part of the NHLBI-sponsored SAPPHIRe Network (which is part of the Family Blood Pressure Program, FBPP). The SAPPHIRe families were recruited to have two or more hypertensive sibs, some families also with one normotensive/hypotensive sib. The two Hospital-based cohorts (TSGH and TCVGH) both recruited unrelated subjects with different recruitment criteria (matched with SAPPHIRe subjects for age, sex, and BMI category).

The Rare Variants for Hypertension in Taiwan Chinese (THRV) is supported by the National Heart, Lung, and Blood Institute (NHLBI) grant (R01HL111249) and its participation in TOPMed is supported by an NHLBI supplement (R01HL111249-04S1). THRV is a collaborative study between Washington University in St. Louis, LA BioMed at Harbor UCLA, University of Texas in Houston, Taichung Veterans General Hospital, Taipei Veterans General Hospital, Tri-Service General Hospital, National Health Research Institutes, National Taiwan University, and Baylor University. THRV is based (substantially) on the parent SAPPHIRe study, along with additional population-based and hospital-based cohorts. SAPPHIRe was supported by NHLBI grants (U01HL54527, U01HL54498) and Taiwan funds, and the other cohorts were supported by Taiwan funds.

UKB (UK Biobank)

This research has been conducted using the UK Biobank Resource under Application Number 16651. Informed consent was obtained from UK Biobank subjects.

VTE (Venous Thromboembolism project)

This study consists of 338 VTE cases from an inception cohort of Olmsted County, MN residents (OC) with a first lifetime objectively-diagnosed idiopathic VTE during the 40-year study period, 1966-2005. All living study subjects were invited to provide a whole blood sample at the Mayo Clinical Research Unit for leukocyte genomic DNA and plasma collection. For living study subjects who did not provide a blood sample, we retrieved any leftover blood ("waste" blood) from samples collected as part of routine clinical diagnostic testing and used this to extract DNA after obtaining patient consent. For deceased cases, with IRB approval, we extracted DNA from any available stored tissue within the Mayo Tissue Archive. This "tissue" DNA has been successfully genotyped in prior studies. Three trained and experienced study nurse abstractors reviewed the complete medical records in the community of all potential cases.

Funded, in part, by grants from the National Institutes of Health, National Heart, Lung and Blood Institute (HL66216 and HL83141). the National Human Genome Research Institute (HG04735, HG06379), and research support provided by Mayo Foundation.

WGHS (The Women's Genome Health Study)

The Women's Genome Health Study (WGHS) is a prospective cohort comprised of over 25,000 initially healthy female health professionals enrolled in the Women's Health Study, which began in 1992-1994. All participants in WGHS provided baseline blood samples and extensive survey data. Women who reported atrial fibrillation during the course of the study were asked to report diagnoses of AF at baseline, 48 months, and then annually thereafter. Participants enrolled in the continued observational follow-up who reported an incident AF event on at least one yearly questionnaire were sent an additional questionnaire to confirm the episode and to collect additional information. They were also asked for permission to review their medical records, particularly available ECGs, rhythm strips, 24-hour ECGs, and information on cardiac structure and function. For all deceased participants who reported AF during the trial and extended follow-up period, family members were contacted to obtain consent and additional relevant information. An end-point committee of physicians reviewed medical records for reported events according to predefined criteria. An incident AF event was confirmed if there was ECG evidence of AF or if a medical report clearly indicated a personal history of AF. The earliest date in the medical records when documentation was believed to have occurred was set as the date of onset of AF.

The WGHS is supported by the National Heart, Lung, and Blood Institute (HL043851 and HL080467) and the National Cancer Institute (CA047988 and UM1CA182913), with funding for genotyping provided by Amgen. The most recent cardiovascular endpoints were supported by ARRA funding HL099355.

WHI and sub-studies (Women's Health Initiative Clinical Trial and Observational Study)
The Women's Health Initiative (WHI) is a long-term national health study that has focused on strategies for preventing heart disease, breast and colorectal cancer, and osteoporotic fractures in postmenopausal women. The original WHI study included 161,808 postmenopausal women enrolled between 1993 and 1998. The Fred Hutchinson Cancer Research Center in Seattle, WA serves as the WHI Clinical Coordinating Center for data collection, management, and analysis of the WHI.

The WHI has two major parts: a partial factorial randomized Clinical Trial (CT) and an Observational Study (OS); both were conducted at 40 Clinical Centers nationwide.

We also include some sub-studies of WHI include GECCO (Genetics & Epidemiology of Colorectal Cancer Consortium), GARNET (GWAS of Hormone Treatment and CVD and Metabolic Outcomes in the WHI) and MOPMAP.

Genetics & Epidemiology of Colorectal Cancer Consortium (GECCO) is a collaborative effort which aims to accelerate the discovery of colorectal cancer-related variants by discovering, replicating and fine-mapping Genome Wide Association Study (GWAS) findings, conducting a meta-analysis of GWAS data, and investigating how genetic variants are modified by environmental risk factors. The coordinating center for this consortium is based at the Fred Hutchinson Cancer Research Center (Principal investigator: Ulrike Peters).
This study is part of the Genomics and Randomized Trials Network (GARNET, http://www.garnetstudy.org) funded by the National Human Genome Research Institute (NHGRI). The overarching goal is to identify novel genetic factors that contribute to incidence of myocardial infarction, stroke, venous thrombosis, and diabetes through large-scale genome-wide association studies of treatment response in a randomized clinical trial of hormone therapy. Genotyping was performed at the Broad Institute of MIT and Harvard. Data cleaning and harmonization were performed at the GARNET Coordinating Center at the University of Washington.
Participants were selected as a nested case-control sample of coronary heart disease, stroke, venous thrombosis, and incident diabetes events from the parent WHI Hormone Trial.

WHI GARNET participants are women enrolled in the WHI Hormone Therapy (HT) Trial who meet eligibility requirements for this study and eligibility for submission to dbGaP, and who provided DNA samples. Of the approximately 27,000 women who participated in the HT trial, 4,894 were genotyped on the Illumina Omni-Quad as part of WHI GARNET, a genome-wide association study (GWAS) to identify genetic components involved in differential responses from the HT trial. Case selection of adjudicated phenotypes of interest included coronary heart disease, stroke, venous thrombosis, and incident diabetes cases that occurred during the active phase of the Hormone Trial (HT). Controls were participants in the HT trial free of all 4 case conditions by the end of the trials. HT participants with treated diabetes at baseline were excluded from the diabetes cases and controls pool, but were still considered for cases of other 3 conditions. Matching criteria for controls were age, race/ethnicity, hysterectomy status, and enrollment date. GARNET WHI participants belong to the following self-identified ethnic groups: white (87%), black (5%), Hispanic (3%), Asian/Pacific Islander (1.8%), and American Indian (0.7%). The ethnic group is unknown for 1.9% of participants.

The WHI program is funded by the National Heart, Lung, and Blood Institute, National Institutes of Health, U.S. Department of Health and Human Services through contracts HHSN268201600018C, HHSN268201600001C, HHSN268201600002C, HHSN268201600003C, and HHSN268201600004C. Personal funding for Sean P. David from National Institute on Minority Health and Health Disparities grant U54-MD010724. The authors thank the WHI investigators and staff for their dedication, and the study participants for making the program possible. A full listing of WHI investigators can be found at: http://www.whi.org/researchers/Documents Write a Paper/WHI Investigator Short List.pdf. For more information about this study, please contact nm9o@nih.gov.

The WHI program is funded by the National Heart, Lung, and Blood Institute, National Institutes of Health, U.S. Department of Health and Human Services through contracts 75N92021D00001, 75N92021D00002, 75N92021D00003, 75N92021D00004, 75N92021D00005.

REFERENCE

1. Mowinckel, A.M. & Vidal-Piñeiro, D. Visualization of Brain Statistics With R Packages ggseg and ggseg3d. *Advances in Methods and Practices in Psychological Science* **3**, 466-483 (2020).
2. Tobacco & Genetics, C. Genome-wide meta-analyses identify multiple loci associated with smoking behavior. *Nat Genet* **42**, 441-7 (2010).
3. Gamazon, E.R. *et al.* A gene-based association method for mapping traits using reference transcriptome data. *Nat Genet* **47**, 1091-8 (2015).
4. Markunas, C.A. *et al.* Genome-wide DNA methylation differences in nucleus accumbens of smokers vs. nonsmokers. *Neuropsychopharmacology* **46**, 554-560 (2021).
5. Stegle, O., Parts, L., Piipari, M., Winn, J. & Durbin, R. Using probabilistic estimation of expression residuals (PEER) to obtain increased power and interpretability of gene expression analyses. *Nat Protoc* **7**, 500-7 (2012).
6. Shabalin, A.A. Matrix eQTL: ultra fast eQTL analysis via large matrix operations. *Bioinformatics* **28**, 1353-8 (2012).
7. Bryois, J. *et al.* Genetic identification of cell types underlying brain complex traits yields insights into the etiology of Parkinson's disease. *Nat Genet* **52**, 482-493 (2020).
8. Han, B. & Eskin, E. Random-effects model aimed at discovering associations in meta-analysis of genome-wide association studies. *Am J Hum Genet* **88**, 586-98 (2011).
9. Fiddes, I.T. *et al.* Human-Specific NOTCH2NL Genes Affect Notch Signaling and Cortical Neurogenesis. *Cell* **173**, 1356-1369 e22 (2018).
10. Fuke, S., Sasagawa, N. & Ishiura, S. Identification and characterization of the Hesr1/Hey1 as a candidate trans-acting factor on gene expression through the 3' non-coding polymorphic region of the human dopamine transporter (DAT1) gene. *J Biochem* **137**, 205-16 (2005).
11. Chastagner, P., Rubinstein, E. & Brou, C. Ligand-activated Notch undergoes DTX4-mediated ubiquitylation and bilateral endocytosis before ADAM10 processing. *Sci Signal* **10**(2017).
12. Delaney, A., Keighren, M., Fleetwood-Walker, S.M. & Jackson, I.J. Involvement of the melanocortin-1 receptor in acute pain and pain of inflammatory but not neuropathic origin. *PLoS One* **5**, e12498 (2010).
13. Chaki, S., Ogawa, S., Toda, Y., Funakoshi, T. & Okuyama, S. Involvement of the melanocortin MC4 receptor in stress-related behavior in rodents. *Eur J Pharmacol* **474**, 95-101 (2003).
14. Serova, L.I., Laukova, M., Alaluf, L.G. & Sabban, E.L. Intranasal infusion of melanocortin receptor four (MC4R) antagonist to rats ameliorates development of depression and anxiety related symptoms induced by single prolonged stress. *Behav Brain Res* **250**, 139-47 (2013).
15. Chaffin, A.T., Fang, Y., Larson, K.R., Mul, J.D. & Ryan, K.K. Sex-dependent effects of MC4R genotype on HPA axis tone: implications for stress-associated cardiometabolic disease. *Stress* **22**, 571-580 (2019).
16. Sabban, E.L. & Serova, L.I. Potential of Intranasal Neuropeptide Y (NPY) and/or Melanocortin 4 Receptor (MC4R) Antagonists for Preventing or Treating PTSD. *Mil Med* **183**, 408-412 (2018).
17. Tapinc, D.E. *et al.* Gene expression of pro-opiomelanocortin and melanocortin receptors is regulated in the hypothalamus and mesocorticolimbic system following nicotine administration. *Neurosci Lett* **637**, 75-79 (2017).
18. Uhl, G.R. *et al.* Cocaine reward is reduced by decreased expression of receptor-type protein tyrosine phosphatase D (PTPRD) and by a novel PTPRD antagonist. *Proc Natl Acad Sci U S A* **115**, 11597-11602 (2018).
19. de Leeuw, C.A., Mooij, J.M., Heskes, T. & Posthuma, D. MAGMA: generalized gene-set analysis of GWAS data. *PLoS Comput Biol* **11**, e1004219 (2015).
20. Subramanian, A. *et al.* Gene set enrichment analysis: a knowledge-based approach for interpreting genome-wide expression profiles. *Proc Natl Acad Sci U S A* **102**, 15545-50 (2005).
21. Liberzon, A. *et al.* Molecular signatures database (MSigDB) 3.0. *Bioinformatics* **27**, 1739-40 (2011).

22. Supek, F., Bosnjak, M., Skunca, N. & Smuc, T. REVIGO summarizes and visualizes long lists of gene ontology terms. *PLoS One* **6**, e21800 (2011).
23. Gu, Z. & Hübschmann, D. simplifyEnrichment: an R/Bioconductor package for Clustering and Visualizing Functional Enrichment Results. *bioRxiv*, 2020.10.27.312116 (2020).
24. Lin, D. An Information-Theoretic Definition of Similarity. in *Proceedings of the Fifteenth International Conference on Machine Learning* 296–304 (Morgan Kaufmann Publishers Inc., 1998).
25. Kuznetsova, I., Lugmayr, A., Siira, S.J., Rackham, O. & Filipovska, A. CirGO: an alternative circular way of visualising gene ontology terms. *BMC Bioinformatics* **20**, 84 (2019).
26. Kanehisa, M. & Goto, S. KEGG: kyoto encyclopedia of genes and genomes. *Nucleic Acids Res* **28**, 27-30 (2000).
27. Jassal, B. *et al.* The reactome pathway knowledgebase. *Nucleic Acids Res* **48**, D498-D503 (2020).
28. Martens, M. *et al.* WikiPathways: connecting communities. *Nucleic Acids Res* **49**, D613-D621 (2021).
29. Wishart, D.S. *et al.* DrugBank 5.0: a major update to the DrugBank database for 2018. *Nucleic Acids Res* **46**, D1074-D1082 (2018).
30. Sakaue, S. & Okada, Y. GREP: genome for REPositioning drugs. *Bioinformatics* **35**, 3821-3823 (2019).
31. Yu, A.Z. & Ramsey, S.A. A Computational Systems Biology Approach for Identifying Candidate Drugs for Repositioning for Cardiovascular Disease. *Interdiscip Sci* **10**, 449-454 (2018).
32. Wakefield, J. Bayes factors for genome-wide association studies: comparison with P-values. *Genet Epidemiol* **33**, 79-86 (2009).
33. Pickrell, J.K. Joint analysis of functional genomic data and genome-wide association studies of 18 human traits. *Am J Hum Genet* **94**, 559-73 (2014).
34. Mahajan, A. *et al.* Fine-mapping type 2 diabetes loci to single-variant resolution using high-density imputation and islet-specific epigenome maps. *Nat Genet* **50**, 1505-1513 (2018).
