# Supplementary material for: Multi-ancestry transcriptome-wide association analyses yield insights into tobacco use biology and drug repurposing
Source: Nat Genet. 2023 Jan 26;55(2):291–300. doi: 10.1038/s41588-022-01282-x (PMC9925385; doi:10.1038/s41588-022-01282-x)
Supplement: Supplementary file 2 — Reporting Summary [file 41588_2022_1282_MOESM2_ESM.pdf]

Corresponding author(s): Dajiang Liu, Bibo Jiang, and Scott Vrieze

Last updated by author(s): Jun 2, 2022

Reporting Summary

Nature Portfolio wishes to improve the reproducibility of the work that we publish. This form provides structure for consistency and transparency in reporting. For further information on Nature Portfolio policies, see our Editorial Policies and the Editorial Policy Checklist.

Statistics

For all statistical analyses, confirm that the following items are present in the figure legend, table legend, main text, or Methods section.

n/a	Confirmed	
☐	☒	The exact sample size (*n*) for each experimental group/condition, given as a discrete number and unit of measurement
☐	☒	A statement on whether measurements were taken from distinct samples or whether the same sample was measured repeatedly
☐	☒	The statistical test(s) used AND whether they are one- or two-sided<br>*Only common tests should be described solely by name; describe more complex techniques in the Methods section.*
☐	☒	A description of all covariates tested
☐	☒	A description of any assumptions or corrections, such as tests of normality and adjustment for multiple comparisons
☐	☒	A full description of the statistical parameters including central tendency (e.g. means) or other basic estimates (e.g. regression coefficient) AND variation (e.g. standard deviation) or associated estimates of uncertainty (e.g. confidence intervals)
☐	☒	For null hypothesis testing, the test statistic (e.g. *F*, *t*, *r*) with confidence intervals, effect sizes, degrees of freedom and *P* value noted<br>*Give P values as exact values whenever suitable.*
☒	☐	For Bayesian analysis, information on the choice of priors and Markov chain Monte Carlo settings
☒	☐	For hierarchical and complex designs, identification of the appropriate level for tests and full reporting of outcomes
☐	☒	Estimates of effect sizes (e.g. Cohen's *d*, Pearson's *r*), indicating how they were calculated

Our web collection on statistics for biologists contains articles on many of the points above.

Software and code

Policy information about availability of computer code

Data collection	No software was used in data collection.
Data analysis	TESLA is implemented and made available in https://github.com/funfunchen/rareGWAMA (DOI: 10.5281/zenodo.7352120).<br>Other softwares used included: MAGMA (v1.08, https://ctg.cncr.nl/software/magma), REVIGO (accessed 2022/05; http://revigo.irb.hr/), CirGO (accessed 2022/05; https://github.com/IrinaVKuznetsova/CirGO), METASOFT (v2.0.1; http://zarlab.cs.ucla.edu/software/), MetaXcan (v0.7.1; https://github.com/hakyimlab/MetaXcan), R Shiny (v1.7.2), MAMBA (v1.12; https://github.com/dan11mcguire/mamba), ggseg (v1.5.3; https://github.com/ggseg/ggseg).

For manuscripts utilizing custom algorithms or software that are central to the research but not yet described in published literature, software must be made available to editors and reviewers. We strongly encourage code deposition in a community repository (e.g. GitHub). See the Nature Portfolio guidelines for submitting code & software for further information.

Data

Policy information about availability of data

All manuscripts must include a data availability statement. This statement should provide the following information, where applicable:

- Accession codes, unique identifiers, or web links for publicly available datasets
- A description of any restrictions on data availability
- For clinical datasets or third party data, please ensure that the statement adheres to our policy

Pre-computed gene expression prediction model weights of 48 tissues are from the PrediXcan website (GTEx v7): https://predictdb.org/.
Gene Ontology (GO) and pathway gene sets are from MSigDB (https://www.gsea-msigdb.org/gsea/msigdb/).
RNA-seq and genotype data from postmortem nucleus accumbens samples of physiologically normal human brains are from Lieber Institute for Brain Development

(LIBD) Human Brain Repository Data (http://eqtl.brainseq.org/phase2/eqtl/).
Single cell RNA-seq data from entire mouse nervous system is from https://github.com/jbryois/scRNA_disease.
GWAS datasets from the GWAS and Sequencing Consortium of Alcohol and Nicotine use (GSCAN) and the Trans-Omics Precision Medicine (TOPMed).

Field-specific reporting

Please select the one below that is the best fit for your research. If you are not sure, read the appropriate sections before making your selection.

☒ Life sciences ☐ Behavioural & social sciences ☐ Ecological, evolutionary & environmental sciences

For a reference copy of the document with all sections, see nature.com/documents/nr-reporting-summary-flat.pdf

Life sciences study design

All studies must disclose on these points even when the disclosure is negative.

Sample size	In this study, we conducted a multi-ancestry TWAS using GWAS data from the GSCAN study and whole genome sequence from TOPMed with a total sample size of 1.3 million individuals (1.2 million from the GSCAN and 150,000 from the TOPMed).
Data exclusions	There is no data exclusions in our analysis. All datasets that pass quality controls are analyzed.
Replication	We use MAMBA to assess the GWAS association signals which is a model based method to assess whether the signal is genuine or not without a replication dataset. MAMBA works by examining the strength and consistency of association signals across studies.
Randomization	This is re-analysis of existing datasets coming from observational studies. No new data is collected. No randomization is performed here.
Blinding	This is re-analysis of existing datasets coming from observational studies. No new data is collected. No randomization is performed here.

Reporting for specific materials, systems and methods

We require information from authors about some types of materials, experimental systems and methods used in many studies. Here, indicate whether each material, system or method listed is relevant to your study. If you are not sure if a list item applies to your research, read the appropriate section before selecting a response.

Materials & experimental systems

n/a	Involved in the study	
☒	☐	Antibodies
☒	☐	Eukaryotic cell lines
☒	☐	Palaeontology and archaeology
☒	☐	Animals and other organisms
☒	☐	Human research participants
☒	☐	Clinical data
☒	☐	Dual use research of concern

Methods

n/a	Involved in the study	
☒	☐	ChIP-seq
☒	☐	Flow cytometry
☒	☐	MRI-based neuroimaging
